# Nucleotide Metabolism and Immune Genes Can Predict the Prognostic Risk of Hepatocellular Carcinoma and the Immune Microenvironment

**DOI:** 10.3390/biology14081079

**Published:** 2025-08-18

**Authors:** Xiaofang Wang, Qinghua Cui, Yuan Zhou

**Affiliations:** 1 Department of Biomedical Informatics, School of Basic Medical Sciences, Peking University, Beijing 100191, China; 2311110086@stu.pku.edu.cn (X.W.); cuiqinghua@bjmu.edu.cn (Q.C.); 2 Department of Pathogenic Biology and Immunology, Department of Basic Medicine, School of Medicine, Shihezi University, Shihezi 832000, China; 3 School of Sports Medicine, Wuhan Institute of Physical Education, Wuhan 430079, China

**Keywords:** hepatocellular carcinoma, nucleotide metabolism, immune microenvironment, prognostic risk, biomarker

## Abstract

The overall survival of hepatocellular carcinoma (HCC) remains poor, highlighting the need for better prognostic tools. Nucleotide metabolism fuels tumor progression, while the immune microenvironment dictates therapy response, but integrated models combining both features are lacking. Using TCGA-LIHC transcriptomic/clinical data, we identified nucleotide metabolism and immune-related differentially expressed genes (NMIRGs), which stratified HCC patients into two subtypes via non-negative matrix factorization. A nine-gene prognostic risk signature was constructed through LASSO/Cox regression and validated using independent GEO datasets, and the NMIRG signature was further validated experimentally via RT-qPCR in HCC cell lines and independently using the HPA database for protein-level evidence. As evaluated by our risk signature, high-risk patients exhibited altered immune profiles (T cells increasing, neutrophils decreasing), elevated tumor mutation burden and microsatellite instability, and worse predicted immunotherapy response. Gene set enrichment analysis linked high-risk genes to immune pathways and low-risk genes to metabolic processes. Our risk signature predicted HCC prognosis independent of demographic features and outperformed existing signatures with superior C-index accuracy, effectively predicting immune microenvironment status and therapy benefits. Together, this integrated NMIRG signature offers enhanced prognostication and identifies promising biomarkers for personalized HCC management.

## Simple Summary

Hepatocellular carcinoma (HCC) has a poor prognosis, necessitating better risk prediction tools. While nucleotide metabolism fuels tumor growth and immune evasion, and the immune microenvironment dictates therapy response, existing prognostic models typically focus on only one aspect. This study developed an integrated prognostic signature combining nucleotide metabolism and immune-related genes (NMIRGs) using TCGA-LIHC data. We identified two HCC subtypes (C1: poor prognosis, high immune infiltration; C2: better prognosis) based on NMIRG profiles. A nine-gene NMIRG signature (high-risk: HSP90AA1, HDAC1, RAC3, STC1, MAPT, BTC, CHGA, and GAL; low-risk: GHR) was constructed and validated in independent GEO datasets. The risk score was an independent prognostic factor, correlating with advanced stage, specific immune checkpoint expression, altered immune cell infiltration (e.g., increased T cells, decreased neutrophils in high-risk), higher tumor mutation burden (TMB), and microsatellite instability (MSI). The model showed potential for predicting immunotherapy response differences. Crucially, it outperformed existing single-feature models in predicting survival (higher C-index). Validated across multiple datasets and supplemented with experimental evidence, this NMIRG signature provides a superior tool for HCC risk stratification and immune microenvironment assessment, offering insights for personalized management and biomarker discovery.

## 1. Introduction

Hepatocellular carcinoma (HCC) is the third leading cause of cancer-related deaths worldwide, with a 5-year relative survival rate of approximately 18% and up to 830,000 cases of morbidity and mortality each year [1]. In recent years, although many novel therapies have been proposed and developed, individual survival rates remain low due to delays in diagnosis, frequent metastasis, and high recurrence rates [2]. The pathological diagnosis of HCC is usually based on the examination of resected or explant specimens, or biopsy specimens; however, it is worth noting that up to 9% of advanced HCC patients receive inappropriate treatment in the absence of biopsy [3]. Consequently, deciphering key molecular determinants of HCC progression and treatment response is critical for improving prognosis. Early identification of high-risk HCC patients enables tailored surveillance and interventions, highlighting the urgent need for robust prognostic biomarkers and models to enhance risk prediction accuracy and guide clinical decisions.

The extensive heterogeneity of HCC complicates biomarker discovery. Dysregulated nucleotide metabolism plays a pivotal role in tumorigenesis, profoundly impacting proliferation, metastasis, and therapeutic resistance [4]. As the central hub for nucleotide synthesis, the liver is particularly vulnerable; metabolic perturbations directly impair function and drive oncogenesis [5]. Furthermore, enhanced nucleotide metabolism is critical for cancer initiation and progression downstream of oncogene activation [6,7]. Therefore, developing and validating prognostic prediction models of HCC involving nucleotide metabolism-related genes holds significant promise for refining HCC prognostication.

Simultaneously, the tumor immune microenvironment (TIME) decisively influences HCC behavior and therapy response. While immunotherapy (e.g., ICB) shows promise [1,8], its efficacy is limited by immunosuppressive TIME features that promote resistance [9]. Crucially, emerging evidence reveals profound crosstalk between nucleotide metabolism and immune function: aberrant metabolism in cancer cells actively sculpts an immunosuppressive TIME, facilitating immune evasion [4,7,10]. Aberrant nucleotide metabolism in cancer cells not only fuels tumor growth but also actively shapes the TIME, often suppressing anti-tumor immunity and fostering an environment conducive to immune evasion [4]. However, current prognostic models focus narrowly on single dimensions—either immune-related genes (IRGs) [11,12] or nucleotide metabolism-related genes (NMRGs) [13,14]—overlooking this critical interaction and limiting their clinical utility.

To overcome this limitation, we hypothesize that integrating nucleotide metabolism and immune regulation will yield a more powerful prognostic tool. Therefore, our study aims to: (1) identify HCC molecular subtypes based on integrated nucleotide metabolism and immune-related DEG profiles; (2) develop and validate a multi-gene prognostic signature (NMIRGs) combining these DEGs with clinical features; (3) characterize the NMIRG signature’s association with TIME, genomic instability, biological pathways (GSEA), and immunotherapy response; and (4) evaluate its prognostic performance against existing single-feature models. Using the TCGA-LIHC cohort for discovery and independent GEO datasets (GSE14520/GSE10141/GSE27150) for validation, this integrated model aims to provide superior risk stratification and novel insights into HCC’s immune-metabolic landscape. To bridge prediction and clinical relevance, we experimentally validated signature gene expression via RT-qPCR and leveraged the HPA database for independent protein-level assessment. We anticipate that this integrated prognostic model will provide clinicians with a superior tool for risk stratification and offer insights into the immune-metabolic landscape of HCC, facilitating personalized management strategies and identifying potential novel biomarkers.

## 2. Materials and Methods

### 2.1. Data Acquisition and Processing

RNA sequencing (RNA-seq) transcriptome, clinical information, and somatic mutation data were obtained from The Cancer Genome Atlas database (TCGA; https://portal.gdc.cancer.gov/; accessed on 20 September 2024), including 374 LIHC and 50 normal samples. External validation datasets of HCC patients were obtained from the TCGA database and the Gene Expression Omnibus (GEO; https://www.ncbi.nlm.nih.gov/geo/; accessed on 21 September 2024) database, including GSE14520 (GPL3721 subset), GSE10141, and GSE27150. The data were normalized, and differentially expressed genes (DEGs) of the samples were analyzed using the “limma” package (v3.64.3) of R (v4.4.2). The DEG extraction criteria were as follows: |log2Fold-Change| > 1 and *p* < 0.05. All gene expression values were treated as log2-scale. When multiple probes corresponded to the same gene symbol, the mean expression was calculated as the final expression value. 

### 2.2. DEG Subset Extraction and Non-Negative Matrix Factorization (NMF) Clustering

From the MSigDB database (https://www.gsea-msigdb.org/gsea/msigdb/; accessed on 21 September 2024), “c2.cp.kegg.v7.4” contained 947 metabolism-related genes downloaded from the KEGG pathway set. From these, 72 genes specifically related to nucleotide metabolism were identified using the DAVID database [15] (https://david.ncifcrf.gov/tools.jsp; accessed on 21 September 2024). Immune-related genes (1793 genes after removing duplicates) were obtained from the ImmPort database [16] (https://www.immport.org/home; accessed on 21 September 2024).The transcriptome data of the TCGA database were intersected to obtain differentially expressed genes (DEGs) related to nucleotide metabolism and immunity. Then, the expression of DEGs in each tumor sample in the TCGA database was combined with clinical data by univariate Cox analysis to obtain the expression of prognosis-related NMIRGs. The “nsNMF” algorithm of the “NMF” package (v0.23.0) was used to cluster HCC patient samples according to the expression of NMIRGs, and the cluster number K was set between 2 and 10. With the “survival” package (v3.8-3), survival analysis of HCC patients was conducted, and the progression-free survival (PFS) of HCC patients was validated using the TCGA Pan-Cancer Atlas (https://pancanatlas.xenahubs.net; accessed on 25 September 2024) pan-cancer data.

### 2.3. Analysis of the Tumor Microenvironment and the Immune Infiltration

Stromal cell and immune cell scores in HCC were calculated using the Estimation of STromal and Immune cells in MAlignant Tumor tissues using Expression data (ESTIMATE) [17] software (v1.0.13), which uses the unique properties of transcriptional profiles to infer the properties of tumor cells as well as the purity of tumors, thereby predicting the levels of immune infiltration. The MCPcounter [18] algorithm estimated the abundance of each immune microenvironment-related cell type in the different HCC subtypes. The “MCPcounter” package (v1.1.0) provides abundance scores for T cells, CD8 T cells, cytotoxic lymphocytes, B lineage, NK cells, monocytic lineage, myeloid dendritic cells, neutrophils, endothelial cells, and fibroblasts for each sample.

### 2.4. Prognostic Model Construction and Validation

Following reference [19], the model was constructed by randomly splitting the samples into training and test sets in a 7:3 ratio within the TCGA-LIHC cohort. Samples without complete survival data were excluded, and 259 samples (70%) out of 371 TCGA-LIHC datasets were used as the training set (TCGAtrain set), while 112 samples (30%) were used as the internal test set (TCGAtest set). A Lasso regression model was constructed using the “glmnet” (v4.1-8) and “caret” (v7.0-1) R packages. Nine NMIRG signature genes were identified to construct a prognostic risk score model, and high-risk and low-risk groups were divided according to the median risk score of each sample. HCC samples in GSE14520 and GSE10141 were used as the external validation set (GEOtest set). Univariate Cox regression analysis was performed on the DEGs in the GSE14520 dataset using survival time and status to identify prognostic NMIRGs. The model was verified by survival analysis, ROC curves at 1, 3, and 5 years using the “rms” package (v8.0-0), decision curve analysis (DCA), and risk curves. Survival differences in Kaplan–Meier curves were compared using the log-rank test. The ROC curve and the area under the curve (AUC) were obtained using the “timeROC” package (v0.4).

### 2.5. Evaluation and Comparison of the Prognostic Models

Differences in clinical characteristics between the TCGAtest and TCGAtrain groups were analyzed using the chi-squared test. Correlation analysis was performed using Pearson’s method, and the Mann–Whitney U test was used to compare differences between the two groups. Clinical data metrics were integrated using logistic regression to generate a survival prediction nomogram. Independent prognostic factors were analyzed by univariate and multivariate Cox risk regression analyses using the “survival” package (v3.8-3). The model performance was evaluated by correlation analysis of immune checkpoint genes (31 genes of HCC [20,21]) and immune infiltration in high-risk and low-risk groups of HCC patients. The TCIA database [22] (https://tcia.at/home; accessed on 27 September 2024) was used to analyze the immunotherapeutic effect of *PD-1* and *CTLA-4* in HCC. Gene set enrichment analysis (GSEA) was performed to explore active functions or pathways and the underlying mechanisms. Correlation analysis of risk score, microsatellite instability (MSI), TMB, and tumor immune microenvironment was conducted to evaluate the prognostic value of this model. Finally, we compared our model with the previous models reported in the references, and the accuracy of our model was evaluated by the C-index value. All statistical analyses in this study were performed using R software (version 4.3.1); *p* < 0.05 indicated statistical significance.

### 2.6. Detection of the Expression of NMRIG Signature

HuH-7 and MHCC97-H cells were obtained from the Cell Collection Committee of the Chinese Academy of Sciences (Shanghai, China). The expression of NMIRG signature genes was detected by real-time PCR. After 24 h of culture of HuH-7 and MHCC97-H cells, total RNA of HCC cells was extracted using a total RNA extraction kit (Thermo Fisher Scientific Inc., Waltham, MA, USA). Then, the total RNA was reverse transcribed into cDNA using a reverse transcription kit (Thermo Fisher Scientific Inc., Waltham, MA, USA), and the relative gene expression was detected by quantitative fluorescent PCR (Bioer, Hangzhou, China). The primer sequences are shown in Supplementary Table S1. Protein expression levels of these genes in HCC tissue were retrieved from the Human Protein Atlas online database (HPA, v24.0, https://www.proteinatlas.org/; accessed on 4 February 2025). In the immunohistochemical (IHC) test, the samples of HCC patients were obtained from the TCGA database, where the degree of antibody staining was classified as not detected, low, medium, or high. A positive result was defined as tissue stained dark brown.

## 3. Results

### 3.1. NMIRGs Clustered HCC Patients into Two Subtypes, and They Showed Significant Differences in Tumor Microenvironment and Immune Infiltration

According to our procedure, a total of 431 DEGs were found by combining the NMIRGs with the TCGA transcriptome data (|log2 fold-change| > 1, *p* < 0.05), of which 380 were up-regulated DEGs and 51 were down-regulated DEGs (Supplementary Figure S1A,B; Supplementary Table S2). Among them, there were 382 immune-related genes, 34 metabolism-related genes, and 3 crossover genes (Supplementary Figure S1C). These DEGs distinguished HCC samples from normal controls (Supplementary Figure S2; Supplementary Table S2). Using univariate Cox regression analysis and NMF clustering, HCC was divided into C1 and C2 subtypes (Supplementary Figures S1D and S3; Supplementary Table S3). The Kaplan–Meier curve showed that the overall survival (OS) and PFS of C1 were significantly lower than those of C2 (*p* < 0.05, Supplementary Figure S1E,F), indicating that nucleotide metabolism and immune DEGs (NMIRGs) discriminate between different types of HCC.

**Figure 1:**
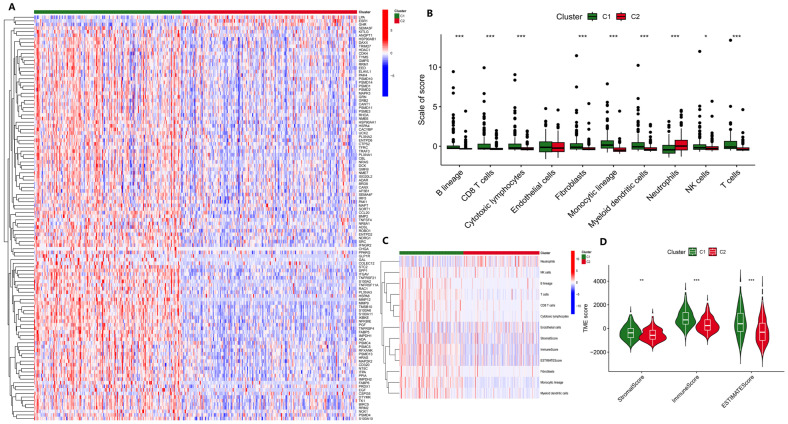
Analysis of the immune microenvironment in different HCC subtypes. (**A**) Comparison of DEGs of two HCC subtypes by unsupervised clustering. (**B**) Comparison of the results for the two HCC subtypes using the MCPcounter algorithm. (**C**) Heatmap of the immune cell scoring using the MCPcounter algorithm. (**D**) Comparison of the tumor microenvironment of the two HCC subtypes using the ESTIMATE algorithm. * *p* < 0.05, ** *p* < 0.01, *** *p* < 0.001.

Studies have shown that nucleotide metabolism is associated with the immune microenvironment [7], so we speculate that NMIRGs may also distinguish immune infiltration between HCC subtypes. The unsupervised clustering diagram shows that nucleotide metabolism and immune DEGs showed higher expressions in C1 than in C2 (Figure 1A). Using MCPcounter for eight immune microenvironment-related cell types for analysis, we found that, except for the neutrophils and endothelial cells, all other cells were significantly increased in subtype C1 (*p* < 0.05), whereas neutrophils were significantly increased in subtype C2 (Figure 1B), indicating that immune cells were mainly infiltrated in the C1 tumor samples. Similarly, the results of tumor microenvironment analysis also showed that immune cells scored higher in C1 (Figure 1C, D). Both the score and the composite score of stromal cells were higher in C1 than in C2, indicating that the tumor purity of C1 was lower than that of C2. Combined with the previous results, we believe that C1 represents a highly expressed subtype of nucleotide metabolism and immune DEGs in HCC, with more obvious immune infiltration, lower tumor purity, and poor prognosis.

### 3.2. Prognosis Prediction Model Based on Nucleotide Metabolism and Immune-Related Genes

**Figure 2:**
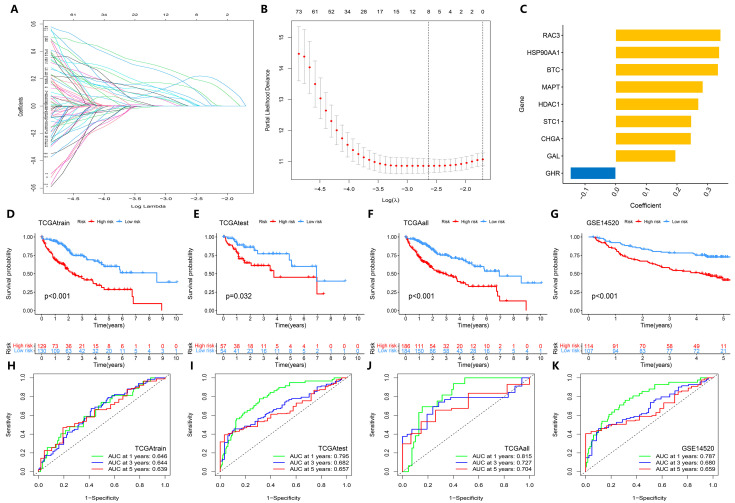
Construction and overall performance evaluation of the prognosis prediction model. (**A**) LASSO regression coefficient distribution map; one line represents one gene. (**B**) Distribution plot of the LASSO regression parameters. (**C**) Bar chart showing the model coefficients of the selected signature genes. (**D**–**G**) Kaplan–Meier curve analysis of high-risk and low-risk HCC patients in each dataset, including internal datasets TCGAtrain, TCGAtest, and TCGAall, and the external GEO test dataset GSE14520. (**H**–**K**) ROC curves at 1, 3, and 5 years in each dataset.

We combined the survival data and DEGs of TCGA-LIHC patients, and randomly divided the samples into the TCGAtrain group and TCGAtest group according to a 7:3 fraction. Through univariate Cox analysis and LASSO regression with cross-validation, nine signature genes (*RAC3*, *HSP90AA1*, *BTC*, *MAPT*, *HDAC1*, *STC1*, *CHGA*, *GAL*, *GHR*) were identified to construct the prognostic model (Figure 2A–C; Supplementary Tables S4 and S5). The high-risk and low-risk groups were divided according to the median risk score of each sample. Through the analysis of clinical characteristics by chi-squared test, we found that there were no differences in age, sex, grade, stage, T, N, and M stage between the high-risk and low-risk groups (*p* > 0.05, Supplementary Table S6), indicating that the prediction model had no statistical deviation in the clinical characteristics of the sample grouping; using the GSE14520 dataset as an external validation set and comparing the survival rates of TCGAtrain, TCGAtest, and TCGAall groups between the high-risk and low-risk HCC patients, it was found that the survival rate of the high-risk group was significantly lower than that of the low-risk group (*p* < 0.05, Figure 2D–G). ROC curves at 1, 3, and 5 years showed that the area under the curve of each group was greater than 0.5 (Figure 2H–K), indicating that the model has predictive power to discriminate the prognostic risk of HCC patients in the high-risk and low-risk groups.

### 3.3. Characterizing the Clinical Relevance of the Prediction Model

**Figure 3:**
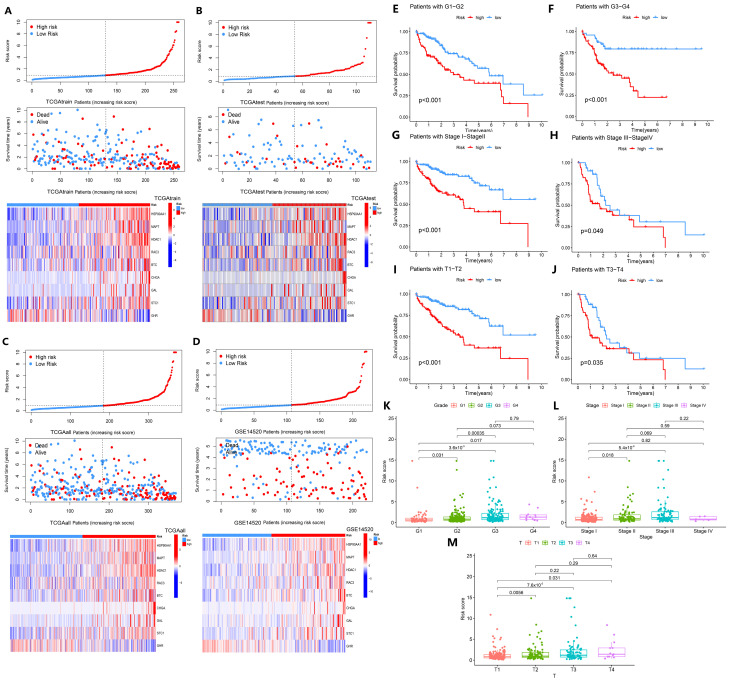
Analysis of basic clinical features associated with the high- and low-risk groups. (**A**–**D**) Risk curves, survival status, and prognostic signatures heatmap in TCGAtrain, TCGAtest, GEOtest (GSE14520), and TCGAall datasets. (**E**,**F**) Kaplan–Meier curve analysis of pairwise comparisons of G1 to G2 and G3 to G4 between high-risk and low-risk groups. (**G**,**H**) Kaplan–Meier curve analysis of pairwise comparisons of stage I to stage II and stage III to stage IV between high-risk and low-risk groups. (**I**,**J**) Kaplan–Meier curve analysis of pairwise comparisons of T1 to T2 and T3 to T4 between high-risk and low-risk groups. (**K**–**M**) Comparison of different grades, different stages, and different T stages.

The model characteristics were verified using internal and external datasets, and it was found that as the risk increased, the curve became progressively steeper and the number of patients who died gradually increased (Figure 3A–D), which was consistent with the expected prediction. In the TCGAtrain dataset, the expression of the GHR gene decreased with higher risk, while the expression of the *HSP90AA1*, *HDAC1*, *RAC3*, *STC1*, *MAPT*, and *BTC* genes increased (Figure 3A–D), but the *CHGA* and *GAL* genes were only upregulated in some very-high-risk samples. This suggests that *HSP90AA1*, *HDAC1*, *RAC3*, *STC1*, *MAPT*, *BTC*, *CHGA*, and *GAL* are high-risk genes, and *GHR* is a low-risk gene. For different clinical characteristics of the high-risk and low-risk groups, the survival rate of the high-risk group was lower in grade (G1–4), stage (I–IV), and T stage (T1–4) compared with the low-risk group (*p* < 0.05, Figure 3E–J), showing that our model is suitable for predicting HCC in different periods. Looking at the risk scores of patients among clinical characteristics, there were no significant differences in age, sex, N stage, and M stage in HCC patients (*p* > 0.05, Supplementary Figure S4), but there were significant differences in grade, stage, and T stage (*p* < 0.05, Figure 3K–M). Among them, the risk scores of the patients gradually increased during the progression from G1 to G4, stage I to stage III, and T1 to T4 (Figure 3K–M, *p* < 0.05). The result suggests that the model is able to predict differences in clinical characteristics and prognosis of HCC patients with different progression in the high-risk and low-risk groups.

### 3.4. Nomogram Derived from the Prediction Model Predicts Survival Partly Independent of the Tumor Stage

**Figure 4:**
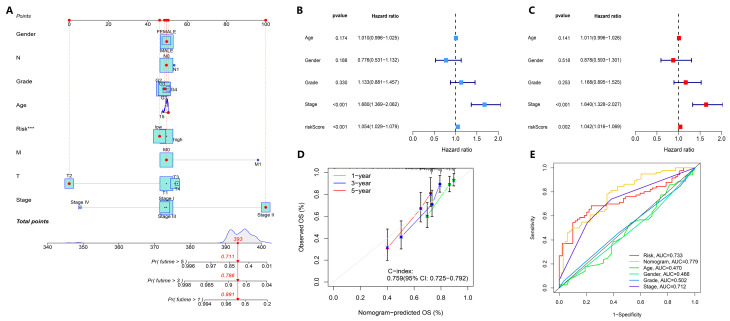
Construction and evaluation of the prediction model-derived nomogram. (**A**) Nomogram prediction of OS in TCGA-LIHC patients based on prognostic features of nucleotide metabolism and immune DEGs. For each patient, the total score was calculated by adding the scores obtained from the subscales for each variable. On the basis of the total score, the underlying scale was used to predict the probability of survival at 1, 3, or 5 years. The red line indicates the calculation process and the principle of the nomogram. (**B**,**C**) Univariate and multivariate Cox regression analysis of risk scores and various clinical characteristics. (**D**) Calibration curve between predicted and actual survival at 1, 3, and 5 years. (**E**) ROC curves of the 1-year, 3-year, and 5-year survival rates of TCGA-LIHC patients, including the nomogram. *** *p* < 0.001.

To further demonstrate the prediction ability of our model in comparison with basic clinical characteristics, logistic regression was performed on the entire TCGA-LIHC cohort and a survival prediction nomogram was constructed. As shown in the figure, the 1-year, 3-year, and 5-year survival rates predicted by the model were 0.891, 0.786, and 0.711, respectively (Figure 4A; Supplementary Table S7). The C-index value of the calibration curve was 0.759, indicating that the nomogram predicted survival and actual survival well (Figure 4D). There were more prognostic features included in the model, so we further tested whether the nomogram can be used as an independent prognostic factor. By univariate Cox regression analysis, we found that the hazard ratio (HR) for the risk score was 1.054 (95% CI: 1.029–1.079; *p* < 0.001) (Figure 4B). Multivariate Cox regression analysis showed that the HR for the risk score was 1.042 (95% CI: 1.016–1.069; *p* = 0.002) (Figure 4C). Furthermore, the stage of HCC was *p* < 0.001 in univariate and multivariate Cox regression analysis, suggesting that the stage and risk score of HCC patients can be used as independent risk factors to predict the prognosis of HCC patients. In addition, the results of the DCA decision curve further suggested that stage was closely related to the poor prognosis of HCC patients (Supplementary Figure S5). As shown in Figure 4E, the AUC of the nomogram was 0.779, indicating that the prediction of survival of HCC patients by the nucleotide metabolism and immune-related gene signatures model is accurate, indicating its potential clinical value.

### 3.5. Links Between Prognostic Model Results and Immunotherapy Efficacy

**Figure 5:**
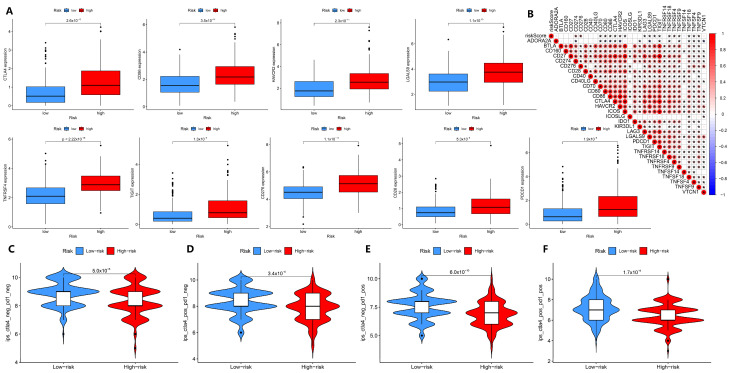
The effectiveness of the prognostic model assessing immune status and immunotherapy efficacy. (**A**) Comparison of the expression of immune checkpoint-related genes in TCGA-LIHC patients between high-risk and low-risk groups (only 9 genes were listed). (**B**) Correlation analysis of risk scores and immune checkpoint-related genes in TCGA-LIHC patients. (**C**–**F**) Comparison of the immunotherapy effects in TCGA-LIHC patients in the high-risk and low-risk groups when receiving PD-1 and CTLA-4 alone or in combination (GES10141 dataset). * *p* < 0.05.

Next, we explored the role of our model in assessing immune status and immunotherapy efficacy. The intersection of 79 immune checkpoint genes discovered by Hu et al. [20] and 47 HCC-related immune checkpoint genes discovered by Long et al. [21] was used to obtain 31 immune checkpoint genes of HCC for analysis. Through the correlation analysis of immune checkpoint-related genes, it was found that most of the immune checkpoint-related genes were positively correlated with the high-risk and low-risk groups of HCC patients, and only the *ADORA2A* gene was negatively correlated with the risk of HCC patients (Figure 5A,B, *p* < 0.05). However, there was no statistical difference in the expression of the *ADORA2A* gene in the high-risk and low-risk groups (Supplementary Figure S6, *p* > 0.05). This suggests that our model can be used to assess the association of risk differences in HCC patients with immune checkpoint genes, and our model may serve as a new indicator for the efficacy of immunotherapy. To test this idea, LIHC immunotherapy data of HCC were obtained through the TCIA database, and we compared the effect of *PD-1* and *CTLA-4* treatment between the high-risk and low-risk groups. We found a significant difference in immunotherapy response between the high-risk and low-risk groups in the GSE10141 dataset (*p* < 0.05). Compared to the high-risk group, the low-risk group fared better when treated with *CTLA4* alone, *PD-1* alone, or both in combination (Figure 5C–F). However, the response of HCC patients in the GSE14520 and GSE27150 datasets to immunotherapy was different from that in the GSE10141 dataset. Only the high-risk and low-risk groups of HCC patients in the double-negative group in the GSE14520 dataset were statistically different. There was no difference in immunotherapy efficacy between high-risk and low-risk HCC patients in the GSE27150 dataset (Supplementary Figure S7). In conclusion, our model can predict the differences in immune status and immunotherapy efficacy between high-risk and low-risk groups of HCC patients for most of the datasets investigated here.

### 3.6. Tumor Immunity-Related Biological Features Are Associated with the Prediction Results

**Figure 6:**
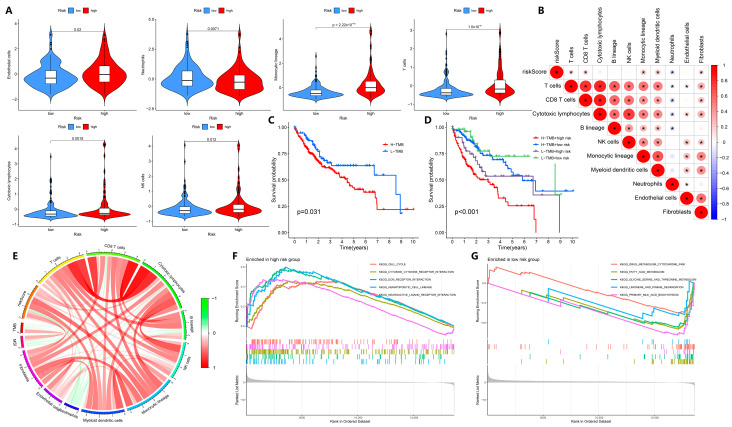
Exploration of tumor immunity-related biological factors. (**A**) Comparison of immune cells between the high- and low-risk groups of TCGA-LIHC patients. (**B**) Correlations between the relative abundance of immune microenvironment-related cell types and the risk score. (**C**,**D**) Survival rate and Kaplan–Meier curves of high-TMB and low-TMB groups, and high-risk and low-risk groups in TCGA-LIHC patients. (**E**) Correlation analysis of TMB, MSI, and immune cell infiltration in patients with TCGA-LIHC. (**F**,**G**) KEGG enrichment analysis in the high-risk and low-risk groups of TCGA-LIHC patients. * *p* < 0.05.

Similarly, our model is advantageous for assessing the association between risk differences and immune infiltration in HCC patients. Compared to the low-risk group, the high-risk group had more immune cells, such as NK cells, monocytic lineage, endothelial cells, T cells, and myeloid dendritic cells, but the low-risk group had more neutrophils (*p* < 0.05, Figure 6A; Supplementary Figure S8). Furthermore, neutrophils were negatively correlated with the risk of HCC patients, and other immune cells were positively correlated with the risk of HCC patients (*p* < 0.05, Figure 6B).

While tumor mutation burden (TMB) has been considered as a biomarker for predicting cancer progression and treatment outcome [23], physiological mismatch repair (MMR) proteins aim to detect and correct errors in mismatched nucleotides, the defects of which can lead to microsatellite instability (MSI) in DNA [24]. Therefore, we also analyzed the role of our predictive model in TMB. The TMB of HCC patients was divided into high-TMB and low-TMB groups, and we found that the survival rate of the high-TMB group was significantly lower than that of the low-TMB group (*p* < 0.05, Figure 6C; Supplementary Table S8). When the patient risk score was combined with TMB, we found that HCC patients with high TMB in the high-risk group had a lower survival rate, whereas HCC patients in the high-risk group, even those with low TMB, had a lower survival rate than patients in the low-risk group (*p* < 0.05, Figure 6D), suggesting that the risk score and TMB provide independent prognostic information. We analyzed the correlation between HCC risk score, TMB, MSI, and immune cells in HCC patients. According to Figure 6E, MSI was positively correlated with TMB and patient risk score. Except for neutrophils, HCC patient risk scores were positively correlated with most immune cells. In fact, the survival risk of HCC patients and TMB are indeed independent of each other. These results indicate that NMIRGs can be used to predict the prognostic risk of HCC patients, with the risk positively correlated with TMB and MSI, and the high risk is closely correlated with immune-related processes.

We further analyzed the functions or pathways of enrichment among the high-risk and low-risk groups by the GSEA enrichment method. As shown in Figure 6F, five biological pathways, including cell cycle, cytokine–cytokine receptor interaction, ECM receptor interaction, hematopoietic cell lineage, and neuroactive ligand–receptor interaction were enriched in the high-risk group Five biological processes, including cytochrome p450, fatty acid metabolism, glycine, serine, and threonine metabolism, limonene and degradation, and primary bile acid biosynthesis were enriched in the low-risk group (Figure 6G). This suggests that immune-related biological processes are mainly enriched in the high-risk group of HCC patients, whereas metabolism-related processes are mainly enriched in the low-risk group of HCC patients.

### 3.7. Comparison with Previous HCC Prognostic Models

**Figure 7:**
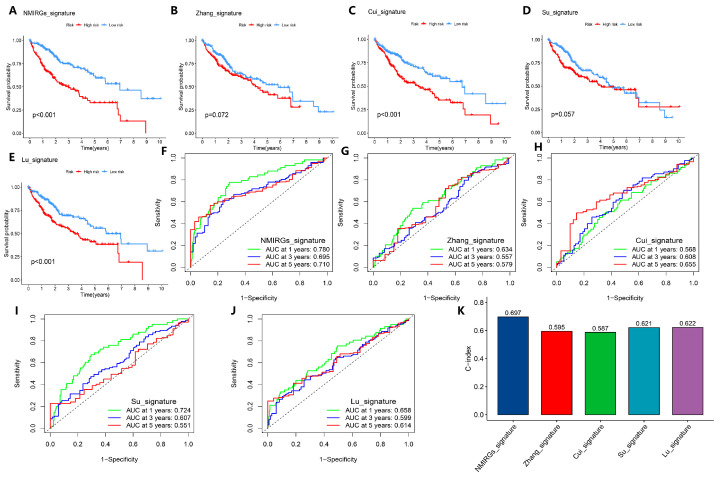
Comparison of model predictive performance with the previous prognostic signatures. (**A**–**E**) Kaplan–Meier curve analysis of all prediction models, including the prediction models published in the references. (**A**–**E**) are NMIRGs_signature (our model), Zhang_signature, Cui_signature, Su_signature, and Lu_signature, respectively. (**F**–**J**) ROC curves of the 1-year, 3-year, and 5-year survival in all prediction models. (**F**–**J**) are NMIRGs_signature (our model), Zhang_signature, Cui_signature, Su_signature, and Lu_signature, respectively. (**K**) C-index analysis of all prediction models.

Finally, we compared the prediction score of the external validation set GSE14520 with the models already reported in the references, including Zhang_signature [25] (chemokines and chemokine receptors-related three-gene model), Cui_signature [26] (immune index-related ten-gene model), Su_signature [27] (immunogenic cell death-related three-gene model), and Lu_signature [28] (inflammatory response- and immunity status-related seven-gene model). Using the same calculation method, HCC samples were divided into high-risk and low-risk groups, and the survival analysis showed that our model is better than Zhang_signature and Su_signature, and is at least comparable to Cui_signature and Lu_signature (Figure 7A–E). Furthermore, the ROC curve analysis showed that our NMIRGs_signature indeed showed better performance, where the areas under the ROC curve for 1-year, 3-year, and 5-year survival rates in our model were 0.780, 0.695, and 0.710, respectively (Figure 7F–J). In comparison with other models, the C-index of our model was 0.697, which is also higher than Zhang_signature (C-index = 0.595), Cui_signature (C-index = 0.587), Su_signature (C-index = 0.621), and Lu_signature (C-index = 0.622) (Figure 7K). These results indicate that our model has a high accuracy in predicting the prognosis of HCC patients.

### 3.8. Experimental Validation of Signature Genes via qPCR and HPA Database

**Figure 8:**
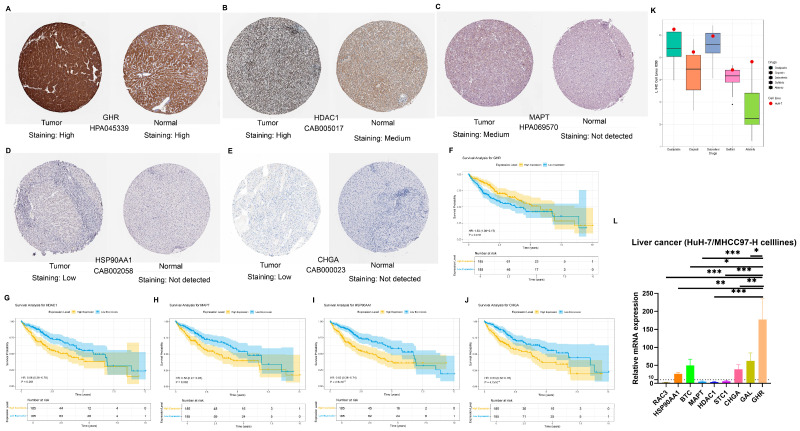
NMRIG signature genes are differentially expressed in liver cancer. (**A**–**E**) The protein expression levels of GHR, HDAC1, MAPT, HSP90AA1, and CHGA in HCC patients and normal tissues by immunohistochemistry (IHC) obtained from the HPA online database. (**F**–**J**) Survival analysis of these genes from the TCGA database. (**K**) IC50 comparison of the relative sensitivity of HuH7 cell lines with other HCC cell lines to the HCC therapeutics Oxaliplatin, Cisplatin, Dabrafenib, Gefitinib, and Afatinib obtained from GDSC. (**L**) The gene expression level of NMRIG signatures in Huh7 cell lines relative to MHCC97H cell lines was detected by RT-qPCR. * *p* < 0.05, ** *p* < 0.01, *** *p* < 0.001.

The expression of these nine NMIRG signature genes used in the construction of the model was searched in the HPA online database. We found that the protein levels of eight out of nine signature genes (including *CHGA*, *GAL*, *GHR*, *RAC3*, *HSP90AA1*, *MAPT*, *HDAC1*, and *STC1*; Supplementary Table S9, Figure 8A–E) had been measured in HCC tissues. Among them, the proteins of GHR and HDAC1 showed strong positivity in HCC tissues, the protein of MAPT was moderately positive in HCC tissues, the proteins of STC1, HSP90AA1, and CHGA showed low positivity in HCC tissues, while RAC3 and GAL did not show expression in HCC tissues (Supplementary Figure S9A–C). No immunohistochemical data on liver cancer were available for BTC protein. Moreover, consistent with the expression of NMIRG proteins retrieved from the HPA database in HCC tissues, the *GHR*, *HDAC1*, *MAPT*, *HSP90AA1*, and *CHGA* genes are significantly associated with the prognosis of HCC patients (*p* < 0.05, Figure 8F–J, Supplementary Figure S9D–F). Among them, HCC patients with low expression of *HDAC1*, *MAPT*, *HSP90AA1*, and *CHGA* have a better prognosis, while those with low expression of *GHR* show the opposite, having a worse prognosis (Figure 8F–J). Although there could be some false-negative results among the immunohistochemical data, the prognostic associations of NMIRG signature genes can be at least partly confirmed at the protein level. We analyzed the IC50 values of common liver cancer treatment drugs (Oxaliplatin, Cisplatin, Dabrafenib, Gefitinib, and Afatinib) for liver cancer cell lines in the GDSC database. We found that compared with other liver cancer cell lines, the HuH-7 cell line had higher IC50 values for these five liver cancer treatment drugs, indicating that the HuH-7 cell line has stronger drug resistance (Figure 8K, Supplementary Table S10). Through RT-qPCR detection, the relative expression levels of the NMIRG signature gene mRNA were expressed to different degrees in HuH-7 cell lines, among which the relative expression level of the GHR gene was the highest. Dysregulation of signature genes (e.g., *GHR*, *BTC*) in Huh-7 cells (*p* < 0.05, Figure 8L, Supplementary Table S11), while HPA IHC revealed prognostic protein expression patterns (e.g., low *HDAC1/MAPT* linked to better survival, Figure 8F–J), supporting our bioinformatic predictions.

## 4. Discussion

HCC remains a challenge to global health. Although immunotherapy has made a breakthrough, the treatment outcomes are not uniform due to differences in tumor microenvironment, and there is still a significant proportion of HCC patients with poor prognosis [13]. Therefore, it is urgent to investigate effective prognostic indicators. Given the role of nucleotide metabolism in HCC progression and the immune microenvironment [4,6,7,10], the combination of both predictors has greater predictive value [29]. Therefore, this study integrates nucleotide metabolism and immune-related differential genes and HCC patients’ clinical features, and establishes a prognosis prediction model for HCC patients, in order to help clinicians more accurately evaluate the prognosis of HCC patients, provide personalized treatment, and identify new prognostic markers.

We first classified HCC patients into C1 and C2 subtypes by NMF clustering and found that the C1 cluster had high expression of nucleotide metabolism and immune-related genes, low tumor purity, more immune cell infiltration, and significantly lower overall survival (OS) and progression-free survival compared with C2. This indicates that the C1 subtype represents HCC patients with a poorer prognosis, and it also suggests that the high expression of NMIRGs is associated with worse prognosis and greater immune infiltration in HCC patients. This suggests that NMIRGs have predictive potential. Therefore, next, we screened out nine nucleotide metabolism and immune signature genes to construct a prognostic model of HCC. *HSP90AA1*, *HDAC1*, *RAC3*, *STC1*, *MAPT*, *BTC*, *CHGA*, and *GAL* were identified as high-risk genes, and *GHR* as a low-risk gene. Receptor-associated coactivator 3 (*RAC3*) is an oncogene that is highly expressed in many malignant tumors and regulates cell cycle progression and cell proliferation, invasion, and migration [30]. In fact, our study found a complex expression pattern of *RAC3* in HCC. Our findings align with emerging evidence suggesting *RAC3* acts as a context-dependent oncogene in specific HCC molecular subgroups (e.g., SOX6-high), while its function may be compensated or inactivated in others (including subtypes represented by HuH-7 or the analyzed TCGA cohort) [31]. Betacellulin (BTC) proteins are ligands for the *EGFR/ERBB4* ligand [32], with established literature showing that *BTC* overexpression by HCC cells and *EGFR* expression by tumor-associated endothelial cells collaboratively enhance tumor vascularity via paracrine signaling [33]. Intriguingly, while the HPA database currently lacks immunohistochemical data for BTC in liver cancer, survival analysis of TCGA-LIHC data revealed no significant survival difference based on *BTC* expression levels. However, our RT-qPCR analysis demonstrated relatively high *BTC* mRNA expression in the HuH7 cell line, suggesting a spatially restricted role for *BTC* within the tumor microenvironment (TME). Specifically, *BTC*’s pro-tumorigenic function may be critically dependent on localized expression and signaling at the interface between tumor cells and adjacent endothelial cells. Chromogranin A (*CHGA*) may play a role in early tumor progression [34]. It is mentioned in most liver cancer prediction models, and we found that it is indeed differentially expressed in HCC patients and associated with prognosis. These findings, alongside established knowledge of *CHGA* as a neuroendocrine marker [35], strongly support *CHGA* as a promising biomarker for HCC, warranting further investigation into its functional role and precise prognostic utility. Human heat shock protein 90 alpha family class A member 1 (*HSP90AA1*) [36], microtubule-associated protein tau (*MAPT*) [37], and histone deacetylase 1 (*HDAC1*) [38] are associated with poor prognosis in HCC. Knockdown of growth hormone receptor (*GHR*) increases the sensitivity of HCC cells to sorafenib [39]. Critically, our signature’s clinical applicability is reinforced by experimental validation: mRNA levels of key genes (e.g., *GHR*, *BTC*) were confirmed via RT-qPCR in HCC cell lines, while HPA-derived IHC data revealed prognostic protein expression patterns (e.g., HDAC1, MAPT). While further mechanistic studies are warranted, this multi-level validation underscores the biological relevance of our model. In addition, the AUC of the nomogram reached 0.803, indicating that our model has good predictive performance, and these genes are expected to become potential targets in HCC. According to the expression of NMIRG signature genes, personalized guidance for HCC patients can be provided to reduce the disease burden.

We also evaluated the predictive performance of the model by examining immune infiltration and the effect of immunotherapy. Previous studies have shown that tumor cells evade immune killing by altering the local immune microenvironment [40]. Therefore, changes in the immune microenvironment are critical for assessing tumor progression. We found that our model could well evaluate the correlation between prognostic risk differences in HCC patients and immune checkpoint genes and immune infiltration, and the changes in neutrophils were also consistent with the negative correlation between the number of tumor-infiltrating neutrophils and the number of T cells reported in reference [41]. In addition, when evaluating the effect of *PD-1* and *CTLA-4* immunotherapy in high-risk and low-risk HCC patients, the results of the external validation dataset were also in line with expectations. There are differences in the effectiveness of immunotherapy in different HCC patients, so the efficacy of immunotherapy is not the same in different datasets. For example, the GSE10141 dataset included more HCC patients with high alcohol consumption than the GSE14520 dataset, and our model showed that they had better immunotherapy outcomes for *PD-1* and *CTLA-4* in the GSE10141 dataset.

In order to explore the potential regulatory mechanism of NMRIGs signature in HCC, we performed GSEA enrichment analysis in the high-risk and low-risk groups and found that the enrichment of immune-related processes was mainly in the high-risk group of HCC patients, while the enrichment of metabolism-related processes was mainly in the low-risk group. This finding aligns with previous studies suggesting that preserved metabolic function is often associated with better prognosis in HCC [42,43]. This is an interesting result, which shows that the analysis of HCC progression cannot only consider a single feature, and the combined analysis of multiple features can enable a more comprehensive interpretation of HCC prognosis. TMB and MSI, as indicators of somatic mutation [24], have been used as biomarkers to predict cancer progression and treatment outcomes. High TMB indicates increased production of neoantigens, which can affect the efficacy of immunotherapy. Our analysis showed that the risk score of HCC patients was positively correlated not only with MSI and TMB, but also with most immune cell abundances (except neutrophils). Nonetheless, the NMIRG risk score and TMB are largely independent of each other in predicting HCC prognosis.

In summary, we have constructed a clinical model by nine nucleotide metabolism and immune-related signature genes that predicts HCC prognosis, which has advantages in predicting the risk and immune microenvironment of HCC patients. It is expected that this model will help clinicians to more accurately assess the prognosis of HCC patients and provide personalized treatment options. In addition, the differential expression of NMIRG signature genes suggests their potential as biomarkers, providing a new perspective for the diagnosis and treatment of HCC. Most importantly, our predictive model has a higher accuracy in predicting the prognosis of HCC patients than some single-feature predictive models.

## 5. Conclusions

In conclusion, hepatocellular carcinoma continues to pose significant therapeutic challenges due to its heterogeneity and poor prognosis, underscoring the need for advanced predictive tools. This study successfully developed and validated a novel prognostic signature by integrating nucleotide metabolism and immune-related genes (NMIRGs) for HCC. We identified two molecular subtypes (C1/C2) with distinct prognoses and immune profiles, and constructed a robust nine-gene NMIRG signature that effectively stratifies HCC patients into high- and low-risk groups. This signature serves as an independent prognostic factor and outperforms existing models. Crucially, it correlates with altered immune cell infiltration, elevated immune checkpoint expression, increased tumor mutation burden (TMB), and microsatellite instability (MSI), suggesting a differential response to immunotherapy. The integration of bioinformatic modeling with experimental mRNA validation and protein-level evidence (HPA) provides a robust framework for HCC risk stratification. The NMIRG signature provides a valuable tool for refined prognosis and offers insights into the HCC immune microenvironment. The identified genes represent promising biomarker/therapeutic targets. Future research should prioritize prospective validation in diverse cohorts, assess its utility for guiding personalized therapy (especially immunotherapy), and explore the mechanistic roles of these NMIRGs in HCC pathogenesis and treatment resistance.

## Data Availability

The original contributions presented in the study are included in the article/Supplementary Materials; further inquiries can be directed to the corresponding authors.

## Supplementary Materials

The following supporting information can be downloaded at https://www.mdpi.com/article/10.3390/biology14081079/s1: Supplementary Figure S1. NMF clustering of HCC based on genes related to nucleotide metabolism and immunity. Supplementary Figure S2. The heatmap of nucleotide metabolism and immune-related DEGs. Supplementary Figure S3. Results of the nsNMF decomposition level. Supplementary Figure S4. Comparison of different genders, different ages, different N stages, and M stages. Supplementary Figure S5. DCA decision curve of each clinical feature. Supplementary Figure S6. Comparison of immune checkpoint-related genes in the high- and low-risk groups of TCGA-LIHC patients (excluding all other immune checkpoints listed in the article). Supplementary Figure S7. Comparison of the immunotherapy effects in TCGA-LIHC patients in the high-risk and low-risk groups (GES14520 dataset and GSE27150 dataset). Supplementary Figure S8. Comparison of immune microenvironment-related cells (B lineage, myeloid dendritic cells, CD8 T cells, fibroblasts) between high-risk and low-risk groups in patients with HCC. Supplementary Figure S9. The survival analysis of NMIRG signature genes (RAC3, GAL, STC1) and protein expression of these in HCC. Supplementary Table S1. The sequence of primers. Supplementary Table S2. The list of merged differentially expressed genes. Supplementary Table S3. The two HCC subtypes. Supplementary Table S4. Results of multivariate Cox regression analysis of HCC samples from the TCGA database. Supplementary Table S5. Results of LASSO regression analysis of HCC samples from the TCGA database. Supplementary Table S6. Chi-squared test results of clinical features of LIHC patients in the TCGA database. Supplementary Table S7. High- and low-risk nomogram results of HCC patients. Supplementary Table S8. Tumor mutation burden (TMB) results of HCC patients. Supplementary Table S9. Immunohistochemical patient information from the HPA database. Supplementary Table S10. IC50 values of liver cancer cell lines relative to drug sensitivity in the GDSC database. Supplementary Table S11. Relative expression levels of NMIRG genes.

## Abbreviations

HCC	hepatocellular carcinoma
LIHC	liver hepatocellular carcinoma
NMIRGs	nucleotide metabolism and immune-related genes
C1	cluster 1
DEGs	differentially expressed genes
TMB	tumor mutation burden
MSI	microsatellite instability
TCGA	The Cancer Genome Atlas
GEO	Gene Expression Omnibus
DAVID	Database for Annotation, Visualization, and Integrated Discovery
LASSO	Least Absolute Shrinkage and Selection Operator
MCPcounter	Microenvironment Cell Populations counter
ESTIMATE	Estimation of STromal and Immune cells in MAlignant Tumor tissues using Expression data
GSEA	Gene Set Enrichment Analysis
NMF	Non-Negative Matrix Factorization
PFS	progression-free survival
DCA	Decision Curve Analysis
ROC	Receiver Operating Characteristic
RAC3	receptor-associated coactivator 3
HSP90AA1	heat shock protein 90 alpha family class A member 1
BTC	betacellulin
MAPT	microtubule-associated protein tau
HDAC1	histone deacetylase 1
STC1	stanniocalcin-1
CHGA	chromogranin A
GAL	galanin and GMAP prepropeptide
GHR	growth hormone receptor
PD-1	programmed cell death protein 1
CTLA-4	cytotoxic T-lymphocyte-associated protein-4
ADORA2A	adenosine receptor A2a

## References

[1] Vogel A., Meyer T., Sapisochin G., Salem R., Saborowski A. (2022). Hepatocellular carcinoma. Lancet.

[2] Anwanwan D., Singh S.K., Singh S., Saikam V., Singh R. (2020). Challenges in liver cancer and possible treatment approaches. Biochim. Biophys. Acta (BBA)-Rev. Cancer.

[3] Childs A., Zakeri N., Ma Y.T., O’rOurke J., Ross P., Hashem E., Hubner R.A., Hockenhull K., Iwuji C., Khan S. (2021). Biopsy for advanced hepatocellular carcinoma: Results of a multicentre UK audit. Br. J. Cancer.

[4] Ma J., Zhong M., Xiong Y., Gao Z., Wu Z., Liu Y., Hong X. (2021). Emerging roles of nucleotide metabolism in cancer development: Progress and prospect. Aging.

[5] Wu H.L., Gong Y., Ji P., Xie Y.F., Jiang Y.Z., Liu G.Y. (2022). Targeting nucleotide metabolism: A promising approach to enhance cancer immunotherapy. J. Hematol. Oncol..

[6] Jain S., Jacobson K.A. (2021). Purinergic Signaling in Liver Pathophysiology. Front. Endocrinol..

[7] Mullen N.J., Singh P.K. (2023). Nucleotide metabolism: A pan-cancer metabolic dependency. Nat. Rev. Cancer.

[8] Xu D., Tian Y., Xia Q., Ke B. (2021). The cGAS-STING Pathway: Novel Perspectives in Liver Diseases. Front. Immunol..

[9] Donne R., Lujambio A. (2023). The liver cancer immune microenvironment: Therapeutic implications for hepatocellular carcinoma. Hepatology.

[10] Han T., Kang D., Ji D., Wang X., Zhan W., Fu M., Xin H.-B., Wang J.-B. (2013). How does cancer cell metabolism affect tumor migration and invasion?. Cell Adhes. Migr..

[11] Shen B., Zhang G., Liu Y., Wang J., Jiang J. (2022). Identification and Analysis of Immune-Related Gene Signature in Hepatocellular Carcinoma. Genes.

[12] Chen Y., Shen C., Wu J., Yan X., Huang Q. (2023). Role of immune related genes in predicting prognosis and immune response in patients with hepatocellular carcinoma. J. Biochem. Mol. Toxicol..

[13] Xu Y.J., He M.K., Liu S., Huang L.C., Bu X.Y., Kan A., Shi M. (2021). Construction of a single nucleotide variant score-related gene-based prognostic model in hepatocellular carcinoma: Analysis of multi-independent databases and validation in vitro. Cancer Cell Int..

[14] Wei T., Liu J., Ma S., Wang M., Yuan Q., Huang A., Wu Z., Shang D., Yin P. (2023). A Nucleotide Metabolism-Related Gene Signature for Risk Stratification and Prognosis Prediction in Hepatocellular Carcinoma Based on an Integrated Transcriptomics and Metabolomics Approach. Metabolites.

[15] Sherman B.T., Hao M., Qiu J., Jiao X., Baseler M.W., Lane H.C., Imamichi T., Chang W. (2022). DAVID: A web server for functional enrichment analysis and functional annotation of gene lists (2021 update). Nucleic Acids Res..

[16] Bhattacharya S., Dunn P., Thomas C.G., Smith B., Schaefer H., Chen J., Hu Z., Zalocusky K.A., Shankar R.D., Shen-Orr S.S. (2018). ImmPort, toward repurposing of open access immunological assay data for translational and clinical research. Sci. Data.

[17] Yoshihara K., Shahmoradgoli M., Martínez E., Vegesna R., Kim H., Torres-Garcia W., Treviño V., Shen H., Laird P.W., Levine D.A. (2013). Inferring tumour purity and stromal and immune cell admixture from expression data. Nat. Commun..

[18] Becht E., Giraldo N.A., Lacroix L., Buttard B., Elarouci N., Petitprez F., Selves J., Laurent-Puig P., Sautes-Fridman C., Fridman W.H. (2016). Estimating the population abundance of tissue-infiltrating immune and stromal cell populations using gene expression. Genome Biol..

[19] Moons K.G.M., Altman D.G., Reitsma J.B., Ioannidis J.P.A., Macaskill P., Steyerberg E.W., Vickers A.J., Ransohoff D.F., Collins G.S. (2015). Transparent Reporting of a multivariable prediction model for Individual Prognosis Or Diagnosis (TRIPOD): Explanation and Elaboration. Ann. Intern. Med..

[20] Hu F.F., Liu C.J., Liu L.L., Zhang Q., Guo A.Y. (2021). Expression profile of immune checkpoint genes and their roles in predicting immunotherapy response. Brief. Bioinform..

[21] Long M., Zhou Z., Wei X., Lin Q., Qiu M., Zhou Y., Chen P., Jiang Y., Wen Q., Liu Y. (2022). A novel risk score based on immune-related genes for hepatocellular carcinoma as a reliable prognostic biomarker and correlated with immune infiltration. Front. Immunol..

[22] Charoentong P., Finotello F., Angelova M., Mayer C., Efremova M., Rieder D., Hackl H., Trajanoski Z. (2017). Pan-cancer Immunogenomic Analyses Reveal Genotype-Immunophenotype Relationships and Predictors of Response to Checkpoint Blockade. Cell Rep..

[23] Choucair K., Morand S., Stanbery L., Edelman G., Dworkin L., Nemunaitis J. (2020). TMB: A promising immune-response biomarker, and potential spearhead in advancing targeted therapy trials. Cancer Gene Ther..

[24] Gabbia D., De Martin S. (2023). Tumor Mutational Burden for Predicting Prognosis and Therapy Outcome of Hepatocellular Carcinoma. Int. J. Mol. Sci..

[25] Zhang Z., Mao M., Wang F., Zhang Y., Shi J., Chang L., Wu X., Zhang Z., Xu P., Lu S. (2023). Comprehensive analysis and immune landscape of chemokines- and chemokine receptors-based signature in hepatocellular carcinoma. Front. Immunol..

[26] Cui X., Han L., Cui L., Fu G., Liu E., Wang D., Song B., Zhang Y., Zhou W., Wang H. (2022). Immune index: A gene and cell prognostic signature for immunotherapy response prediction in hepatocellular carcinoma. Pharmacol. Res..

[27] Su D., Zhang Z., Xia F., Liang Q., Liu Y., Liu W., Xu Z. (2023). ICD-related risk model predicts the prognosis and immunotherapy response of patients with liver cancer. Front. Pharmacol..

[28] Lu G., Du R., Feng B., Wang J., Zhang F., Pei J., Wang Y., Shang Y. (2022). A Novel Gene Signature Associated with Inflammatory Responses and Immune Status Assists in Prognosis and Intervention for Patients with HCC. J. Inflamm. Res..

[29] Xu Y.Y., Qin Z., Gao J.M., Yang Y., Lu Y., Yu F., Lv Y., Sun Z., Zhang J., Tang J. (2023). Combination of Neutrophil Count and Gensini Score as a Prognostic Marker in Patients with ACS and Uncontrolled T2DM Undergoing PCI. Cardiovasc. Innov. Appl..

[30] De Curtis I. (2019). The Rac3 GTPase in Neuronal Development, Neurodevelopmental Disorders, and Cancer. Cells.

[31] Wu D., Liu Z., Sun Y., Gou C., Shang R., Lu M., Zhang R., Wei H., Li C., Shi Y. (2025). Integrated Analysis of the Anoikis-Related Signature Identifies Rac Family Small GTPase 3 as a Novel Tumor-Promoter Gene in Hepatocellular Carcinoma. Medcomm.

[32] Dahlhoff M., Wolf E., Schneider M.R. (2014). The ABC of BTC: Structural properties and biological roles of betacellulin. Semin. Cell Dev. Biol..

[33] Chava S., Bugide S., Zhang X., Gupta R., Wajapeyee N. (2022). Betacellulin promotes tumor development and EGFR mutant lung cancer growth by stimulating the EGFR pathway and suppressing apoptosis. iScience.

[34] Mahata S.K., Corti A. (2019). Chromogranin A and its fragments in cardiovascular, immunometabolic, and cancer regulation. Ann. N. Y. Acad. Sci..

[35] Malaguarnera M., Vacante M., Fichera R., Cappellani A., Cristaldi E., Motta M. (2010). Chromogranin A (CgA) serum level as a marker of progression in hepatocellular carcinoma (HCC) of elderly patients. Arch. Gerontol. Geriatr..

[36] Zuehlke A.D., Beebe K., Neckers L., Prince T. (2015). Regulation and function of the human HSP90AA1 gene. Gene.

[37] Chang C.-W., Shao E., Mucke L. (2021). Tau: Enabler of diverse brain disorders and target of rapidly evolving therapeutic strategies. Science.

[38] Cai D., Yuan X., Cai D.Q., Li A., Yang S., Yang W., Duan J., Zhou W., Min J., Peng L. (2023). Integrative analysis of lactylation-related genes and establishment of a novel prognostic signature for hepatocellular carcinoma. J. Cancer Res. Clin. Oncol..

[39] Gao S., Ni Q., Wu X., Cao T. (2020). GHR knockdown enhances the sensitivity of HCC cells to sorafenib. Aging.

[40] Giannini E.G., Aglitti A., Borzio M., Gambato M., Guarino M., Iavarone M., Lai Q., Levi Sandri G.B., Melandro F., Morisco F. (2019). Overview of Immune Checkpoint Inhibitors Therapy for Hepatocellular Carcinoma, and The ITA.LI.CA Cohort Derived Estimate of Amenability Rate to Immune Checkpoint Inhibitors in Clinical Practice. Cancers.

[41] He G., Zhang H., Zhou J., Wang B., Chen Y., Kong Y., Xie X., Wang X., Fei R., Wei L. (2015). Peritumoural neutrophils negatively regulate adaptive immunity via the PD-L1/PD-1 signalling pathway in hepatocellular carcinoma. J. Exp. Clin. Cancer Res..

[42] Fu J., Cao Z., Zhang J., Chen Q., Wang Y., Wang S., Fang X., Xu X. (2022). Identification of two immune-related risk score signatures through integrated analysis of multi-omics data in hepatocellular carcinoma. Gene.

[43] Lu J., Shi L., Zhang C., Zhang F., Cai M. (2025). Prognostic significance of pyrimidine metabolism-related genes as risk biomarkers in hepatocellular carcinoma. J. Chemother..

