# Supplementary material for: Nucleotide Metabolism and Immune Genes Can Predict the Prognostic Risk of Hepatocellular Carcinoma and the Immune Microenvironment
Source: Biology (Basel). 2025 Aug 18;14(8):1079. doi: 10.3390/biology14081079 (PMC12383802; doi:10.3390/biology14081079)
Supplement: Supplementary file 1 [file biology-14-01079-s001.zip › biology-3668842-supplementary.pdf]

Supplementary Figures

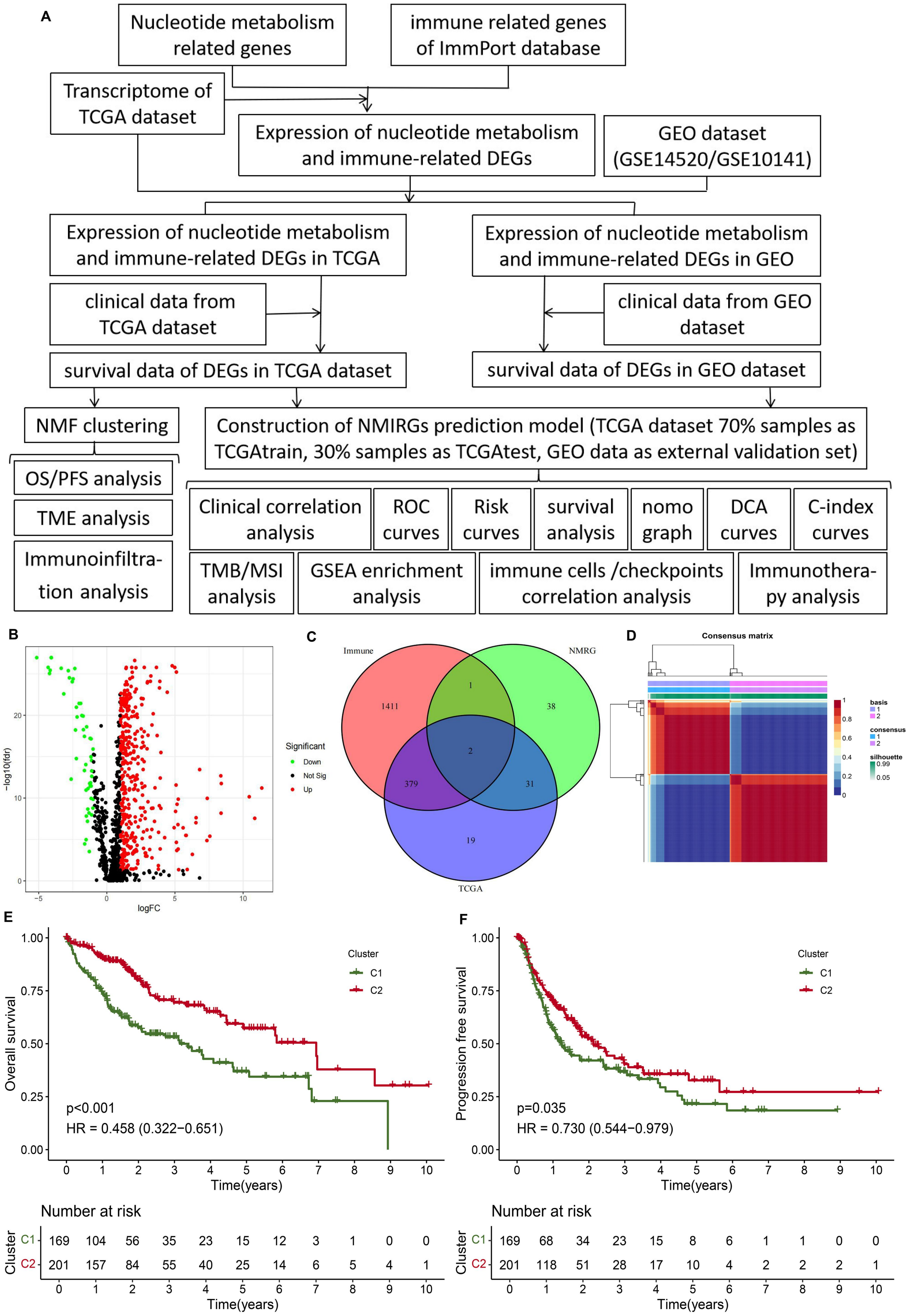

Supplementary Figure S1

(A) Flowchart of the study. **(B)** The volcano plot of DEGs, up-regulated DEGs in red and down-regulated DEGs in green. **(C)** Venn diagram of 1793 immune related genes, 72 nucleotide metabolism related genes and 431 differentially expressed genes in the TCGA database. **(D)** The heatmap of the nsNMF consensus matrix at K=2. **(E-F)** Kaplan-Meier curves for overall survival (OS) and progression-free survival (PFS) of different HCC subtypes.

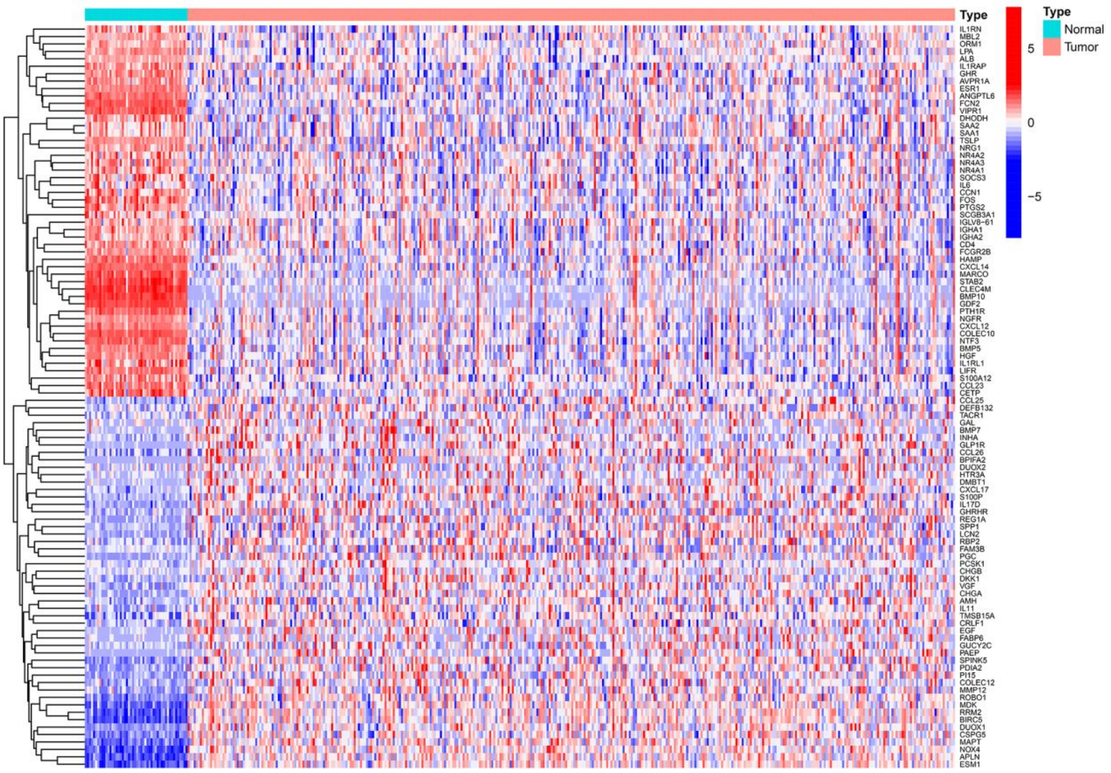

Supplementary Figure S2

The top 100 DEGs with the most significant differences of nucleotide metabolism and immune-related genes between the normal group and HCC group in LIHC samples.

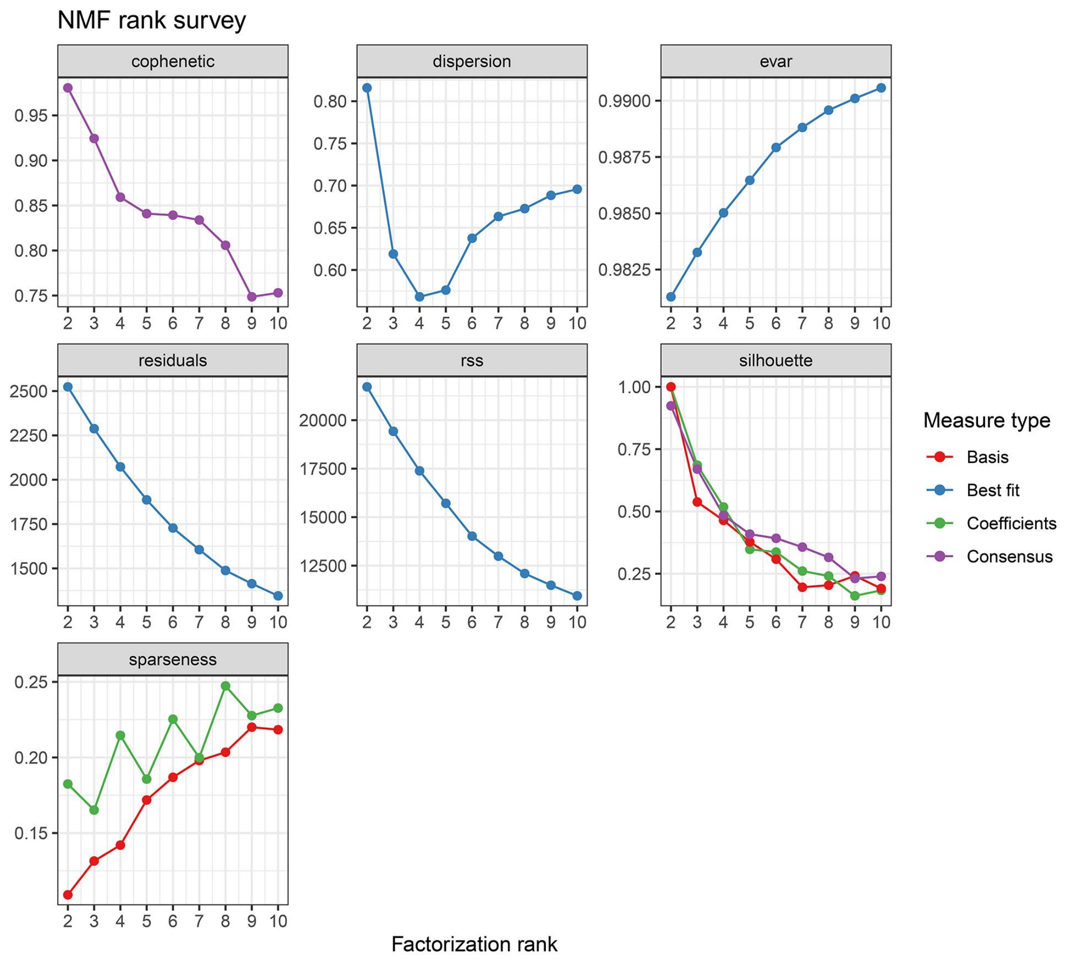

Supplementary Figure S3

Results ofthe nsNMF decomposition ranks. According to the cophenetic diagram, the first point ofthe fastest decline is regarded as the optimal rank, namely k=2.

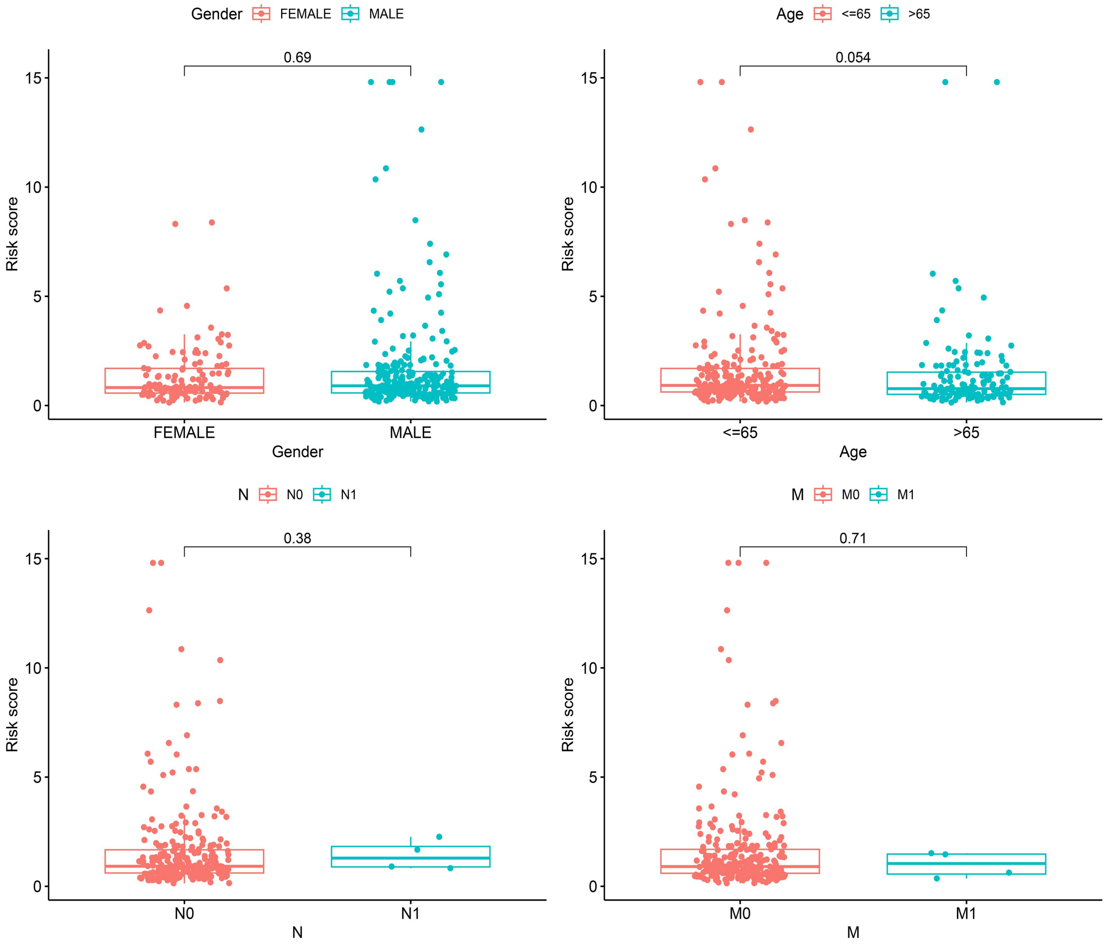

Supplementary Figure S4

(A) Comparison of different age. **(B)** Comparison of different gender. **(C)** Comparison of different N stage. **(D)** Comparison of M stage.

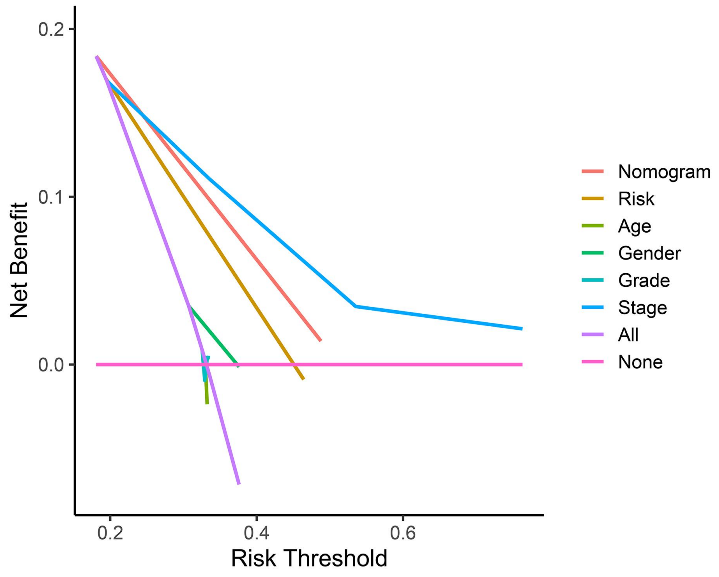

Supplementary Figure S5

The DCA decision curve of each clinical feature. The abscissa is the risk threshold, the ordinate is the net benefit, the fully positive hypothesis curve is all, the fully negative hypothesis curve is none, and the OS accuracy is measured by the area under the curve.

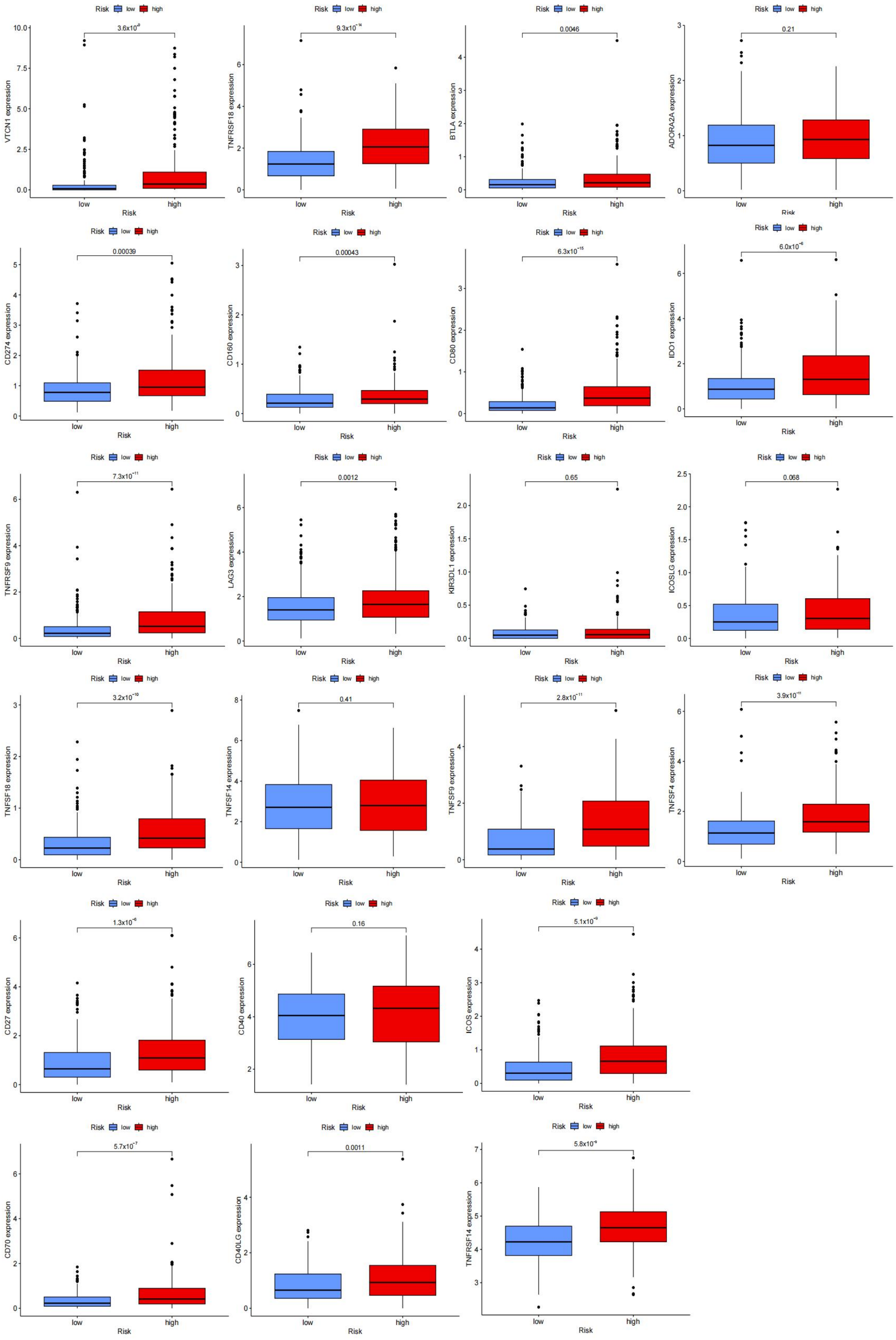

Supplementary Figure S6

Comparison of immune checkpoint-related genes in the high and low-risk groups of TCGA-LIHC patients. There are a total of 31 immune checkpoint genes, 9 ofwhich are listed in Figure 6A and the remaining 22 genes are shown here.

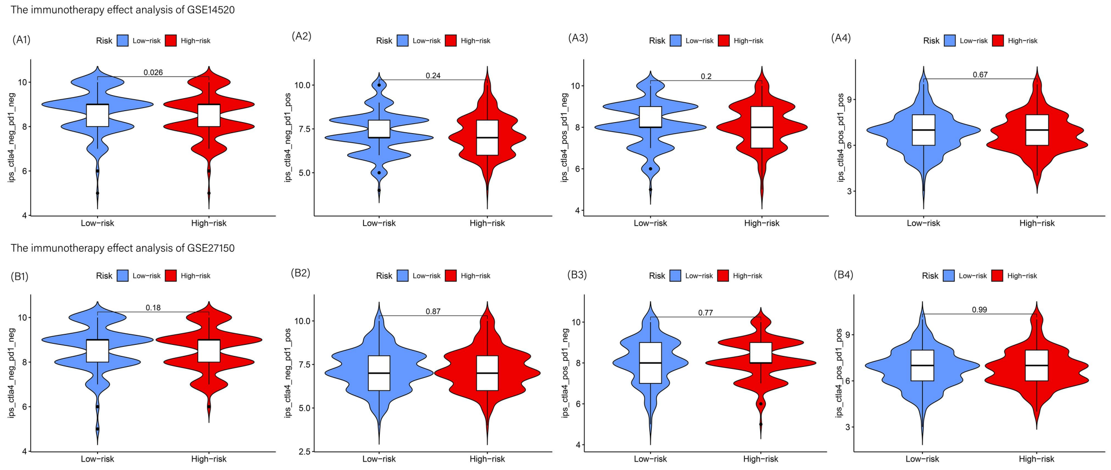

Supplementary Figure S7

Comparison of the immunotherapy effects in HCC patients in the high-risk and low-risk groups (GES10520 dataset and GSE27150 dataset). **(A)** The effects of without *PD-1* and *CTLA-4*. **(B)** The effects of treated with *PD-1* alone. **(C)** The effects of treated with *CTLA4* alone. **(D)** The effects of *PD-1* and *CTLA-4* combination.

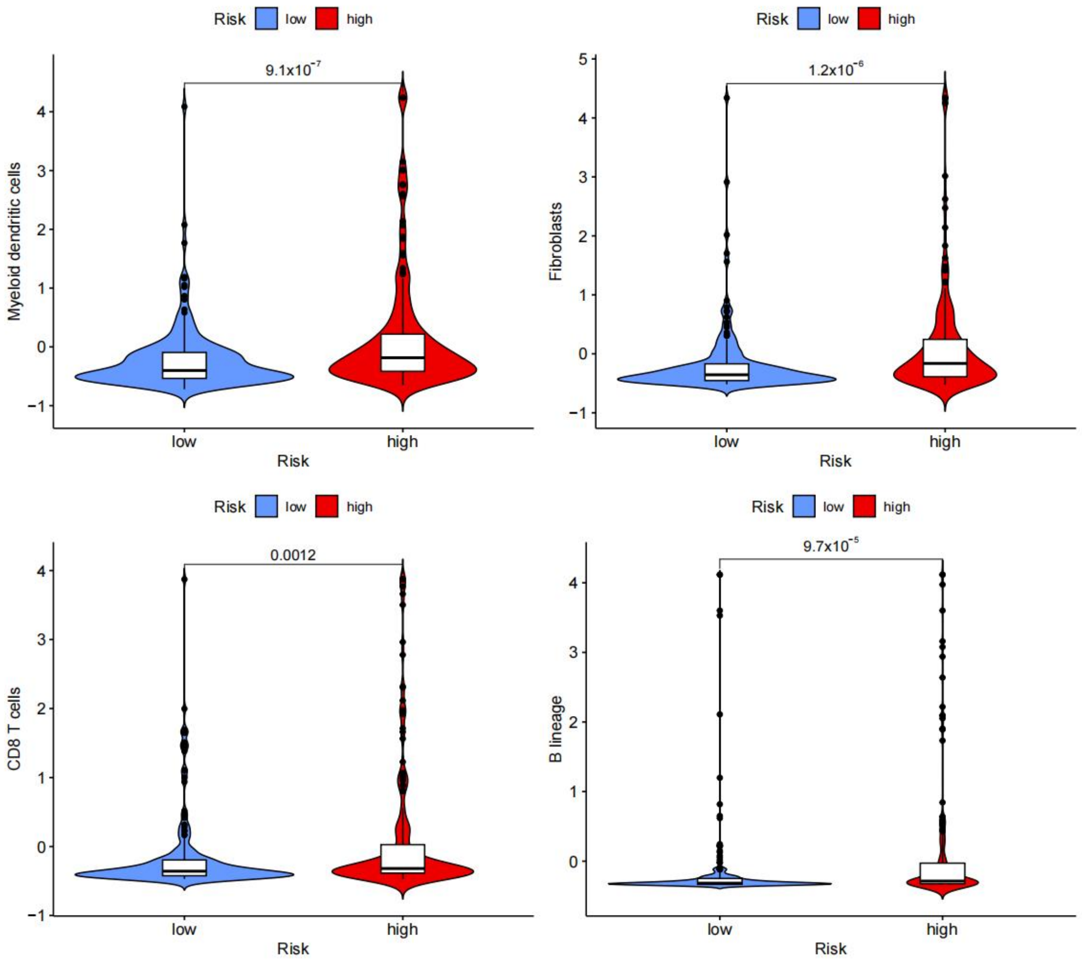

Supplementary Figure S8

Comparison of immune microenvironment-related cells between high-risk and low-risk groups in HCC patients. **(A)** Myeloid dendritic cells. **(B)** Fibroblasts. **(C)** CD8 T cells. **(D)** B lineage.

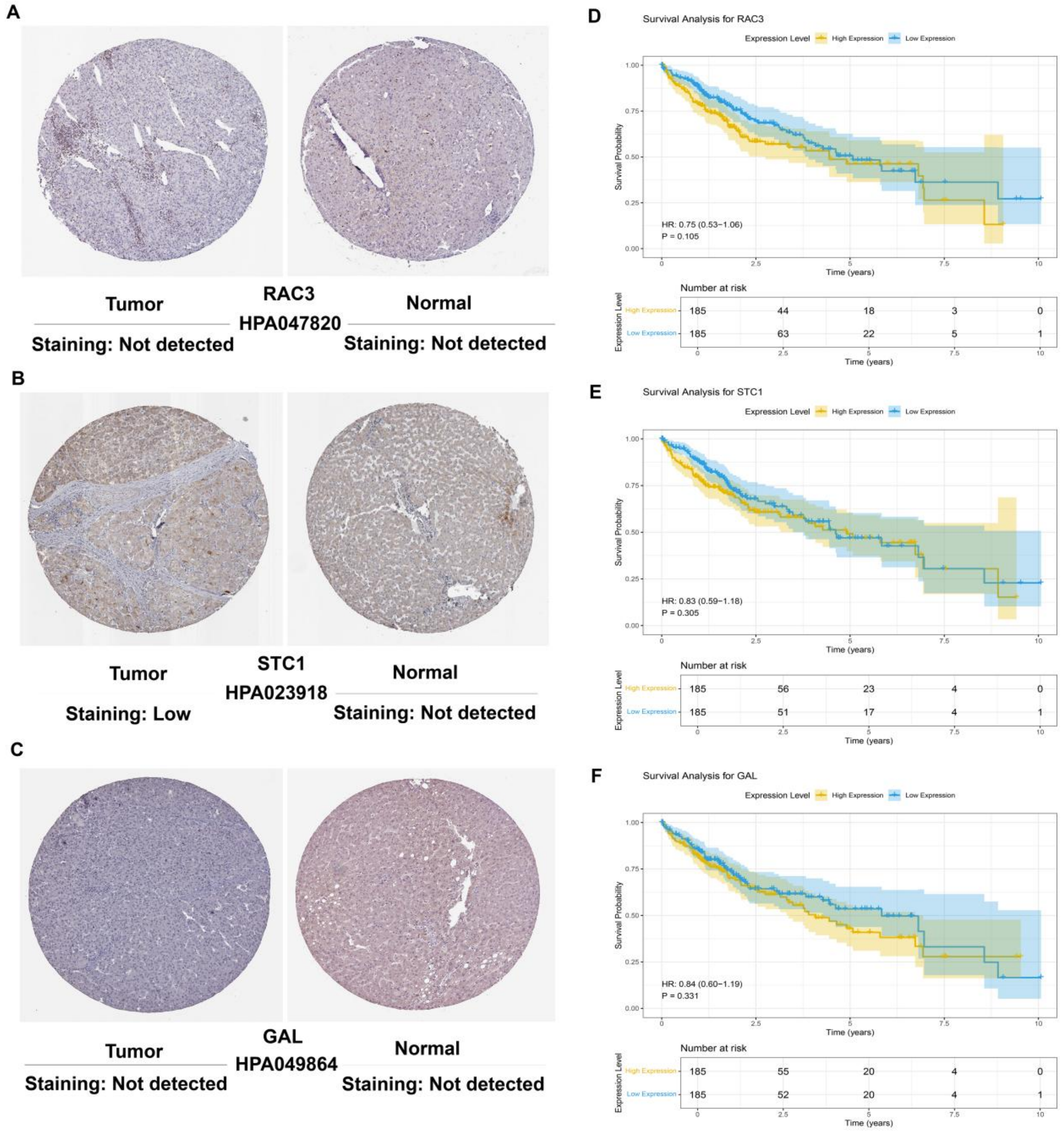

Supplementary Figure S9

The survival analysis of the characteristic genes of NMIRGs and the differential expression of NMRIGs characteristic proteins in liver cancer. **(A-C)** The protein expression levels of *RAC3, STC1, GAL* in HCC patients and normal tissues by immunohistochemistry (IHC) obtained from HPA online database. **(D-F)** The survival analysis of *RAC3, STC1, GAL* by TCGA database.

Supplementary Table S1. The sequence of primer.

HSP90AA1	Forward:5'-CCCAGAGTGCTGAATACCCG-3'<br>Reverse:5'-TAACAGGTGCCCTGCTTCTC-3'
HDAC1	Forward:5'-TCACCGAATCCGCATGACTC-3'<br>Reverse:5'-CTGCTTGCTGTACTCCGACA-3'
BTC	Forward:5'-CCACCACACAATCAAAGCGG-3'<br>Reverse:5'-CGTTTCCGAAGAGGGACACA-3'
GAL	Forward:5'-AGGAAAAACGAGGCTGGACC-3'<br>Reverse:5'-TTGCTGGTGAGGCCATTCTT-3'
GHR	Forward:5'-GGACGTACCAGCTGTTGTGA-3'<br>Reverse:5'-TGGATAACACTGGGCTGCTG-3'
CHGA	Forward:5'-ACTCCGAGGAGATGAACGGA-3'<br>Reverse:5'-TGGCTGCTCTGGTTCTCAAG-3'
MAPT	Forward:5'-CCAAGTGTGGCTCATTAGGCA-3'<br>Reverse:5'-CCAATCTTCGACTGGACTCTGT-3'
STC1	Forward:5'-GTGGCGGCTCAAAACTCAG-3'<br>Reverse:5'-GTGGAGCACCTCCGAATGG-3'
RAC3	Forward:5'-GACGTCAGTTTGGGGGTAGG-3'<br>Reverse:5'-TGGACGGGAAACCAGTCAAC-3'
Human Actin	Forward:5'-CACCATTGGCAATGAGCGGTTC-3'<br>Reverse:5'-AGGTCTTTGCGGATGTCCACGT-3'

Supplementary Table S2. The list of merged differentially expressed genes.

gene	conMean	treatMean	logFC	pValue	fdr	Regulation	GeneType
CALR	514.516416	1141.4118144385	1.14953038799888	7.00035127906398e-19	4.51666500334128e-18	Up	immune
CANX	143.518994	341.351737433155	1.25001741221709	1.63234418220616e-21	1.4506303958851e-20	Up	immune
CD1A	0.09548	0.688281550802139	2.84972836789438	0.000690576547701241	0.00101644235614776	Up	immune
CD4	64.366766	29.6729272727273	-1.117168727	6.64917527943e-16	2.9132665642898e-15	Down	immune
PDIA3	181.793308	375.922591176471	1.04813652375004	2.77579712744784e-21	2.37709172186897e-20	Up	immune
HLA-A	540.608924	1141.74965026738	1.07858911560177	2.97551741193543e-12	9.25061848859134e-12	Up	immune
HLA-DMA	23.520296	63.6454689839572	1.43615159374044	1.46305600432985e-06	2.71298967732031e-06	Up	immune
HLA-DOB	0.6242	2.32744919786096	1.8986694154921	8.8721399823564e-07	1.65824521098804e-06	Up	immune
HLA-F	23.027934	56.7168505347594	1.30039244000473	6.35375484014529e-10	1.61763163768023e-09	Up	immune
HLA-G	2.538168	5.99079465240642	1.23895981925909	5.07456092348521e-09	1.18911352983161e-08	Up	immune
HLA-H	44.25238	89.1055513368984	1.00976026652661	9.89621041232629e-08	2.01779874640939e-07	Up	immune
HSPA1A	60.57404	174.197884224599	1.52395556073425	4.7733595763554e-14	1.84282980365852e-13	Up	immune
HSPA1B	26.807594	74.2446577540107	1.46964547881888	4.78335633516825e-09	1.12648041693212e-08	Up	immune
HSPA1L	0.829518	1.90118021390374	1.19655010013824	4.81852378549477e-14	1.85267322691268e-13	Up	immune
HSPA2	2.203396	4.91823877005348	1.15841297320928	3.78394616155315e-08	8.0281217075895e-08	Up	immune
HSPA4	27.716274	70.6410227272727	1.34977290645768	7.15453977188869e-26	2.04229589852095e-24	Up	immune
HSPA5	287.380002	668.531580748663	1.21803604134019	3.21373774575995e-20	2.4218727652047e-19	Up	immune
HSPA6	2.943516	11.8150965240642	2.00501904072657	0.000154303847785756	0.000241452200355784	Up	immune
HSP90AA1	185.480376	425.69675802139	1.19855954661531	4.54599938921939e-19	3.05880816046048e-18	Up	immune
HSP90AB1	299.87518	1031.31323930481	1.78204856337052	1.18831882464723e-25	3.29234215534615e-24	Up	immune
LTA	0.291828	0.77257192513369	1.40455093967329	5.81503897064468e-06	1.01628324867297e-05	Up	immune
CIITA	0.704722	1.63758636363636	1.21644483612467	2.24975224640198e-06	4.05601901982221e-06	Up	immune
MICA	3.553656	10.7236754010695	1.59342351773757	2.8754957708071e-21	2.44028560009035e-20	Up	immune
MICB	1.202478	5.0502050802139	2.07033147510556	4.46144731827966e-16	1.97309078583072e-15	Up	immune
NFYA	5.75292	16.4309010695187	1.51404528637377	5.79591158450415e-18	3.26931060634905e-17	Up	immune
PSMC2	16.462102	33.599728342246	1.02930100745516	5.32116225803341e-24	8.95095508404906e-23	Up	immune
PSMC4	44.634222	118.401744385027	1.40746815174697	3.26421674589047e-28	2.1963515533063e-26	Up	immune
PSMC5	36.23158	77.0450644385027	1.08845481889583	9.33931367673371e-24	1.37463023179424e-22	Up	immune
PSMD1	8.324236	16.9752973262032	1.02804707128299	1.05254960939914e-21	1.00151690106464e-20	Up	immune
PSMD2	37.267972	91.3609176470588	1.29364082698529	2.25478111529292e-23	3.03429115800847e-22	Up	immune
PSMD3	34.961236	75.4236700534759	1.10926116256206	6.92119086779463e-24	1.0515744834617e-22	Up	immune
PSMD4	70.06908	248.939018449198	1.82894251557213	1.43575593134595e-28	1.6626475639806e-26	Up	immune
PSMD10	12.730806	36.3504120320856	1.51364795660255	3.75044863948574e-25	9.54843950917721e-24	Up	immune
PSMD11	16.003448	33.3155593582888	1.05781334382893	4.60654997936898e-23	5.29191473239705e-22	Up	immune
PSMD13	33.717428	68.8140949197861	1.02920960651329	1.63421038909722e-23	2.26386203901409e-22	Up	immune
PSME2	63.812828	128.535406684492	1.01024744592647	2.01826951854579e-17	1.04462081674183e-16	Up	immune
RELB	9.959528	24.7631072192513	1.31404307494885	1.39495830941033e-12	4.53120940505011e-12	Up	immune
RFX5	5.15475	19.9146577540107	1.94985632109084	3.58909577145586e-22	3.59673214543768e-21	Up	immune
TAP1	10.545178	33.0377176470588	1.6475305764631	3.51242163221436e-12	1.08482005821178e-11	Up	immune
TAP2	3.832558	9.80108903743315	1.35463443656885	9.69852805416332e-17	4.66123134031727e-16	Up	immune
TAPBP	88.744852	178.918390106952	1.0115663445116	5.30678434477882e-17	2.65903768764981e-16	Up	immune
SEM1	13.408808	32.3337548128342	1.26986006064959	1.73358264113309e-24	3.33272417948443e-23	Up	immune
AP3B1	7.178556	19.1680676470588	1.41693933122808	4.49482867598478e-23	5.29188836316911e-22	Up	immune
RFXANK	6.85733	24.9628256684492	1.86406239259843	1.28507862838902e-27	6.37128456811819e-26	Up	immune
PSME3	16.050576	40.0732069518717	1.32001289778255	7.42639695776984e-23	8.23019521672846e-22	Up	immune
PSMD14	8.264684	20.9656342245989	1.34299490907054	6.75024326695572e-24	1.04241461597906e-22	Up	immune
CLEC4M	14.954108	0.422407486631016	-5.145762642	2.99258088915283e-30	1.09397523244127e-27	Down	immune
ADRM1	33.7708	69.3332647058824	1.03777133862155	1.05655249442216e-24	2.26198284033108e-23	Up	immune
UBXN1	40.87974	86.6823481283422	1.08435221437105	4.07414599451377e-23	4.92031477798971e-22	Up	immune
ULBP2	0.429856	1.96254518716578	2.19080052318983	0.000161305643566849	0.000251989910845724	Up	immune
PDIA2	0.041502	4.65830080213904	6.81047912974294	9.05940002931877e-15	3.63147013941203e-14	Up	immune
HAMP	406.615996	45.5032227272727	-3.159626339	1.27852746218967e-24	2.61820188996232e-23	Down	immune
PI3	2.894282	20.8191553475936	2.84663413770585	0.000279386015545511	0.000428634571081224	Up	immune
CXCL14	39.738406	6.84299732620321	-2.537833715	2.82431784849674e-26	8.86835804427976e-25	Down	immune
CXCL9	18.377714	39.535013368984	1.10517359125252	0.0182809106784097	0.023589887478167	Up	immune
CXCL12	80.955124	21.418020855615	-1.918297225	2.99505262026159e-23	3.71228890564002e-22	Down	immune
CCL13	0.266178	1.71922807486631	2.69129770673302	6.30409915010623e-07	1.19246212839359e-06	Up	immune
CCL8	1.811408	3.96004304812834	1.12840457908598	9.58859998278844e-05	0.000153874977577286	Up	immune
TMSB10	765.30451	3205.52943368984	2.06645684912376	1.89343959362876e-13	6.70533871127177e-13	Up	immune
LCN2	9.982266	377.755545721925	5.24194177690056	4.83809957230033e-11	1.37273789069485e-10	Up	immune
COLEC10	26.292222	2.58038155080214	-3.34897976	2.92635702684777e-28	2.12048332253123e-26	Down	immune
S100A12	3.644966	0.773551336898396	-2.236336413	3.77481321187241e-16	1.70147509680632e-15	Down	immune
MMP12	0.156244	5.56396577540107	5.15424085138879	2.07523047921555e-10	5.58533460406014e-10	Up	immune
TMSB15A	0.120034	1.39190989304813	3.53555080297624	1.81492494251667e-12	5.81516767296157e-12	Up	immune
S100A13	4.475538	12.1835764705882	1.44480467352045	7.28830516660396e-08	1.50231585709867e-07	Up	immune
S100A6	73.865082	290.879957754011	1.97745946501639	8.10743351552878e-06	1.39111154310166e-05	Up	immune
S100P	4.194644	389.878821122995	6.53833360286536	4.50183891571457e-08	9.50836829283212e-08	Up	immune
S100A10	100.919806	423.738989037433	2.06996654183784	2.72407957172123e-19	1.88682570335397e-18	Up	immune
S100A2	0.817986	1.80694518716578	1.14340468692309	7.06957210020141e-07	1.33190738367795e-06	Up	immune
DEFB132	0.225854	2.24444919786096	3.31289907179715	0.0011413738744092	0.00164904016824151	Up	immune
LCN12	3.513258	7.88658342245989	1.16659091367494	1.59326511669661e-12	5.13991691756235e-12	Up	immune
S100A11	98.249108	360.354053208556	1.87489885828681	2.73670010500305e-06	4.89178652545137e-06	Up	immune
S100A3	0.346316	2.16749732620321	2.64586926622326	3.26917570871921e-18	1.90096513432932e-17	Up	immune
COLEC12	0.206478	2.15409545454545	3.38302220411733	6.50378240852394e-10	1.64693315203524e-09	Up	immune
MAVS	5.777512	18.6039628342246	1.6870897065435	1.41526267021174e-24	2.83654773476481e-23	Up	immune
ZC3HAV1L	0.34901	1.33078930481283	1.93094189791513	0.0304721090791262	0.0380699293800224	Up	immune
IL6	1.810086	0.742905080213904	-1.284808447	0.000185122333233819	0.000287764418987224	Down	immune
TGFB1	13.396508	37.568122459893	1.48765202661998	0.000384398204172756	0.000576597306259134	Up	immune
MMP9	2.571246	18.1108318181818	2.81631326029839	1.32732688945694e-08	2.96289556840862e-08	Up	immune
FABP6	0.024464	1.38752780748663	5.82571256281066	1.76548394138928e-06	3.22929295687127e-06	Up	immune
MBL2	138.278596	62.4008606951872	-1.147940027	1.07781728607971e-12	3.55001357862618e-12	Down	immune
PLAU	2.005688	9.80020935828877	2.2887153680898	2.27861732371286e-16	1.05736823592981e-15	Up	immune
PAEP	0.02107	29.4547002673797	10.4490918426395	2.38833465218276e-11	6.94386185912395e-11	Up	immune
IRF3	15.269156	42.6139213903743	1.48070449674763	2.20473373603333e-24	3.99395996027576e-23	Up	immune
LPA	31.505268	9.44254732620321	-1.738345067	1.36661225729276e-21	1.24985315181532e-20	Down	immune
NOX4	0.056186	0.701247593582888	3.64164131438119	1.83790647497879e-28	1.6626475639806e-26	Up	immune
LTF	0.642466	2.82654973262032	2.13735006872186	0.0230852117670092	0.0295867612034322	Up	immune
FABP5	1.129112	6.50232727272727	2.52576757259836	6.01884965710474e-20	4.46437509999422e-19	Up	immune
FABP4	2.70327	26.3656516042781	3.28588213294781	6.92122713174544e-10	1.74326095136476e-09	Up	immune
RBP7	5.920068	35.1173620320856	2.56849882330838	4.49482867598478e-23	5.29188836316911e-22	Up	immune
DUOX1	0.145236	1.4429871657754	3.31258746116293	1.88399261967628e-20	1.50400088791106e-19	Up	immune
RBP2	0.120584	4.14433743315508	5.10303107512945	1.32337444287074e-05	2.23809465921766e-05	Up	immune
CETP	21.016002	4.63228689839572	-2.181691729	1.83722048135879e-21	1.60246453096294e-20	Down	immune
PI15	0.031434	1.06472513368984	5.0820113585409	8.04420012890484e-13	2.67761007824324e-12	Up	immune
NOX1	0.372312	1.21383983957219	1.70499405575971	9.16139339096584e-17	4.44847039911846e-16	Up	immune
APOD	0.937546	3.73533048128342	1.99427450819281	7.2265909899373e-06	1.25136924862517e-05	Up	immune
ORM1	15128.34684	6623.38016256684	-1.191614773	4.15429785521998e-13	1.43346101817481e-12	Down	immune
PML	3.748324	8.64696844919786	1.20594876227414	2.79562005875034e-18	1.6548808538986e-17	Up	immune
AEN	5.959556	12.7550010695187	1.09778626405442	5.98168267088495e-14	2.27207462740872e-13	Up	immune
BPIFA2	0.003122	1.04166497326203	8.38220509105412	2.79180480504679e-09	6.69878612963557e-09	Up	immune
ISG20L2	7.472068	19.8925951871658	1.41265201283463	2.55126358858203e-23	3.24768959519496e-22	Up	immune
DUOX2	0.40431	15.6121631016043	5.27106474275779	0.0364231340504534	0.0449680108460381	Up	immune
TFRC	9.85557	32.865513368984	1.73756330302896	7.88224974534507e-17	3.86722878130992e-16	Up	immune
IDO1	0.961522	2.97730534759358	1.63061541713737	4.48828901967351e-05	7.4044978222985e-05	Up	immune
SOCS3	105.6578	37.2590160427808	-1.503737795	6.46900059141807e-09	1.50093560520094e-08	Down	immune
CCL20	17.66611	75.6567085561497	2.09848361359436	3.5289421258244e-08	7.53801243203307e-08	Up	immune
PPIA	48.618332	130.605197860963	1.42564001151445	1.94152050995611e-28	1.6626475639806e-26	Up	immune
ZYX	43.45121	100.189351871658	1.20526092908308	4.07958344413195e-13	1.41285573690158e-12	Up	immune
PGC	0.06843	181.372951871658	11.3720427551331	1.92913117482771e-12	6.16014090402611e-12	Up	immune
IKBKE	1.304974	5.35666871657754	2.03731501211613	2.30755653118517e-12	7.29435655159876e-12	Up	immune
FCN2	112.91046	5.70939331550802	-4.305697881	1.10846018880896e-28	1.6626475639806e-26	Down	immune
MAPT	0.110614	1.60932540106952	3.86285016568261	1.84593370081611e-25	4.96819870333936e-24	Up	immune
LYZ	48.739408	376.02093315508	2.94765234626275	3.64637337959646e-07	7.06766198267462e-07	Up	immune
ITGAV	8.747058	26.6693516042781	1.60831298474968	6.97520169845084e-10	1.75217066665085e-09	Up	immune
IRF5	2.979812	7.70539465240642	1.37064753796345	1.85442856061974e-17	9.65122488455135e-17	Up	immune

CACYBP	9.801896	36.0205318181818	1.87768673712368	5.92538789548288e-27	2.5371433625204e-25	Up	immune
NOD1	0.679218	1.53359732620321	1.17497312934597	1.28969605967449e-10	3.51125343414269e-10	Up	immune
MAPK3	9.598358	27.3005328877005	1.50806958296235	6.5302048752756e-26	1.92232906015925e-24	Up	immune
GRN	114.035408	290.107229411765	1.34710439681045	1.1398944344915e-20	9.33722223731298e-20	Up	immune
GNAI1	1.81633	4.04957245989305	1.15674325863629	4.13999543629129e-09	9.79868266579496e-09	Up	immune
WNT5A	1.224396	3.62235374331551	1.56485720367177	0.00981474626195139	0.0130955962871929	Up	immune
ADAR	37.76356	85.1495874331551	1.1730047646859	2.4929720773353e-17	1.26939443072965e-16	Up	immune
TYK2	12.484864	28.0955751336898	1.1701628276585	2.85541973864142e-22	2.92370151500024e-21	Up	immune
NOS2	0.232656	1.08153262032086	2.21680688080531	4.3642750149903e-14	1.69183006753945e-13	Up	immune
TRAF3	2.167676	5.49524893048128	1.34203570074949	7.8497777973978e-16	3.42337531719848e-15	Up	immune
TPM2	6.662346	42.208271657754	2.66342356959735	6.46106281539351e-24	1.02075637697397e-22	Up	immune
NEO1	5.68713	11.6137542780749	1.03006172735726	2.38275088847845e-06	4.28349491783721e-06	Up	immune
SRC	3.859454	15.7513655080214	2.02900823545864	5.47529382476553e-11	1.52662176185075e-10	Up	immune
ELAVL1	11.218098	23.1017254010695	1.04217251498271	1.25402948626451e-22	1.34238156370587e-21	Up	immune
ROBO3	0.594828	1.82774839572193	1.61952302021537	2.24464330680668e-10	6.02408545587434e-10	Up	immune
DLL4	2.34908	8.79437219251337	1.90448474466115	3.72088778270599e-22	3.68955399085162e-21	Up	immune
ECD	8.986208	18.5800053475936	1.04796655612959	2.05395439524638e-20	1.62590339522865e-19	Up	immune
DMBT1	0.013948	2.63256470588235	7.56026691227646	2.25190864900957e-06	4.05601901982221e-06	Up	immune
SKIV2L	13.758062	33.4351050802139	1.28108638924542	8.95337852365758e-25	2.00811489744891e-23	Up	immune
DCK	3.425558	9.82893636363636	1.52069629167657	1.33592053871342e-16	6.29218573734021e-16	Up	both
DAXX	17.163738	46.1326636363636	1.42642481002793	1.82366654949727e-24	3.43578777925286e-23	Up	immune
EED	1.896006	4.98242941176471	1.39388583620408	4.87391182912012e-24	8.50226841302065e-23	Up	immune
APOBEC3H	0.50093	1.23707540106952	1.30425251650845	1.51569198523823e-08	3.3753708040057e-08	Up	immune
SPINK5	0.070168	1.09816336898396	3.9681356417147	6.33469984590623e-17	3.1406775025493e-16	Up	immune
MARCO	96.16969	5.21556577540107	-4.204686601	1.89852513447382e-27	8.94205338337169e-26	Down	immune
CCL28	1.136524	4.64280748663102	2.03036931109743	0.000216234449478749	0.000334471020375996	Up	immune
LTB4R	1.673964	4.28852967914438	1.35721460316697	2.84816331718879e-12	8.91352107904266e-12	Up	immune
TRIM27	11.059656	27.4832842245989	1.31324790242833	9.91399374577524e-25	2.17185630430704e-23	Up	immune
PTX3	0.467668	1.2711077540107	1.44252971388711	0.0284107534857847	0.0357315484427359	Up	immune
IFNAR2	0.346416	0.745062299465241	1.10485549794764	7.75969385200921e-15	3.13718094789385e-14	Up	immune
SYTL1	0.793424	1.7705385026738	1.15802427453485	0.000465998803343234	0.000695674917193861	Up	immune
PTGS2	1.825516	0.626673796791444	-1.542517738	2.19202249389068e-15	8.97776169236966e-15	Down	immune
MAP2K2	24.324644	65.8478435828877	1.43671750438364	2.60991097922357e-26	8.7804862229593e-25	Up	immune
NDRG1	14.618572	61.8794077540107	2.08165699885258	7.81153096663691e-13	2.60938374842978e-12	Up	immune
IRF9	0.594308	1.38321524064171	1.21874296382831	1.22959928523149e-09	3.05615442397906e-09	Up	immune
LANCL1	10.312154	24.4915612299465	1.24793902839244	1.39254860804005e-17	7.45329993621436e-17	Up	immune
ABCC4	0.880746	4.88243529411765	2.47080300180904	5.9234559022061e-10	1.52814557670497e-09	Up	immune
HGF	6.685994	2.8571	-1.226590528	9.85022796218208e-19	6.14497664925531e-18	Down	immune
HDAC1	25.467054	57.1031379679144	1.16493794772723	6.26814912807702e-19	4.10041422128372e-18	Up	immune
STAB2	5.325608	0.298881016042781	-4.155303071	6.26939891573481e-28	3.69110861163887e-26	Down	immune
PDGFRB	7.581736	29.9875168449198	1.9837619358232	4.08585722269487e-17	2.05822326405271e-16	Up	immune
PDCD1	0.77962	3.7577743315508	2.26903542371431	0.0062912114310992	0.00849043147291611	Up	immune
PCSK1	0.03453	5.61269251336898	7.34469888021574	7.8564433980936e-06	1.35545232252824e-05	Up	immune
ARG2	1.051558	4.35493663101604	2.05012330030342	1.05392257988156e-05	1.79205603187044e-05	Up	immune
BIRC5	0.652138	20.672063368984	4.98636129957754	1.60240989599582e-28	1.6626475639806e-26	Up	immune
VIM	35.433646	84.0654328877005	1.24639277496072	9.72382819541354e-12	2.88954137541942e-11	Up	immune
PRDX1	194.46893	390.759190106952	1.00674012731512	8.86004390154524e-14	3.26021927939672e-13	Up	immune
GBP2	8.955072	37.5172106951872	2.06677563446653	2.43606235812708e-19	1.69983017878201e-18	Up	immune
ALB	87922.60573	39494.30905	-1.154589361	1.14876257583317e-18	7.0727735061101e-18	Down	immune
AGER	1.369944	3.58141631016043	1.38641330932262	4.04327092901798e-17	2.04772108340588e-16	Up	immune
UNC93B1	10.509884	22.7463751336898	1.11388990946311	8.14652663157208e-14	3.03321268258534e-13	Up	immune
TNFSF4	0.498132	3.02682459893048	2.60320507720778	5.86568232967805e-19	3.89117799616671e-18	Up	immune
ACTA1	0.128128	0.624713368983957	2.28560861978066	3.63607682545546e-12	1.11569523439057e-11	Up	immune
CCL15	2.723552	8.65130614973262	1.66742855080472	2.38999172738865e-12	7.52967293377963e-12	Up	immune
CCL26	0.116978	1.8295577540107	3.96718582481196	6.87244033335895e-10	1.73561361770084e-09	Up	immune
CCL23	5.26949	1.12050106951872	-2.233519314	9.82701255291517e-23	1.07103293921787e-21	Down	immune
CCL25	0.421096	69.6412740641711	7.36964961525502	3.76768886128773e-11	1.08051536497542e-10	Up	immune
PPARG	5.122856	12.897656684492	1.33208872857872	6.69657061393548e-12	2.01538962246876e-11	Up	immune
PTK2	5.767908	16.2267614973262	1.49225504017332	1.43324404481227e-22	1.51698414630692e-21	Up	immune
VDR	0.786536	2.63240935828877	1.74279915093951	3.67741366437215e-06	6.4871229809711e-06	Up	immune
RAC1	91.55426	184.655250534759	1.01213536410103	1.33463730441937e-21	1.23257680466965e-20	Up	immune
RAC3	9.566494	31.4537660427807	1.71717056808915	9.45714758081114e-14	3.45295853531942e-13	Up	immune
NFATC4	0.571808	2.49149679144385	2.12341000656513	9.153750553865e-14	3.35518794620266e-13	Up	immune
HRAS	11.60928	37.5062032085562	1.69185072492219	9.81974437715734e-27	3.85424966803426e-25	Up	immune
NRAS	15.63903	36.0249764705882	1.20384645485628	7.88224974534507e-17	3.86722878130992e-16	Up	immune
FOS	304.673068	61.8981697860963	-2.299293319	8.26968275368427e-20	6.03879159222526e-19	Down	immune
IKBKG	2.79391	8.6102807486631	1.62377473113777	7.25000486197586e-15	2.94375197412985e-14	Up	immune
NFKBIE	10.372924	24.9699139037433	1.2673682173517	3.77377447003094e-11	1.08051536497542e-10	Up	immune
PIK3R3	1.77143	4.71638395721925	1.41276671489593	1.22812684901755e-19	8.7643597861707e-19	Up	immune
FCGR2B	4.728944	2.34811310160428	-1.010016157	2.36482855128739e-17	1.21068939962648e-16	Down	immune
RASGRP3	1.354168	2.96946631016043	1.13279693193322	3.28670405491861e-10	8.74597519698681e-10	Up	immune
IGHA1	490.761748	204.73284973262	-1.2612802	4.97936488485897e-13	1.69947888461491e-12	Down	immune
IGHA2	42.749208	21.0984962566845	-1.018757514	6.50381244752768e-10	1.64693315203524e-09	Down	immune
IGLV8-61	64.494986	26.0807082887701	-1.306203956	2.63025288453199e-09	6.336824084985e-09	Down	immune
CXCL17	0.182366	5.58994438502674	4.93792524347744	1.89109069339692e-07	3.79022858123383e-07	Up	immune
CCN1	92.90574	40.0416732620321	-1.214265471	1.10702680012267e-12	3.62090015873457e-12	Down	immune
SAA1	9636.042942	3355.98031737968	-1.521706568	6.07488044176275e-06	1.05777030982265e-05	Down	immune
SAA2	466.230846	151.145103208556	-1.625110217	2.07071378899647e-05	3.45241130837996e-05	Down	immune
SEMA3B	1.74327	8.61979278074866	2.30585715412169	1.49601163609899e-07	3.02412652619152e-07	Up	immune
SEMA3F	3.06378	12.6503697860963	2.04579494642005	1.02820954078009e-29	2.42143346853711e-27	Up	immune
SEMA3G	1.634832	6.06745561497326	1.89194926184054	3.91039638488957e-16	1.74577885998387e-15	Up	immune
SEMA4F	0.578268	2.71624438502674	2.23180311302529	1.35827408803489e-13	4.88356561423231e-13	Up	immune
SEMA5B	0.189246	1.77822807486631	3.23210566345287	3.99715024022428e-27	1.79300739347203e-25	Up	immune
SEMA6B	2.824952	6.38807647058823	1.17715522884094	7.07702097237901e-13	2.37243905906798e-12	Up	immune
SEMA6C	2.10125	9.93951604278075	2.24192778889777	2.06753172475361e-21	1.78680264653018e-20	Up	immune
SEMA7A	2.025196	7.88002994652406	1.96013957250351	2.87319894011612e-12	8.96209735625624e-12	Up	immune
TYMP	29.111328	65.2499013368984	1.16439506641674	2.82002907826866e-08	6.12089260767069e-08	Up	both
CXCR3	1.25699	3.1295628342246	1.31598797005376	6.03967613972857e-06	1.05358794881932e-05	Up	immune
EDNRA	1.205382	2.92415267379679	1.27852821196847	0.000108811273569646	0.000172850286176402	Up	immune
FPR1	5.753618	2.86496363636364	-1.005952612	4.91866792489773e-11	1.39140696253864e-10	Down	immune
GPR17	0.248842	0.598785828877005	1.26681007167693	0.0282573681494522	0.0355861507978395	Up	immune
PTGDR2	0.454382	3.13289411764706	2.78551842198864	1.09231957524078e-12	3.58524404138263e-12	Up	immune
C5AR2	1.098882	3.2849871657754	1.5798512587275	0.00033631665547189	0.000510161496706152	Up	immune
PLXNA1	1.408116	6.4023128342246	2.18482698508817	1.06710441809392e-17	5.81047608002585e-17	Up	immune
PLXNA2	2.014754	5.62415588235294	1.48103288404387	1.60319097785835e-20	1.301901638916e-19	Up	immune
PLXNA3	0.866386	4.24038101604278	2.2911120671974	1.63623485450092e-15	6.82005855283127e-15	Up	immune
PLXNC1	0.776404	4.70658181818182	2.59980021876062	4.88706238963891e-16	2.15122092104666e-15	Up	immune
PLXND1	8.650002	25.9271048128342	1.58368874315127	4.06151010147519e-18	2.34720399729425e-17	Up	immune
ROBO1	1.540018	19.9352470588235	3.69430236625667	4.1595654069659e-19	2.81892849882149e-18	Up	immune
ADM2	1.605472	12.4334299465241	2.95315493089109	2.17328660118175e-17	1.11870818487061e-16	Up	immune
AMH	0.071102	0.803662032085562	3.49862687883022	3.03588159766844e-08	6.55917537844879e-08	Up	immune
APLN	0.284892	7.9210486631016	4.7972044252319	5.62329417111631e-29	1.05942862183831e-26	Up	immune
MANF	38.421248	129.745656417112	1.75570995478034	3.83928953211871e-24	6.8237938476525e-23	Up	immune
BMP10	3.363084	0.198074064171123	-4.085672922	3.48399755554544e-30	1.09397523244127e-27	Down	immune
BMP2	5.306964	10.8218975935829	1.02799482711783	0.00482914670504811	0.00658329406100625	Up	immune
BMP4	2.019404	11.9437794117647	2.56425795704535	5.5849338199728e-09	1.30546095742292e-08	Up	immune
BMP5	1.591246	0.569996256684492	-1.481132538	3.3357655501251e-20	2.49388186366496e-19	Down	immune
BMP7	0.02749	0.965286363636364	5.13397818649106	0.000135405893555578	0.000213298247039054	Up	immune
BMP8A	0.143046	0.953522192513369	2.73678736047677	9.21691037423748e-16	3.9646261218148e-15	Up	immune
BMP8B	0.43529	2.10052165775401	2.27069887872042	0.00267202814856387	0.00375119301929533	Up	immune
BTC	0.729968	1.53637379679144	1.07362413740293	0.0259345960272134	0.0330140398076149	Up	immune
MYDGF	105.460812	236.60835802139	1.16579402678568	3.60273604194657e-23	4.40750305391386e-22	Up	immune
CD320	11.861906	33.8715836898396	1.51373959836582	2.89004454295143e-22	2.92733544027984e-21	Up	immune
CD70	0.15697	0.949938235294118	2.5973448543203	4.41553217861261e-06	7.77463796682818e-06	Up	immune
CHGA	0.020206	6.8225486631016	8.39938321697539	5.17260562193597e-13	1.75273183304449e-12	Up	immune
CHGB	0.021528	1.28606550802139	5.90060602444494	0.0345414594566465	0.0427569708385821	Up	immune
CMTM3	5.939232	15.775272459893	1.40931662886997	7.88111321491186e-09	1.81073381669438e-08	Up	immune

CMTM4	1.083082	5.10883502673797	2.23785187599268	1.21434674487267e-19	8.73217277610729e-19	Up	immune
CMTM7	3.68705	7.56451417112299	1.03678044779989	2.68782323505029e-06	4.81355415858816e-06	Up	immune
CSPG5	0.102774	1.08753422459893	3.40351356361794	6.73241355095412e-22	6.60618079687373e-21	Up	immune
DKK1	0.258452	24.2315371657754	6.55084575065128	1.05833014835189e-08	2.37934844808468e-08	Up	immune
EGF	0.01119	0.621298395721925	5.79500438762004	1.75823506683904e-06	3.22229072560773e-06	Up	immune
ESM1	0.361876	12.4646101604278	5.10619852217502	1.15354070841583e-27	6.03686304070951e-26	Up	immune
FAM3B	1.23351	16.9722106951872	3.78233317700252	0.0311856212456275	0.0389097420044783	Up	immune
FGF12	0.302374	1.86030561497326	2.6211336517227	1.51309382304614e-11	4.44029402277092e-11	Up	immune
FGF13	0.154066	0.977233957219251	2.66515547933677	6.08727666292728e-10	1.55398770094241e-09	Up	immune
VEGFD	0.691992	6.82339438502674	3.30166234064356	3.1920568272335e-07	6.21263952738421e-07	Up	immune
FIGNL2	0.093128	0.570676203208556	2.61538550400433	1.65774568854017e-12	5.32968067783222e-12	Up	immune
GAL	0.082028	1.12939946524064	3.78329558988739	0.0108964831239727	0.0144366907212128	Up	immune
GDF11	0.67353	1.73837754010695	1.36792732813212	0.00244407066719752	0.00345174597976022	Up	immune
GDF2	20.153004	1.20126818181818	-4.06836473	1.44874410562605e-30	1.09397523244127e-27	Down	immune
GMFB	6.785214	15.3418553475936	1.17700674099056	3.42656371461598e-16	1.56690437823702e-15	Up	immune
GNRH1	0.241366	0.865369518716578	1.84209383968836	5.52199882530848e-18	3.13356800809674e-17	Up	immune
GPI	37.859724	85.8145518716578	1.18055841754568	3.14929754193964e-19	2.16542940474974e-18	Up	immune
GREM1	0.314262	1.07512459893048	1.7744641274233	0.0290848396283806	0.0364334028323597	Up	immune
HDGFL3	0.419084	0.928887700534759	1.14826474748685	0.00205759945362531	0.00292788321044568	Up	immune
IL11	0.050866	1.67785695187166	5.04377426371405	1.66140798002342e-09	4.07564145099495e-09	Up	immune
IL17D	0.14189	2.14843422459893	3.92044078786147	1.05247508007826e-13	3.82792094762054e-13	Up	immune
IL1RN	110.332854	41.8760868983957	-1.397663907	8.59812278116564e-10	2.15410416485586e-09	Down	immune
IL32	130.534436	345.691867112299	1.40505621138399	7.01178775497701e-09	1.61889805519322e-08	Up	immune
IL34	2.207242	6.40322032085562	1.53655283675151	7.00088421148349e-06	1.21675884266005e-05	Up	immune
INHA	0.090228	4.37016336898396	5.59796819526738	1.96934477762707e-07	3.93034487399301e-07	Up	immune
JAG1	4.015446	12.6818080213904	1.65912829896602	3.3370169587367e-08	7.1605238613439e-08	Up	immune
JAG2	0.971284	5.61448930481283	2.53118969937501	5.52503025540044e-24	9.13083947471441e-23	Up	immune
KITLG	0.943398	4.02192112299465	2.09194634063695	8.69826260618077e-13	2.88512794895151e-12	Up	immune
LTB	7.246112	25.0886871657754	1.79175796956986	4.92229659504025e-05	8.05000588980541e-05	Up	immune
LTBP2	2.487234	10.5471914438503	2.08424473399926	8.47673796224173e-09	1.93812795156109e-08	Up	immune
MDK	10.65412	230.466239572193	4.43507208866554	7.20763435999506e-25	1.65599794319887e-23	Up	immune
NENF	61.345604	165.231584491979	1.42945761863008	2.4890311880029e-23	3.21187312205306e-22	Up	immune
NMB	1.817932	14.2583363636364	2.97143551916271	3.97280868009012e-26	1.20722121827255e-24	Up	immune
CCN3	0.73333	3.17103903743316	2.11242117270169	7.51032953592635e-11	2.08080306554195e-10	Up	immune
NRG1	1.67894	0.756795187165775	-1.149575855	3.8551088780214e-11	1.10045835245338e-10	Down	immune
NRG2	0.096322	0.78060935828877	3.01866350535243	1.02369174451102e-10	2.81142164235971e-10	Up	immune
NTF3	3.707252	0.552910160427807	-2.745233197	7.39668568100783e-27	3.02942517891712e-25	Down	immune
OSGIN1	36.96134	111.297776203209	1.59033580062908	9.3168005718209e-09	2.10971782179214e-08	Up	immune
PDGFA	2.728222	18.2566994652406	2.74239302271544	1.32891988187846e-19	9.4123498400715e-19	Up	immune
PDGFB	3.59684	11.8284264705882	1.71745627557106	7.47957700195464e-19	4.79303505839542e-18	Up	immune
PDGFD	2.572946	9.61430962566845	1.90176208708021	0.000278259081606157	0.000427602047101142	Up	immune
PDGFRL	0.360926	2.40157192513369	2.73420403736118	9.6936663720614e-18	5.34009806022089e-17	Up	immune
PGF	1.151898	4.38403155080214	1.92824520855755	6.06648525262284e-14	2.29503177026936e-13	Up	immune
PTHLH	0.316492	2.87313368983957	3.18238418642328	2.93731073235757e-06	5.22065416958647e-06	Up	immune
RABEP2	4.267634	9.77675053475936	1.19591858787847	5.37552168768535e-18	3.06893419987855e-17	Up	immune
REG1A	0.094746	175.437512566845	10.8546046049631	1.24469295276693e-08	2.79166847977726e-08	Up	immune
SCGB3A1	3.280846	1.22629973262032	-1.41975623	2.96174905112893e-08	6.41371863485851e-08	Down	immune
AIMP1	6.426722	14.6343320855615	1.18720192996264	5.18940797227259e-24	8.8880405634196e-23	Up	immune
SECTM1	6.548122	17.4792631016043	1.4164912579003	9.74582374332401e-07	1.81793385469529e-06	Up	immune
SPP1	24.056886	895.678341176471	5.21845890744307	7.85630227156479e-08	1.61585955017774e-07	Up	immune
STC1	2.695746	9.93341684491979	1.88160548528695	7.01487042390606e-14	2.62222537274584e-13	Up	immune
STC2	0.708782	5.76362887700535	3.02356357138499	3.80560475698071e-19	2.59773889933031e-18	Up	immune
TDGF1	1.001626	8.86083689839572	3.14509904946883	0.000103186193538421	0.000164469364320123	Up	immune
TGFB2	0.524998	2.44877112299465	2.22167410527224	3.05584552528453e-05	5.08587718165729e-05	Up	immune
TNFSF15	0.151318	1.05545427807487	2.80220856462142	1.88378248857056e-09	4.59721011459447e-09	Up	immune
TNFSF9	0.363272	1.60021042780749	2.13913955589638	0.000105282003080454	0.000167526430577344	Up	immune
TOR2A	4.96727	9.97580588235294	1.0059802244949	6.40822681877424e-19	4.16313769881747e-18	Up	immune
TSLP	3.51281	1.12687192513369	-1.640301992	5.30564604082519e-19	3.54462309961513e-18	Down	immune
UCN	1.038734	4.06824278074866	1.96957952274896	5.95198889533564e-17	2.96654684624665e-16	Up	immune
UTS2	0.09463	0.664628877005348	2.8121794466298	0.000283135442642936	0.000433680629218936	Up	immune
VEGFB	24.820328	67.7953160427808	1.44966342068245	1.1734876962433e-05	1.98817519759207e-05	Up	immune
VGF	0.049756	0.719846524064171	3.85474693377518	3.14642105863878e-10	8.39639840577261e-10	Up	immune
ACVRL1	2.823956	7.6443679144385	1.43667960501952	7.81691783914699e-19	4.97536257059221e-18	Up	immune
ADIPOR1	62.368984	133.441022994652	1.09730159151922	2.45847138709606e-23	3.21187312205306e-22	Up	immune
ANGPT1	0.414156	1.34885240641711	1.70348630026928	6.2818460392981e-09	1.46111085654785e-08	Up	immune
ANGPTL6	14.36952	2.81951737967914	-2.34949173	3.59112725133689e-28	2.25522791383957e-26	Down	immune
AVPR1A	32.143062	10.2849957219251	-1.643966175	1.18739907690612e-14	4.7395336035829e-14	Down	immune
BRD8	4.73295	13.1252727272727	1.47153581424731	1.44352129895653e-23	2.02954785614485e-22	Up	immune
CALCRL	3.409628	7.56586604278075	1.14989088726806	7.43781073870545e-06	1.28558123226799e-05	Up	immune
CRLF1	0.183896	2.55581096256685	3.79681912852486	7.27357280259595e-07	1.36488158965048e-06	Up	immune
CRLF3	1.931066	4.08715053475936	1.08169790698029	1.26128589740783e-13	4.55222726190872e-13	Up	immune
ESR1	5.554254	1.55775106951872	-1.834128447	1.14380424712671e-21	1.07746360079336e-20	Down	immune
FGFR3	16.77431	51.2438475935829	1.61112537972348	1.93223672975983e-07	3.86447345951966e-07	Up	immune
FGFR4	40.581886	123.068566042781	1.60055449875458	1.20074305857634e-19	8.70076893214548e-19	Up	immune
FLT1	3.708908	8.35129679144385	1.17100575500117	5.47529382476553e-11	1.52662176185075e-10	Up	immune
GALR3	0.195444	0.846268983957219	2.11436099682049	3.16696629733404e-06	5.6182339964005e-06	Up	immune
GHR	68.676808	20.8970473262032	-1.716523877	7.46781630905897e-20	5.49584606494809e-19	Down	immune
GHRHR	0.083574	1.58457406417112	4.2448970962513	3.40873579658746e-06	6.02444487877183e-06	Up	immune
GIPR	0.121902	0.937566577540107	2.94319934544676	0.00069525047514681	0.00102172534725163	Up	immune
GLP1R	0.002828	0.932405882352941	8.365032176	5.39693272245875e-14	2.06663033518542e-13	Up	immune
HTR3A	0.016426	1.27420481283422	6.27747217839669	0.000424576130171356	0.000635851692561872	Up	immune
IFNGR2	17.936654	37.1532807486631	1.05057882353004	1.68255094187798e-10	4.54144122420933e-10	Up	immune
IL11RA	4.791438	10.6778098930481	1.15608516292933	1.8160652893337e-15	7.50321711645766e-15	Up	immune
IL17RB	19.625054	49.0803871657754	1.32245000589526	4.00395698375353e-09	9.50057299419603e-09	Up	immune
IL17RE	3.343912	7.5255807486631	1.17026603760706	0.0341392731455728	0.0423147306620126	Up	immune
IL1R2	3.402692	9.77433181818182	1.52232151249423	5.25427273463626e-11	1.47747012418727e-10	Up	immune
IL1RAP	17.701152	8.22977192513369	-1.1049189	6.32801941106477e-14	2.37489812160279e-13	Down	immune
IL1RL1	2.521174	0.411335294117647	-2.615708919	1.44646362855246e-13	5.18086972660235e-13	Down	immune
IL22RA1	7.207898	17.1754834224599	1.25270020519517	0.000254712993866192	0.000392058235656786	Up	immune
IL27RA	2.790712	7.19420080213904	1.36620118133727	5.39369863715176e-10	1.40355362325883e-09	Up	immune
IL2RA	0.463474	1.01423288770053	1.12982864755172	0.00402001445797279	0.00550414770263135	Up	immune
IL2RG	2.963994	8.16080347593583	1.46116867323673	0.00265589533588206	0.00373410956179239	Up	immune
IL6R	26.119188	56.5673385026738	1.11485924696463	4.99473418599833e-08	1.05258156671374e-07	Up	immune
LIFR	11.356592	2.77138155080214	-2.034852708	2.99500563816577e-23	3.71228890564002e-22	Down	immune
MC1R	0.159234	0.559997326203209	1.81427152256129	2.93960773197705e-13	1.02940910168118e-12	Up	immune
MCHR1	1.152606	4.31572272727273	1.90470274386864	0.000363418589482493	0.000548622293737994	Up	immune
NGFR	16.447464	7.33390347593583	-1.165211972	2.20972528725375e-16	1.03047585177873e-15	Down	immune
NR2C1	3.296744	6.80861711229947	1.0463199417687	2.31156064857285e-18	1.38693638914371e-17	Up	immune
NR2C2	1.502524	3.90643743315508	1.37846547221026	8.22950726265727e-12	2.46101455283275e-11	Up	immune
NR2F6	44.660188	90.2190088235294	1.01442211191698	1.48422164026512e-17	7.89907788208894e-17	Up	immune
NR4A1	21.601684	8.30369812834225	-1.379317883	2.24749559964118e-12	7.12842038674071e-12	Down	immune
NR4A2	13.312412	6.1321614973262	-1.118304391	4.50843151962637e-09	1.06439661440803e-08	Down	immune
NR4A3	4.513896	1.57098502673797	-1.522703749	3.57417553791442e-12	1.1002854106913e-11	Down	immune
NR6A1	0.689526	2.96746336898396	2.1055533633647	9.89170548959175e-23	1.07103293921787e-21	Up	immune
OXTR	0.388902	2.12978903743315	2.45323197463974	2.03031344121805e-08	4.45817077302425e-08	Up	immune
PPARD	10.233642	23.4656336898396	1.19722975086065	2.72615903949377e-12	8.56013938401044e-12	Up	immune
PTH1R	17.873374	3.21188342245989	-2.476320566	1.07747826284116e-26	4.05993809438549e-25	Down	immune
RARA	14.309316	31.2993778074866	1.12917926706038	1.30648662693034e-17	7.03263087181932e-17	Up	immune
RARG	1.607888	3.74419893048128	1.21949017100909	1.00175128572095e-08	2.26294894759984e-08	Up	immune
RXRB	13.535214	33.6237139037433	1.31276138847976	7.02584765976957e-25	1.65458712387573e-23	Up	immune
SORT1	4.462428	17.8162235294118	1.99729076700635	1.09011896634104e-17	5.90167854191529e-17	Up	immune
TACR1	0.042522	0.64150320855615	3.91517512116417	8.64613700186491e-05	0.000139224975312081	Up	immune
THRA	2.953452	7.9536949197861	1.42922306046138	1.45067054873085e-16	6.79866495972368e-16	Up	immune
'NFRSF11/	0.386544	1.2761550802139	1.72309910869357	6.81977163654861e-05	0.000110762497959117	Up	immune
TNFRSF18	0.644358	4.0918179144385	2.66680758101104	8.88753058637163e-15	3.57780077451371e-14	Up	immune
TNFRSF21	10.39268	29.6919529411765	1.51450425193887	3.28613172305519e-05	5.45949926475836e-05	Up	immune
TNFRSF25	0.849402	3.93811764705882	2.21298680008842	7.72312011823846e-22	7.50018469214498e-21	Up	immune

TNFRSF4	0.778074	6.18390668449198	2.99053927235564	2.71494530841676e-26	8.81889131216755e-25	Up	immune
TNFRSF9	0.21792	1.41571577540107	2.69966113985541	7.38762009828703e-05	0.000119572820147532	Up	immune
VIPR1	7.129792	1.00524518716578	-2.82631256	7.48184929893098e-28	4.14582472917234e-26	Down	immune
ICAM2	1.472438	3.11999090909091	1.08333493770202	1.29233186552954e-09	3.20362267718112e-09	Up	immune
PAK1	5.557384	14.755235828877	1.40874914403463	1.13729747368382e-15	4.80418932829667e-15	Up	immune
PLCG1	3.27944	11.6168558823529	1.82469826908333	1.65176536572228e-21	1.45417100421532e-20	Up	immune
SHC1	21.46337	69.8998914438503	1.70341360119747	2.86585137429621e-20	2.17712257627986e-19	Up	immune
GRB2	37.616478	76.2683398395722	1.01971951960662	8.04726829000729e-21	6.70843073379369e-20	Up	immune
HCST	1.791096	3.98508288770053	1.15376706621321	0.00139018922794481	0.00200238264942509	Up	immune
PRKCA	3.589108	11.169370855615	1.63785068337499	6.26812940262503e-19	4.10041422128372e-18	Up	immune
CASP3	11.04198	24.6626307486631	1.15932780626531	9.69380858065575e-18	5.34009806022089e-17	Up	immune
BID	14.643776	30.4996505347594	1.05850510176553	3.32357626047593e-16	1.5272238231065e-15	Up	immune
CD3D	7.46952	18.4027930481283	1.30083730328178	0.000706249286321639	0.0010346606962908	Up	immune
NCK2	7.438928	20.9606411764706	1.49451620965474	0.000382607226364352	0.000574826778578662	Up	immune
GRAP2	0.55849	1.17679411764706	1.07525858624463	0.0150255461017147	0.0195768525972548	Up	immune
PAK4	7.41858	18.2725962566845	1.30046666318814	1.11155013314839e-18	6.88868569359068e-18	Up	immune
RHOA	89.665258	179.376319786096	1.00036843956581	2.56180341594903e-22	2.65188881079559e-21	Up	immune
CD28	0.62697	1.27142326203209	1.01997607139796	1.55581645096926e-06	2.87933024914154e-06	Up	immune
ICOS	0.396422	0.833061497326203	1.07138597137857	0.00200654625650401	0.00285955608718121	Up	immune
MAP3K8	3.44475	7.42647406417112	1.10827812735745	0.000553370163869554	0.000818327620667378	Up	immune
CTLA4	0.467052	1.65452754010695	1.82476421713465	1.56953102151633e-06	2.89334290072091e-06	Up	immune
CBL	1.439862	2.91690909090909	1.01850987678827	2.12391956235208e-06	3.84017702060587e-06	Up	immune
CDK4	9.966292	30.3313181818182	1.60567944910014	1.51607883012234e-17	8.02329358413059e-17	Up	immune
PDK1	1.33824	3.1984435828877	1.25703316374287	3.82931568981599e-09	9.13219083495358e-09	Up	immune
TRAV4	0.499452	1.00705882352941	1.01173001626872	0.0232000499228449	0.0296935421566846	Up	immune
TRAV12-3	0.346562	0.724935026737968	1.06473822363041	0.0161603392321996	0.0209683740450854	Up	immune
TRAV13-2	0.239622	0.563974598930481	1.23486981611822	0.00707726393673705	0.00951038891356106	Up	immune
TRBV3-1	0.356772	0.99134385026738	1.47438315201342	0.0149577067197509	0.0195154566897581	Up	immune
TRBV6-5	0.50367	1.02588368983957	1.02631646612789	0.0258266420620766	0.0329211053078162	Up	immune
TRBV10-3	0.254442	0.549117647058823	1.10977844434017	0.0384076373956196	0.0472323687032293	Up	immune
TRBV11-2	0.264536	0.601489304812834	1.18507501658793	0.0290510600625817	0.0364334028323597	Up	immune
TRBV30	0.267308	0.734345454545455	1.4579558874966	0.0181185486891686	0.0234124456312714	Up	immune
PAPSS1	6.412126	17.3273323529412	1.43417487978398	9.20905320198036e-17	4.44868108526436e-16	Up	neither
NT5C	7.88389	21.2516762032086	1.43059708433758	1.50254073073698e-21	1.34799368414689e-20	Up	leotide_metabolism
NT5M	1.057054	4.48019090909091	2.0835111302345	1.30314010629244e-18	7.9711557151135e-18	Up	leotide_metabolism
ATIC	21.173082	56.010235828877	1.40345921624012	1.35661449315179e-23	1.93625886749846e-22	Up	neither
NME7	1.356406	3.20930828877005	1.24247331155232	1.72811601370874e-15	7.1713008145975e-15	Up	leotide_metabolism
NME1	4.499566	18.8093831550802	2.06359477720489	6.18283718199585e-25	1.49339298088207e-23	Up	leotide_metabolism
NME2	0.165672	0.558933422459893	1.7543466509743	1.30786733108845e-15	5.50004922270232e-15	Up	leotide_metabolism
NME3	23.151662	61.216321657754	1.40280059469999	9.10929449131613e-24	1.36205641441584e-22	Up	leotide_metabolism
NME6	2.447714	6.04293342245989	1.30381404931884	4.20988853990886e-25	1.0436092117353e-23	Up	leotide_metabolism
APRT	75.03955	153.0538	1.02831578297201	7.57962746326876e-18	4.25000539904713e-17	Up	leotide_metabolism
ADA	2.23806	6.95492834224599	1.63578693657617	2.75048607742275e-18	1.63984676261534e-17	Up	leotide_metabolism
AMPD3	0.674274	1.62658663101604	1.27044078794489	1.76468893705831e-05	2.96316751998026e-05	Up	leotide_metabolism
ADCY3	1.059958	2.27395347593583	1.10119563737366	0.000491320561479091	0.000731159508551823	Up	neither
ADCY4	0.791282	1.97796470588235	1.32175283894129	1.86481185392777e-14	7.28903273706514e-14	Up	neither
ADCY6	2.121896	8.63327887700535	2.02455464448099	1.38295794010488e-21	1.252640749595e-20	Up	neither
ADSL	0.439304	0.982752406417112	1.16160835563054	1.63632131595216e-19	1.15030946240816e-18	Up	leotide_metabolism
ADSS2	10.01796	22.5084983957219	1.16788105753428	1.13729747368382e-15	4.80418932829667e-15	Up	leotide_metabolism
CANT1	10.904076	31.2634197860963	1.51960807518156	6.50163297435649e-24	1.02075637697397e-22	Up	leotide_metabolism
ENTPD1	1.014124	2.73651871657754	1.43210765817458	8.92164230230166e-19	5.60279136584544e-18	Up	leotide_metabolism
ENTPD2	0.970178	5.71127299465241	2.55749097590407	4.86802275996167e-13	1.66751906904869e-12	Up	leotide_metabolism
ENTPD6	8.509648	26.2051679144385	1.62267999189234	2.0612775571158e-22	2.1574705097812e-21	Up	leotide_metabolism
FHIT	1.536488	4.76001711229947	1.63133026054308	6.50158043144148e-24	1.02075637697397e-22	Up	neither
GMPS	5.674108	15.317242513369	1.4326910834624	9.12526641527067e-22	8.77142955427038e-21	Up	leotide_metabolism
GUCY2C	0.068438	2.57838021390374	5.23552361250532	8.99640288556116e-05	0.000144617943996563	Up	neither
GUK1	51.852044	120.923968449198	1.22162746472032	1.01906120492972e-23	1.47685485391353e-22	Up	leotide_metabolism
IMPDH1	3.579726	12.3333467914438	1.78464327446829	6.0993693917521e-08	1.27115176261736e-07	Up	leotide_metabolism
IMPDH2	17.54825	54.5173700534759	1.63538880257926	1.13312333770496e-24	2.37200485359572e-23	Up	leotide_metabolism
ITPA	23.337276	62.7557053475936	1.4271104506519	2.36904090539705e-23	3.14315004631552e-22	Up	leotide_metabolism
NPR2	5.100324	12.7956286096257	1.32699022140711	0.000244532717175337	0.000377004614695855	Up	neither
NUDT2	19.164634	48.7370735294118	1.34657317919227	2.46871769984068e-20	1.90617383053272e-19	Up	neither
NUDT5	19.600452	39.416486631016	1.00791226393422	3.81155850405552e-21	3.20579295608955e-20	Up	neither
PDE4A	1.697698	3.80339518716578	1.1637080045701	0.0122920785025121	0.0161719803762101	Up	neither
PDE5A	0.475304	1.35337513368984	1.50963933842494	1.98060286990223e-06	3.60875803374836e-06	Up	neither
PDE6D	5.679152	12.9669320855615	1.19108975469059	4.5503498664193e-23	5.29188836316911e-22	Up	neither
PDE7A	1.020372	2.94155213903743	1.52748239261885	1.56463804083192e-15	6.55061793094964e-15	Up	neither
PPAT	2.148756	4.93154625668449	1.19853839772583	5.92583188844993e-14	2.25997313316592e-13	Up	neither
PFAS	3.045926	8.04741122994652	1.40164386524953	1.61306386856171e-18	9.80326557538794e-18	Up	neither
PRUNE1	8.835832	27.2419545454545	1.62439232360235	7.33622239343458e-23	8.23019521672846e-22	Up	neither
RRM1	8.978166	26.3382390374332	1.55266621485757	2.17556874414082e-20	1.70782146415054e-19	Up	leotide_metabolism
RRM2	0.703672	10.4348430481283	3.89036198317123	2.30314417010805e-26	8.0354141045992e-25	Up	leotide_metabolism
NT5C3A	5.1258	12.0000668449198	1.22719334888128	1.54860607837953e-17	8.14964763035484e-17	Up	leotide_metabolism
CTPS2	4.287336	9.4549385026738	1.14098658845669	9.28828976389202e-18	5.17725973821673e-17	Up	leotide_metabolism
CAD	2.816106	12.6014240641711	2.16181323524061	2.64971810137487e-25	6.93342903193091e-24	Up	neither
DTYMK	7.48542	32.9605342245989	2.1385844539701	2.31974001781264e-28	1.82099591398292e-26	Up	leotide_metabolism
DUT	10.079142	29.6262834224599	1.55550482070816	1.96755672657094e-24	3.6341930126075e-23	Up	leotide_metabolism
DHODH	41.106652	19.0351024064171	-1.110709543	1.32018688128859e-14	5.24732507246351e-14	Down	neither
TK1	6.494558	59.5500489304813	3.19679944484886	2.19940248346707e-26	7.96860438240762e-25	Up	leotide_metabolism
TYMS	0.845956	4.93080748663102	2.5431693941174	1.58182634819053e-17	8.27822455553044e-17	Up	leotide_metabolism
UCKL1	11.893784	29.6142663101604	1.31608456333653	7.42639695776984e-23	8.23019521672846e-22	Up	leotide_metabolism
UCK2	1.41568	9.50843930481283	2.74771336379648	1.88903639850694e-28	1.6626475639806e-26	Up	leotide_metabolism

Supplementary Table S3. The two HCC subtypes.

Id	futime	fustat	Cluster
TCGA-2Y-A9GS	724	1	C1
TCGA-2Y-A9GT	1624	1	C2
TCGA-2Y-A9GU	1939	0	C2
TCGA-2Y-A9GV	2532	1	C2
TCGA-2Y-A9GW	1271	1	C1
TCGA-2Y-A9GX	2442	0	C1
TCGA-2Y-A9GY	757	1	C1
TCGA-2Y-A9GZ	848	1	C2
TCGA-2Y-A9H0	3675	0	C2
TCGA-2Y-A9H1	1229	1	C1
TCGA-2Y-A9H2	1731	0	C1
TCGA-2Y-A9H3	1516	0	C2
TCGA-2Y-A9H4	1452	0	C2
TCGA-2Y-A9H5	555	1	C1
TCGA-2Y-A9H6	357	0	C2
TCGA-2Y-A9H7	1168	0	C1
TCGA-2Y-A9H8	633	1	C1
TCGA-2Y-A9H9	697	0	C2
TCGA-2Y-A9HA	36	1	C1
TCGA-2Y-A9HB	260	0	C2
TCGA-3K-AAZ8	396	0	C2
TCGA-4R-AA8I	262	1	C2
TCGA-5C-A9VG	328	0	C2
TCGA-5C-A9VH	322	0	C2
TCGA-5C-AAPD	20	0	C1
TCGA-5R-AA1C	520	0	C2
TCGA-5R-AA1D	449	0	C1
TCGA-5R-AAAM	46	1	C2
TCGA-BC-4072	1490	1	C1
TCGA-BC-4073	849	0	C1
TCGA-BC-A10Q	1135	1	C1
TCGA-BC-A10R	308	1	C2
TCGA-BC-A10S	1423	1	C2
TCGA-BC-A10T	837	1	C2
TCGA-BC-A10U	837	1	C2
TCGA-BC-A10W	91	1	C1
TCGA-BC-A10X	770	1	C2
TCGA-BC-A10Y	711	1	C2
TCGA-BC-A10Z	34	1	C2
TCGA-BC-A110	2116	1	C2
TCGA-BC-A112	153	1	C1
TCGA-BC-A216	1351	0	C1
TCGA-BC-A217	1397	1	C2
TCGA-BC-A3KF	8	0	C2
TCGA-BC-A3KG	680	0	C2
TCGA-BC-A5W4	547	1	C2
TCGA-BC-A69H	444	0	C1
TCGA-BC-A69I	387	0	C2
TCGA-BC-A8YO	562	0	C1
TCGA-BD-A2L6	1363	0	C2
TCGA-BD-A3EP	409	0	C1
TCGA-BD-A3ER	1115	0	C1
TCGA-BW-A5NO	20	0	C2
TCGA-BW-A5NP	0	0	C1
TCGA-BW-A5NQ	0	0	C1
TCGA-CC-5258	129	1	C1
TCGA-CC-5259	250	0	C1
TCGA-CC-5260	87	1	C1
TCGA-CC-5261	97	1	C1
TCGA-CC-5262	103	1	C1
TCGA-CC-5263	129	1	C1
TCGA-CC-5264	102	1	C2
TCGA-CC-A123	219	0	C1
TCGA-CC-A1HT	101	1	C1
TCGA-CC-A3M9	300	1	C1
TCGA-CC-A3MA	303	1	C1
TCGA-CC-A3MB	315	1	C2
TCGA-CC-A3MC	363	0	C1
TCGA-CC-A5UC	347	1	C1
TCGA-CC-A5UD	304	1	C1
TCGA-CC-A5UE	272	1	C1
TCGA-CC-A7IE	217	1	C1
TCGA-CC-A7IF	649	1	C2
TCGA-CC-A7IG	299	1	C1
TCGA-CC-A7IH	365	0	C2
TCGA-CC-A7II	399	0	C1
TCGA-CC-A7IJ	382	0	C1
TCGA-CC-A7IK	262	1	C2
TCGA-CC-A7IL	278	1	C2
TCGA-CC-A8HS	300	1	C2
TCGA-CC-A8HT	140	1	C1

TCGA-CC-A8HU	344	1	C2
TCGA-CC-A8HV	279	1	C1
TCGA-CC-A9FS	211	0	C2
TCGA-CC-A9FU	0	0	C1
TCGA-CC-A9FV	0	0	C2
TCGA-CC-A9FW	248	0	C1
TCGA-DD-A113	2425	0	C1
TCGA-DD-A114	1149	1	C1
TCGA-DD-A115	2542	1	C2
TCGA-DD-A116	1622	1	C2
TCGA-DD-A118	3437	0	C2
TCGA-DD-A119	223	1	C1
TCGA-DD-A11A	79	0	C2
TCGA-DD-A11B	14	1	C2
TCGA-DD-A11C	662	0	C2
TCGA-DD-A11D	1560	1	C2
TCGA-DD-A1EA	2415	0	C2
TCGA-DD-A1EB	2017	0	C2
TCGA-DD-A1EC	602	0	C1
TCGA-DD-A1ED	2301	0	C2
TCGA-DD-A1EE	349	1	C1
TCGA-DD-A1EF	394	1	C1
TCGA-DD-A1EG	1372	1	C1
TCGA-DD-A1EH	1495	0	C2
TCGA-DD-A1EI	183	0	C1
TCGA-DD-A1EJ	1005	1	C1
TCGA-DD-A1EK	558	1	C1
TCGA-DD-A1EL	415	1	C1
TCGA-DD-A39V	643	1	C1
TCGA-DD-A39W	827	1	C2
TCGA-DD-A39X	1694	1	C1
TCGA-DD-A39Y	171	1	C1
TCGA-DD-A39Z	601	1	C2
TCGA-DD-A3A1	233	1	C1
TCGA-DD-A3A2	2131	1	C2
TCGA-DD-A3A3	535	1	C1
TCGA-DD-A3A4	612	1	C2
TCGA-DD-A3A5	3125	1	C2
TCGA-DD-A3A6	3258	1	C1
TCGA-DD-A3A7	419	1	C1
TCGA-DD-A3A8	11	1	C2
TCGA-DD-A3A9	931	1	C2
TCGA-DD-A4NA	1008	0	C1
TCGA-DD-A4NB	989	0	C2
TCGA-DD-A4ND	2746	0	C1
TCGA-DD-A4NE	660	1	C2
TCGA-DD-A4NF	942	0	C2
TCGA-DD-A4NG	802	1	C1
TCGA-DD-A4NH	917	0	C1
TCGA-DD-A4NI	816	0	C2
TCGA-DD-A4NJ	928	0	C1
TCGA-DD-A4NK	1210	1	C2
TCGA-DD-A4NL	1711	0	C2
TCGA-DD-A4NN	899	1	C2
TCGA-DD-A4NO	2245	0	C2
TCGA-DD-A4NP	3308	0	C2
TCGA-DD-A4NQ	373	1	C1
TCGA-DD-A4NR	9	1	C1
TCGA-DD-A4NS	2456	1	C1
TCGA-DD-A4NV	2398	0	C2
TCGA-DD-A73A	728	0	C2
TCGA-DD-A73B	283	1	C2
TCGA-DD-A73C	701	0	C2
TCGA-DD-A73D	693	0	C2
TCGA-DD-A73E	44	0	C2
TCGA-DD-A73F	1085	0	C2
TCGA-DD-A73G	3478	0	C2
TCGA-DD-AA3A	410	1	C1
TCGA-DD-AAC8	16	1	C1
TCGA-DD-AAC9	347	0	C1
TCGA-DD-AACA	2301	0	C2
TCGA-DD-AACB	2324	0	C1
TCGA-DD-AACC	1685	1	C1
TCGA-DD-AACD	381	1	C2
TCGA-DD-AACE	2184	0	C2
TCGA-DD-AACF	365	1	C2
TCGA-DD-AACG	469	1	C1
TCGA-DD-AACH	195	1	C1
TCGA-DD-AACI	1618	0	C1
TCGA-DD-AACJ	2102	0	C2
TCGA-DD-AACK	9	0	C1
TCGA-DD-AACL	107	1	C1
TCGA-DD-AACN	1302	0	C2

TCGA-DD-AACO	1876	0	C2
TCGA-DD-AACP	415	0	C1
TCGA-DD-AACQ	432	1	C2
TCGA-DD-AACS	1804	0	C2
TCGA-DD-AACT	1562	0	C2
TCGA-DD-AACU	1567	0	C2
TCGA-DD-AACV	1531	0	C2
TCGA-DD-AACW	1424	0	C2
TCGA-DD-AACX	170	0	C2
TCGA-DD-AACY	1450	0	C2
TCGA-DD-AACZ	171	1	C1
TCGA-DD-AAD0	137	0	C2
TCGA-DD-AAD1	564	0	C1
TCGA-DD-AAD2	658	0	C1
TCGA-DD-AAD3	1295	0	C2
TCGA-DD-AAD5	1345	0	C1
TCGA-DD-AAD6	672	0	C1
TCGA-DD-AAD8	1219	0	C2
TCGA-DD-AADA	1233	0	C2
TCGA-DD-AADB	1242	0	C1
TCGA-DD-AADC	425	1	C1
TCGA-DD-AADD	1231	0	C2
TCGA-DD-AADF	115	1	C2
TCGA-DD-AADG	1145	0	C2
TCGA-DD-AADI	1085	0	C1
TCGA-DD-AADJ	1066	0	C2
TCGA-DD-AADK	1049	0	C1
TCGA-DD-AADL	636	0	C2
TCGA-DD-AADM	12	1	C1
TCGA-DD-AADN	898	0	C1
TCGA-DD-AADO	453	0	C1
TCGA-DD-AADP	458	0	C1
TCGA-DD-AADQ	436	0	C2
TCGA-DD-AADR	2028	0	C1
TCGA-DD-AADS	474	0	C2
TCGA-DD-AADU	554	0	C2
TCGA-DD-AADV	574	0	C2
TCGA-DD-AADW	587	0	C1
TCGA-DD-AADY	555	0	C2
TCGA-DD-AAE0	555	0	C1
TCGA-DD-AAE1	552	0	C2
TCGA-DD-AAE2	638	0	C2
TCGA-DD-AAE3	566	0	C2
TCGA-DD-AAE4	608	0	C2
TCGA-DD-AAE6	141	0	C2
TCGA-DD-AAE7	644	0	C2
TCGA-DD-AAE9	722	0	C2
TCGA-DD-AAEA	575	0	C2
TCGA-DD-AAEB	478	0	C2
TCGA-DD-AAED	763	0	C1
TCGA-DD-AAEE	810	0	C2
TCGA-DD-AAEG	719	0	C2
TCGA-DD-AAEH	784	0	C2
TCGA-DD-AAEI	1531	0	C2
TCGA-DD-AAEK	1067	0	C2
TCGA-DD-AAVP	2752	0	C2
TCGA-DD-AAVQ	2728	0	C1
TCGA-DD-AAVR	2513	0	C1
TCGA-DD-AAVS	1823	0	C1
TCGA-DD-AAVU	2202	0	C1
TCGA-DD-AAVV	2455	0	C1
TCGA-DD-AAVW	2317	0	C1
TCGA-DD-AAVX	1718	0	C2
TCGA-DD-AAVY	1970	0	C2
TCGA-DD-AAVZ	1900	0	C2
TCGA-DD-AAW0	2015	0	C1
TCGA-DD-AAW1	1989	0	C2
TCGA-DD-AAW2	1855	0	C2
TCGA-DD-AAW3	1633	0	C2
TCGA-ED-A459	910	0	C1
TCGA-ED-A4XI	819	0	C2
TCGA-ED-A5KG	854	0	C1
TCGA-ED-A627	423	0	C2
TCGA-ED-A66X	406	0	C1
TCGA-ED-A66Y	296	1	C1
TCGA-ED-A7PX	6	0	C1
TCGA-ED-A7PY	390	0	C2
TCGA-ED-A7PZ	6	0	C2
TCGA-ED-A7XO	427	0	C2
TCGA-ED-A7XP	400	0	C1
TCGA-ED-A82E	408	0	C1
TCGA-ED-A8O5	406	0	C2
TCGA-ED-A8O6	56	1	C1

TCGA-ED-A97K	6	0	C1
TCGA-EP-A12J	570	0	C2
TCGA-EP-A26S	608	0	C2
TCGA-EP-A2KA	627	1	C1
TCGA-EP-A2KB	596	1	C2
TCGA-EP-A2KC	19	1	C1
TCGA-EP-A3JL	303	0	C1
TCGA-EP-A3RK	363	0	C1
TCGA-ES-A2HS	688	1	C2
TCGA-ES-A2HT	438	1	C1
TCGA-FV-A23B	1852	1	C1
TCGA-FV-A2QQ	729	0	C1
TCGA-FV-A2QR	581	1	C2
TCGA-FV-A3I0	848	0	C1
TCGA-FV-A3I1	247	1	C1
TCGA-FV-A3R2	194	1	C2
TCGA-FV-A3R3	366	1	C1
TCGA-FV-A495	1	0	C2
TCGA-FV-A496	10	0	C2
TCGA-FV-A4ZP	2486	1	C1
TCGA-FV-A4ZQ	12	0	C1
TCGA-G3-A25S	416	1	C1
TCGA-G3-A25T	1553	0	C1
TCGA-G3-A25U	1636	0	C2
TCGA-G3-A25V	860	0	C1
TCGA-G3-A25X	1779	0	C1
TCGA-G3-A25Y	452	1	C1
TCGA-G3-A25Z	655	0	C2
TCGA-G3-A3CG	673	0	C2
TCGA-G3-A3CH	780	0	C2
TCGA-G3-A3CI	180	0	C2
TCGA-G3-A3CJ	594	0	C2
TCGA-G3-A3CK	585	0	C2
TCGA-G3-A5SI	768	1	C2
TCGA-G3-A5SJ	698	0	C1
TCGA-G3-A5SK	744	0	C2
TCGA-G3-A5SL	621	0	C2
TCGA-G3-A5SM	520	0	C1
TCGA-G3-A6UC	671	0	C2
TCGA-G3-A7M5	447	0	C2
TCGA-G3-A7M6	632	0	C1
TCGA-G3-A7M7	361	0	C1
TCGA-G3-A7M8	430	0	C2
TCGA-G3-A7M9	56	1	C1
TCGA-G3-AAUZ	480	0	C2
TCGA-G3-AAV0	476	0	C2
TCGA-G3-AAV1	359	1	C1
TCGA-G3-AAV2	372	0	C2
TCGA-G3-AAV3	412	0	C1
TCGA-G3-AAV4	27	1	C2
TCGA-G3-AAV5	354	0	C2
TCGA-G3-AAV6	65	1	C1
TCGA-G3-AAV7	361	0	C1
TCGA-GJ-A3OU	879	0	C1
TCGA-GJ-A6C0	31	1	C1
TCGA-GJ-A9DB	67	1	C1
TCGA-HP-A5MZ	91	1	C1
TCGA-HP-A5N0	752	1	C2
TCGA-K7-A5RF	631	0	C2
TCGA-K7-A5RG	519	0	C1
TCGA-K7-A6G5	512	0	C2
TCGA-K7-AAU7	359	0	C1
TCGA-KR-A7K0	65	1	C1
TCGA-KR-A7K2	829	0	C2
TCGA-KR-A7K7	951	0	C1
TCGA-KR-A7K8	906	0	C2
TCGA-LG-A6GG	387	0	C2
TCGA-LG-A9QC	425	0	C2
TCGA-LG-A9QD	366	0	C2
TCGA-MI-A75C	291	0	C1
TCGA-MI-A75E	507	0	C2
TCGA-MI-A75G	698	0	C2
TCGA-MI-A75H	747	0	C2
TCGA-MI-A75I	630	0	C1
TCGA-MR-A520	229	0	C2
TCGA-MR-A8JO	330	0	C1
TCGA-NI-A4U2	1791	1	C2
TCGA-NI-A8LF	799	0	C2
TCGA-O8-A75V	538	0	C1
TCGA-PD-A5DF	639	1	C1
TCGA-QA-A7B7	94	0	C2
TCGA-RC-A6M3	0	0	C1
TCGA-RC-A6M4	22	0	C2

TCGA-RC-A6M5	15	0	C2
TCGA-RC-A6M6	9	0	C1
TCGA-RC-A7S9	640	0	C2
TCGA-RC-A7SB	588	0	C2
TCGA-RC-A7SF	579	0	C2
TCGA-RC-A7SH	468	0	C2
TCGA-RC-A7SK	472	0	C2
TCGA-RG-A7D4	1098	0	C1
TCGA-T1-A6J8	23	0	C2
TCGA-UB-A7MA	848	0	C1
TCGA-UB-A7MB	601	0	C2
TCGA-UB-A7MC	500	0	C2
TCGA-UB-A7MD	52	1	C1
TCGA-UB-A7ME	486	0	C1
TCGA-UB-A7MF	214	1	C2
TCGA-UB-AA0U	327	0	C2
TCGA-UB-AA0V	314	0	C2
TCGA-WJ-A86L	345	0	C2
TCGA-WQ-A9G7	30	0	C1
TCGA-WQ-AB4B	395	0	C2
TCGA-WX-AA44	615	0	C1
TCGA-WX-AA46	756	0	C2
TCGA-WX-AA47	556	1	C2
TCGA-XR-A8TC	1339	0	C2
TCGA-XR-A8TD	1030	0	C1
TCGA-XR-A8TE	925	0	C2
TCGA-XR-A8TF	693	1	C2
TCGA-XR-A8TG	898	0	C1
TCGA-YA-A8S7	412	1	C1
TCGA-ZP-A9CV	1088	1	C2
TCGA-ZP-A9CY	782	0	C2
TCGA-ZP-A9CZ	706	0	C2
TCGA-ZP-A9D0	1091	0	C2
TCGA-ZP-A9D1	21	0	C1
TCGA-ZP-A9D2	765	1	C1
TCGA-ZP-A9D4	395	0	C2
TCGA-ZS-A9CD	1386	1	C1
TCGA-ZS-A9CE	1241	0	C2
TCGA-ZS-A9CF	2412	0	C2
TCGA-ZS-A9CG	341	0	C2

Supplementary Table S4. The results of HCC samples from the TCGA database that underwent LASSO regression analysis.

id	futime	fustat	HSP90AA1	FABP6	MAPT	DCK	NDRG1	HDAC1	PRDX1	RAC3	SEMA3F	BTC	CHGA	CSPG5	GAL	STC1	UCN	GHR	GLP1R	DTYMK	UCK2
**TCGA-G3-A25X**	4.873973	0	9.409763	2.737952	1.281519	3.71466	8.929766	7.210282	9.416768	3.093527	4.495233	2.297399	1.234017	2.65692	1.306266	4.36522	2.257704	4.743646	1.627795	6.622493	5.109514
**TCGA-DD-AADS**	1.29863	0	7.909564	1.215402	1.372748	1.65933	4.354362	5.299621	7.661542	3.812261	2.884276	1.389025	1.370612	1.392133	1.254358	1.954272	3.195624	3.41247	1.380059	4.487747	2.570814
**TCGA-G3-AAV3**	1.128767	0	9.344611	1.215402	3.096314	4.296937	5.704068	6.885689	9.993957	3.779225	4.237276	1.272327	1.583814	2.741465	1.396523	3.135438	3.159439	4.613829	1.399333	5.006217	3.627432
**TCGA-2Y-A9HB**	0.712329	0	9.096795	1.274253	1.650103	3.700578	4.808026	7.077246	9.369352	2.820465	3.619348	2.138761	1.253507	1.359049	1.254358	2.527547	2.158067	5.653	1.39865	4.172195	4.117275
**TCGA-DD-A73E**	0.120548	0	9.686737	1.215402	3.912204	4.322273	6.9643	6.844208	9.790846	4.530901	5.539281	1.956301	1.241512	2.949235	1.254358	3.772045	3.893686	5.393782	1.406705	4.844284	3.444277
**TCGA-DD-AAEG**	1.969863	0	9.134447	1.314019	2.890903	3.901518	7.555092	6.868026	10.15879	3.201183	3.251799	2.186742	1.352014	2.410206	1.544893	2.137698	3.0152	6.235383	1.44954	4.255272	3.914026
**TCGA-ED-A8O6**	0.153425	1	9.145738	1.726671	1.550439	3.990494	11.24197	7.468652	9.816581	5.803519	4.938241	1.315811	3.177968	1.807854	3.289903	3.710817	2.098998	1.869739	2.841349	5.376507	4.769458
**TCGA-G3-AAV7**	0.989041	0	9.963573	3.980499	2.93566	5.910972	10.23447	8.342488	10.72877	4.492261	3.564035	3.317779	1.353116	2.181876	1.319028	6.031657	2.048599	4.9245	1.417955	5.669225	5.262613
**TCGA-CC-A7IJ**	1.046575	0	11.02695	1.794819	1.462929	4.973657	7.079595	8.49314	10.39624	4.619424	4.344477	2.62515	1.733717	2.951858	1.254358	5.611743	3.572608	1.982414	1.380059	6.485943	4.731374
**TCGA-2Y-A9H9**	1.909589	0	8.870508	1.268681	1.759415	3.552019	5.037802	6.814314	9.256367	4.452133	4.028244	2.156334	1.305975	1.463238	1.254358	4.10877	3.252593	4.546797	1.421532	4.855791	3.275611
**TCGA-CC-5261**	0.265753	1	10.50076	1.330829	2.653866	6.446862	7.436658	7.557932	8.948373	2.965371	4.756937	3.949684	1.29575	2.202763	1.347807	4.22172	1.884467	5.943558	2.458864	5.114653	4.967712
**TCGA-RC-A7SH**	1.282192	0	8.407089	1.404506	2.318704	3.243202	5.795068	6.472636	7.939136	5.29232	3.889693	1.510014	1.210141	1.690487	1.307827	2.850693	2.723747	4.318761	1.380059	5.411087	4.224466
**TCGA-DD-A1EI**	0.50137	0	10.07078	1.215402	2.251952	3.997459	6.284369	7.013666	10.01642	3.858943	4.213024	2.301205	1.282179	1.670304	1.603508	4.489831	3.040137	6.145724	2.710575	5.529364	4.556514
**TCGA-G3-A3CJ**	1.627397	0	9.016811	1.215402	1.478284	3.23882	5.789111	6.205672	8.36701	5.340397	3.035137	1.089147	1.246804	1.319955	1.254358	3.866509	2.404642	2.705966	1.380059	4.138887	3.47586
**TCGA-ED-A7PY**	1.068493	0	10.22479	1.215402	1.439273	3.013914	7.545733	7.124798	7.839686	2.796213	3.27812	1.542802	1.241753	1.685023	1.448138	2.175616	2.178359	4.694481	2.743649	4.599589	4.03952
**TCGA-K7-A5RG**	1.421918	0	9.19504	1.701341	1.631054	4.434749	6.673884	7.132873	9.961966	4.770236	3.510494	2.30681	1.448051	1.786545	1.586169	3.366578	2.686306	5.329814	2.002201	4.705082	2.990634
**TCGA-G3-A7M5**	1.224658	0	8.294707	1.271702	1.367262	2.571776	5.137249	5.897284	8.476039	5.808341	3.145283	1.484048	1.440481	1.65195	1.299776	4.295728	3.386383	6.421831	1.380059	4.504119	2.410291
**TCGA-DD-AADN**	2.460274	0	8.810789	1.309302	2.795209	2.8283	7.497936	7.001295	9.337347	5.668855	4.387826	1.143491	1.27961	1.281737	1.330232	1.346003	3.506583	2.105077	1.380059	4.766389	4.133857
**TCGA-CC-A8HS**	0.821918	1	9.698237	1.289104	1.426399	4.604756	7.617079	7.22109	8.39478	5.136394	3.688541	2.683495	1.973901	2.098282	1.822901	3.522062	1.896844	6.510217	6.230419	5.905389	5.450209
**TCGA-WQ-AB4B**	1.082192	0	9.109773	1.28773	1.59177	3.056295	5.678864	6.104495	9.269144	3.514428	3.197818	1.788304	1.289273	1.272513	1.254358	2.31632	2.613292	4.399585	1.414142	4.03294	3.344142
**TCGA-FV-A3R3**	1.00274	1	9.484945	1.215402	2.10268	4.457789	6.895492	7.658911	9.27121	1.616784	4.404123	1.891245	1.310884	1.338148	2.788602	5.480723	1.551329	6.49253	3.315089	3.872379	2.717426
**TCGA-G3-AAV0**	1.30411	0	7.865013	1.280722	1.528811	2.838426	4.391855	6.120548	8.560288	5.358484	2.91899	1.925567	1.371047	1.378887	1.254358	2.083636	1.908312	7.251258	1.380059	4.281242	3.299791
**TCGA-5C-A9VG**	0.89863	0	10.36627	1.482198	2.112852	4.064226	5.750764	8.668209	9.441957	3.621939	4.68327	1.398718	1.289389	1.785897	2.556653	2.843499	2.447308	2.607748	1.44736	5.212622	6.219663
**TCGA-BC-4072**	4.082192	1	10.20127	1.933961	1.628096	5.135291	7.107874	7.939093	9.421065	3.882541	3.974874	2.437567	1.289041	3.293607	4.691019	3.701314	2.484435	5.519358	2.817219	5.43411	3.905081
**TCGA-DD-AADB**	3.40274	0	9.66043	1.215402	1.78572	4.147025	7.848974	7.310735	8.980336	4.13628	4.365395	1.484293	2.108178	1.924182	1.405166	3.665716	1.987106	3.731888	4.062437	5.781615	4.784113
**TCGA-CC-5258**	0.353425	1	10.34974	1.215402	1.286713	5.431922	8.573816	8.034325	9.894189	4.429156	4.494834	4.028567	1.85035	1.569757	3.581841	3.757479	1.345546	3.90228	2.434478	5.572519	5.581514
**TCGA-DD-A73C**	1.920548	0	9.710457	1.215402	2.977105	3.908947	5.347594	6.914562	9.602872	4.983785	4.549476	1.570688	1.235229	1.311981	1.254358	2.682157	2.551092	8.217953	1.502353	4.135185	3.523014
**TCGA-DD-AAEE**	2.219178	0	9.144361	1.545717	2.432832	4.671826	6.605569	6.476584	10.22512	3.793001	4.305225	1.941861	1.229765	2.209258	1.254358	2.40347	2.43611	4.185845	1.405008	5.330232	3.853787
**TCGA-ED-A82E**	1.117808	0	9.210486	1.273906	2.570141	5.14549	5.567948	6.69766	8.209285	3.238474	4.213496	3.775877	1.253268	1.370585	1.346838	2.932621	3.437141	7.585855	1.443314	4.239295	4.836783
**TCGA-QA-A7B7**	0.257534	0	8.921575	3.922849	2.061181	4.972974	5.550789	7.333785	9.63661	6.298953	4.112933	3.374245	1.246564	2.224693	1.441499	2.146226	2.821277	8.172859	1.387899	5.827045	4.870744
**TCGA-DD-AAE6**	0.386301	0	8.208498	1.423298	2.316773	3.352675	7.370454	7.427705	9.277372	3.956871	3.500755	1.23506	1.754746	2.529315	1.254358	2.437081	2.599768	4.235984	1.380059	6.400854	4.477857
**TCGA-DD-A3A2**	5.838356	1	8.177214	1.215402	1.401808	2.948673	4.481952	5.676328	8.302018	4.245633	3.014821	1.639754	1.225621	1.342628	1.254358	1.476846	2.394486	6.783345	1.393173	3.40617	2.485099
**TCGA-DD-A39Y**	0.468493	1	10.56305	1.664935	3.64631	4.092529	9.907906	7.962778	11.19992	6.320173	3.126022	1.942851	1.245844	1.881359	1.367771	2.14459	3.31874	3.756388	1.425099	6.363019	5.320344
**TCGA-BC-A112**	0.419178	1	9.705855	6.812026	2.431795	4.737374	8.453658	6.956346	10.84207	2.313049	4.312645	2.2374	1.407622	2.521884	1.254358	4.398561	2.672351	3.898257	1.467378	5.089118	5.184527
**TCGA-ZP-A9CZ**	1.934247	0	9.448398	1.287272	2.336386	5.526397	6.769488	6.641064	9.471279	4.707818	3.976877	2.638115	1.288809	1.567172	1.36756	2.942918	1.927183	6.394185	1.425099	5.42449	4.775573
**TCGA-CC-A3MB**	0.863014	1	9.912439	1.215402	4.553198	4.538545	7.195794	7.180453	9.548065	3.605352	4.193291	2.890799	1.63387	1.561463	1.254358	3.700983	1.502819	6.110596	2.557858	6.00855	5.056425
**TCGA-DD-AAE1**	1.512329	0	9.620283	1.215402	2.397169	3.11732	8.258052	6.484127	8.321869	2.714117	4.83551	2.557483	1.210141	1.397311	1.254358	3.040672	2.299419	4.246626	2.129644	4.208105	5.206662
**TCGA-EP-A3RK**	0.994521	0	9.470952	1.215402	2.508572	4.961862	5.699946	6.349881	10.177	3.734755	4.22474	1.593475	1.386686	2.464499	1.254358	3.701579	2.699148	4.011474	1.409753	5.617489	4.633649
**TCGA-G3-A3CG**	1.843836	0	9.016853	1.215402	1.817824	4.002275	4.523866	7.005727	8.994351	4.15789	3.578247	2.966166	1.377326	1.282065	1.254358	4.395182	2.460165	6.15988	1.405008	4.102102	3.623007
**TCGA-DD-AA3A**	1.123288	1	9.781487	2.267247	2.341593	5.110962	6.382097	7.739485	9.398288	3.136039	3.935097	2.963995	1.354327	2.47704	3.419213	5.338018	3.49449	3.065524	4.215201	6.688213	5.757951
**TCGA-DD-A4NA**	2.761644	0	10.05612	1.215402	1.517995	5.732491	7.256143	6.924044	7.965184	3.292139	4.099103	2.396921	1.235229	1.494254	1.709424	4.695046	3.961712	6.782435	1.813734	4.212055	5.341744
**TCGA-DD-A118**	9.416438	0	10.0526	1.622283	2.967311	4.215416	7.174747	6.70308	8.185189	1.45283	4.440896	1.230202	2.043492	2.053683	1.448928	4.498284	1.268229	8.133071	1.591078	4.63399	4.587326
**TCGA-CC-A9FV**	0.00274	0	9.942902	1.427696	5.48324	4.754091	4.805772	6.450378	8.81152	3.81873	3.508836	2.839399	1.238133	1.746036	1.254358	2.455386	2.536642	2.682161	1.380059	4.318133	2.415225
**TCGA-ED-A459**	2.493151	0	9.510187	4.378348	1.562995	4.270329	7.911021	7.545389	10.11337	3.82245	3.841182	2.344684	1.30357	2.75118	1.254358	2.842517	2.196907	5.57831	1.40047	5.202922	4.52529
**TCGA-DD-AAEA**	1.575342	0	8.341954	1.215402	1.765422	3.832439	4.830071	6.257058	8.879145	5.354448	3.556109	2.106006	1.360363	1.684084	1.417708	3.016242	2.908535	5.472169	1.429763	5.286451	3.675795
**TCGA-DD-AADP**	1.254795	0	9.44108	1.215402	1.502459	3.645747	7.155093	6.479009	8.243067	3.691437	3.950436	1.770141	1.263257	1.631595	1.254358	3.679408	2.099189	6.558314	1.402855	3.873097	4.098641
**TCGA-DD-AACT**	4.279452	0	8.866206	1.493469	2.317367	3.7734	5.49908	6.137446	9.027095	3.591705	3.921565	1.781376	1.28416	1.69825	1.254358	2.003785	2.05267	4.704236	1.380059	4.310272	3.047474
**TCGA-DD-A73D**	1.89863	0	8.635738	1.215402	2.131737	4.11622	6.421461	6.409671	9.195949	4.83902	3.766505	1.133727	1.210141	1.260327	1.254358	3.315349	2.843301	4.658237	1.380059	4.797	3.460363
**TCGA-DD-A4NB**	2.709589	0	9.329346	1.215402	2.312603	4.528916	5.677651	7.035541	9.677316	3.492829	4.021549	1.253495	1.210141	1.228167	1.442393	3.679004	1.874034	5.736354	1.389851	4.268248	3.326131
**TCGA-2Y-A9GT**	4.449315	1	8.938335	1.215402	1.379491	3.94243	5.614401	6.463055	9.299626	4.337582	3.829342	1.87393	1.210141	1.282612	1.254358	3.270841	3.043641	7.699308	1.545562	3.890394	3.291025
**TCGA-DD-AADL**	1.742466	0	8.368305	1.370421	2.225488	4.282623	6.993231	7.047244	9.416156	5.329844	3.655352	2.277315	1.288229	2.989216	1.297755	2.148791	1.933827	6.701287	1.405347	5.125001	4.070691
**TCGA-DD-A4ND**	7.523288	0	10.06203	1.215402	1.683345	4.268298	7.160359	7.307398	8.459408	3.127134	4.483793	2.392781	1.351463	1.588297	1.500309	3.997487	2.040063	7.108186	3.25048	4.549298	3.726958
**TCGA-2Y-A9H6**	0.978082	0	9.736208	1.343587	1.932456	3.965569	5.924447	6.450573	8.193562	2.285917	4.174402	2.314947	1.327973	1.59683	1.453461	3.523026	2.033785	7.014212	1.760356	4.655628	3.573948
**TCGA-DD-AAW1**	5.449315	0	9.778331	1.215402	1.605774	4.027651	6.171671	6.123471	9.327745	4.167501	3.496484	1.212731	1.210141	1.52037	1.254358	4.653817	3.078366	4.569067	1.388588	3.435985	2.927182
**TCGA-DD-A1EA**	6.616438	0	9.429983	1.215402	1.712135	4.611102	6.472148	6.752069	9.425573	4.7158	4.038822	1.876203	1.210141	1.911482	1.254358	4.144835	2.801118	6.058889	1.391799	3.856547	3.612044
**TCGA-ED-A66Y**	0.810959	1	10.01571	1.215402	1.578692	5.224327	7.187652	7.554728	8.371558	2.53112	4.582429	2.331133	1.210141	1.843557	1.254358	4.792142	1.440471	3.073779	4.030745	5.110354	4.475663
**TCGA-DD-AACH**	0.534247	1	9.855498	1.437255	3.598118	5.747507	10.01724	8.30829	10.14488	4.766375	4.401883	3.916351	1.296096	4.113727	1.254358	3.843557	3.873361	4.96232	1.846969	6.282169	6.243244
**TCGA-DD-A3A4**	1.676712	1	9.490199	1.215402	2.536085	3.334623	3.491011	7.673511	9.766377	4.127605	3.45552	1.623302	1.232682	1.351744	1.254358	2.179031	2.855074	4.492634	1.445721	4.233332	3.00514
**TCGA-DD-A11A**	0.216438	0	9.749902	1.270309	1.898386	3.778987	6.366329	6.818327	9.269938	5.814024	3.982983	1.621274	1.48542	1.260327	1.254358	4.686368	4.001087	4.984978	1.388703	4.429097	3.748654
**TCGA-ZP-A9D0**	2.989041	0	9.426297	1.497621	1.96494	4.384555	5.739523	6.961296	8.975301	1.724052	3.305879	2.410652	1.233167	1.482184	1.398175	2.499362	1.953865	7.446448	1.850672	4.051876	3.332909
**TCGA-UB-AA0V**	0.860274	0	8.858187	1.215402	1.40055	3.768348	5.906333	6.39036	8.220012	4.068497	3.258803	2.109395	1.210141	1.43603	1.322444	2.335411	2.037104	7.501364	1.380059	3.545071	2.659542
**TCGA-MR-A520**	0.627397	0	8.55085	1.215402	1.531642	3.156446	5.205828	5.922245	8.993068	3.448385	3.185528	1.987779	1.230981	1.222435	1.707211	3.680714	2.151879	6.921861	1.380059	3.70974	2.395174
**TCGA-EP-A2KA**	1.717808	1	9.110994	1.326177	2.179622	4.629437	7.243388	8.063117	8.849516	2.684159	3.825847	3.77713	1.272225	1.181292	1.254358	2.800554	2.399575	4.355902	1.607731	5.120128	3.92584
**TCGA-DD-AAE7**	1.764384	0	9.403715	1.215402	1.69914	3.306436	6.016183	5.772515	8.400701	3.223085	3.284526	1.831692	1.210141	1.567862	1.333179	2.694851	2.542377	5.959052	1.380059	3.893869	2.56544
**TCGA-DD-AAE4**	1.665753	0	8.664595	1.215402	1.29154	3.26039	4.559204	6.108782	8.546758	4.291234	3.584136	1.382815	1.210141	1.389835	1.254358	2.941326	1.798517	5.875568	1.380059	5.141388	2.715487
**TCGA-DD-A4NF**	2.580822	0	10.37676	1.215402	1.618918	4.252548	6.621402	6.863259	9.273526	3.20846	4.160784	1.143054	1.233532	2.633854	1.254358	4.449996	2.515037	5.193491	1.429097	3.89923	4.500603

**TCGA-BW-A5NP**	0.00274	0	10.325	1.215402	2.804884	5.005946	7.372961	7.722997	8.801705	3.187653	3.845002	1.141632	1.36266	2.2086	1.525545	4.017369	2.256159	3.855773	4.150306	5.470849	5.171465
**TCGA-RC-A6M3**	0.00274	0	10.83543	1.320504	1.356759	3.310138	6.306293	8.13876	9.573357	3.663113	3.961055	1.854576	1.413404	2.551411	6.655542	4.145214	3.492297	4.344454	1.843409	4.655666	4.919819
**TCGA-DD-AAE0**	1.520548	0	9.009184	1.858347	2.730066	3.836686	6.985909	7.848776	9.265187	5.452181	3.826342	1.677515	1.210141	1.801692	2.482971	3.89917	1.681177	2.169756	2.407236	5.943802	5.61497
**TCGA-XR-A8TF**	1.89863	1	8.988726	1.215402	1.645218	3.953205	5.650822	6.518284	9.200646	4.093477	3.935217	1.06929	1.257678	1.884479	1.254358	4.703616	2.698225	5.894854	1.40047	3.951405	4.525557
**TCGA-G3-A6UC**	1.838356	0	8.99865	1.278878	1.99742	4.304563	5.980454	5.957524	9.237522	5.179814	5.103411	2.790892	2.008084	1.936242	1.254358	3.652613	3.59655	6.197464	1.476886	3.919056	3.604903
**TCGA-DD-A1ED**	6.30411	0	8.850578	1.215402	1.358606	3.438659	6.256561	6.249671	8.548555	3.429937	3.499919	1.551608	1.273988	1.282721	1.482121	5.774979	1.865598	6.191312	1.414255	3.357383	2.468712
**TCGA-CC-5262**	0.282192	1	9.500885	1.893927	2.540012	4.463943	5.935382	7.085042	9.486148	3.615503	4.345831	2.618305	1.235471	2.243627	1.254358	4.397321	2.655662	4.996844	1.432972	5.544899	4.809655
**TCGA-DD-AAW0**	5.520548	0	9.855328	1.215402	2.432697	4.122011	5.748732	5.821474	8.697104	4.292986	3.65558	1.820752	1.233895	1.608641	2.110472	2.174841	1.934867	6.265328	1.410091	4.276978	3.673526
**TCGA-DD-AAD8**	3.339726	0	8.948472	1.572202	1.822141	4.106681	4.052216	6.979771	8.940567	5.193488	3.650462	1.761049	1.406146	1.939919	1.254358	3.705198	4.308247	5.671036	1.380059	5.62249	3.791524
**TCGA-RC-A7SK**	1.293151	0	8.881384	1.492599	2.478255	3.483875	5.676209	6.418829	9.516813	3.913863	3.365737	1.893255	5.93107	2.272297	1.480873	1.216399	2.59864	5.078284	1.516422	4.707142	3.970567
**TCGA-DD-AAC8**	0.043836	1	10.06687	1.705491	3.936666	4.731932	7.53977	7.118544	10.13684	4.313525	4.329849	1.098496	1.365607	1.746334	4.395	4.071127	3.700471	1.52587	1.391685	4.727882	5.456276
**TCGA-GJ-A3OU**	2.408219	0	9.842932	1.575551	2.894398	5.090559	7.050303	6.549136	9.58889	4.437524	4.626645	1.903083	1.210141	1.7075	3.694586	3.527726	2.745855	4.836352	1.380059	5.132527	4.572259
**TCGA-KR-A7K8**	2.482192	0	8.879334	1.215402	2.330765	4.010045	4.423982	6.099211	9.030585	5.927491	4.666987	1.781566	1.325047	1.95013	1.254358	3.409092	2.733676	5.589659	1.390195	4.874568	3.410883
**TCGA-CC-A1HT**	0.276712	1	9.97693	5.318995	3.440625	5.69167	8.904216	7.942687	10.91337	4.706837	4.372715	3.63717	1.323131	1.825942	1.426498	4.909938	3.400626	1.896929	1.435952	6.420546	5.509584
**TCGA-BC-A69H**	1.216438	0	10.10636	2.322375	2.029497	2.966405	10.00775	7.266304	9.038994	3.18932	4.361796	1.498071	2.30198	1.399891	1.458565	3.099669	3.987502	2.249716	1.568397	7.11707	4.736827
**TCGA-DD-A4NN**	2.463014	1	11.13017	1.277492	2.139553	4.165997	6.029965	7.032306	8.605135	3.671138	4.82217	1.553376	1.255892	1.817983	2.092981	3.456869	1.164453	4.162585	2.542459	5.748638	4.242611
**TCGA-DD-AACC**	4.616438	1	9.815001	1.215402	1.474933	3.844723	5.617832	6.484205	8.882899	3.269079	3.284026	2.010828	1.26302	1.398105	1.254358	2.697989	1.878402	4.814277	1.446704	4.890678	3.390082
**TCGA-DD-AADI**	2.972603	0	10.20513	1.215402	2.563883	3.686058	6.830376	7.354245	8.523787	4.059735	3.446125	1.465637	1.239099	1.648971	1.31704	2.09152	2.195808	4.567655	1.380059	4.695525	3.667817
**TCGA-DD-AAEB**	1.309589	0	7.768963	1.215402	1.305115	2.783127	4.820578	5.719475	7.474238	4.419705	3.136713	1.06929	1.233046	1.627663	1.254358	3.176675	3.152833	4.124301	1.380059	3.442939	2.567923
**TCGA-2Y-A9H1**	3.367123	1	8.278693	1.215402	1.586105	2.241634	5.399805	6.246276	8.610358	4.697657	3.055576	1.55943	1.235713	1.167058	1.362467	2.400016	3.384907	2.593824	1.390883	4.63326	3.48346
**TCGA-DD-AACB**	6.367123	0	9.427023	1.215402	2.92369	3.899288	7.675703	7.663791	10.62367	4.129741	3.720831	1.109459	1.916325	1.664589	1.671701	2.97997	3.362066	3.067372	1.396029	5.517097	4.143367
**TCGA-WX-AA46**	2.071233	0	9.210412	1.259219	1.34956	3.173673	5.622942	6.093134	8.79487	4.503335	3.571284	1.650971	1.210141	1.360074	1.254358	1.166578	2.053215	6.638919	1.380059	3.386172	2.343089
**TCGA-DD-A1EJ**	2.753425	1	10.2061	1.589036	1.364131	4.617468	8.790509	6.981701	9.666707	5.350787	5.43956	1.06929	3.783464	2.035288	1.322554	3.196419	1.664481	3.721506	2.264639	5.483481	4.200043
**TCGA-EP-A2KB**	1.632877	1	8.92339	1.215402	2.47743	3.487067	8.44818	6.832375	8.478772	4.411033	3.559681	1.266444	1.243078	1.324819	1.254358	4.539854	2.186717	4.710311	1.895764	4.934122	4.582233
**TCGA-DD-AACW**	3.90137	0	9.822091	1.215402	1.855123	3.643972	7.957483	6.887408	7.990705	3.455807	3.882593	1.327996	1.539817	1.320484	1.254358	1.616262	2.208708	4.578045	2.549637	5.402596	5.563013
**TCGA-ED-A97K**	0.016438	0	9.555754	1.215402	1.402542	5.321699	7.320825	7.84812	9.034976	2.873987	4.038527	3.088727	1.210141	1.842322	3.326786	3.550992	2.154567	5.833485	3.501547	4.753438	4.795381
**TCGA-DD-AADW**	1.608219	0	9.626909	1.435633	2.359332	5.539659	6.459266	7.318913	8.683654	4.129769	4.760958	2.370029	1.468283	2.261832	1.391348	4.796891	1.957628	6.667002	2.140426	5.979264	4.883783
**TCGA-DD-A39W**	2.265753	1	10.26391	1.215402	3.242669	4.14549	6.601123	7.637344	8.930543	3.619395	3.844319	1.095198	1.234259	1.72238	1.254358	3.172863	1.903322	4.725351	1.380059	4.597721	4.636172
**TCGA-DD-A4NR**	0.024658	1	9.96021	2.350177	2.320187	4.705252	5.954605	7.258282	9.861398	2.360296	4.37501	2.818461	1.446418	1.499468	1.753823	3.760799	3.026035	3.813795	1.39008	5.439878	4.707556
**TCGA-DD-AACU**	4.293151	0	10.23955	2.770995	2.769075	3.948156	7.273458	7.566421	10.53854	2.966814	4.256188	2.995724	1.238616	2.584017	1.254358	2.001634	3.222423	6.19431	1.404215	4.394384	3.988352
**TCGA-DD-AACQ**	1.183562	1	8.474799	1.215402	2.050875	4.012801	6.068093	6.754409	9.335672	5.279758	3.546982	1.127762	1.492229	2.110264	1.254358	2.573498	3.162806	6.364126	1.380059	5.296889	4.231722
**TCGA-DD-A11B**	0.038356	1	9.428548	1.270657	1.593563	4.68264	5.193578	5.843317	7.888637	3.251519	3.653728	2.698103	1.210141	1.777016	1.254358	3.796305	1.544746	7.052452	1.406026	4.1834	4.177375
**TCGA-DD-A4NS**	6.728767	1	9.298434	1.215402	2.483973	4.71527	6.603736	7.278142	8.642511	4.271786	4.630087	2.527585	1.372999	1.409077	1.358739	3.779469	3.211044	6.759702	1.461355	4.281285	3.249712
**TCGA-DD-A1EH**	4.09589	0	10.29647	1.215402	2.244404	5.198727	7.080647	6.720003	8.289903	3.323565	4.459585	1.851606	1.210141	1.348644	1.383751	5.141547	2.885105	6.736481	2.260755	4.639218	3.758551
**TCGA-DD-AAW3**	4.473973	0	8.167372	1.215402	2.012388	4.771441	6.361267	7.188172	9.135105	3.723634	3.754958	2.100496	1.210141	1.312941	1.254358	2.977379	2.474313	4.874964	1.380059	4.363269	3.796915
**TCGA-DD-AADA**	3.378082	0	9.406762	1.323844	1.717812	3.492599	4.615708	5.883739	8.064666	1.470055	3.500337	1.413973	1.210141	1.691655	1.342086	2.288968	2.143135	5.4009	1.380059	3.543654	2.816911
**TCGA-UB-A7MF**	0.586301	1	8.790941	1.265068	2.676111	4.97361	8.026266	7.950017	9.534907	2.789321	4.266807	1.06929	1.813871	1.454553	1.332961	4.089295	3.725582	4.726566	1.380059	5.118454	4.068846
**TCGA-DD-A119**	0.610959	1	9.208193	1.215402	2.171278	3.981872	6.626183	6.133339	9.159914	4.605857	3.246409	1.490554	1.583906	1.604131	4.121246	2.745163	1.835833	2.665133	1.387784	4.561749	3.42457
**TCGA-2Y-A9H8**	1.734247	1	10.36148	3.368943	3.669858	3.954811	6.917481	7.268532	8.967874	5.578062	4.208805	1.664319	1.835455	3.4712	1.394456	2.38568	2.476782	5.909812	4.499618	5.299207	2.953532
**TCGA-K7-A6G5**	1.40274	0	9.275768	1.215402	2.059103	3.590049	5.681567	6.772959	9.635548	4.587187	3.802642	1.53993	1.371915	1.581751	1.254358	3.376587	2.595058	4.860427	1.398195	4.900792	3.247691
**TCGA-WJ-A86L**	0.945205	0	8.668601	3.617547	2.408851	4.23013	5.656484	5.83243	8.059456	5.525156	4.183543	2.006515	1.269636	2.481084	1.340354	2.689212	2.731321	6.964913	1.380059	4.77981	4.130613
**TCGA-ZS-A9CG**	0.934247	0	10.35297	1.215402	1.683512	3.77677	7.666839	6.296866	9.263953	4.166487	3.802328	1.143818	1.210141	1.526455	1.254358	6.121711	3.344064	5.862357	1.410092	3.992899	3.411107
**TCGA-O8-A75V**	1.473973	0	10.054	1.264485	2.956969	2.970345	7.547153	6.70005	10.09455	3.768471	4.056644	1.560041	1.298631	1.451897	1.293927	1.947543	3.642895	4.008371	1.380059	4.549975	4.082028
**TCGA-ED-A7PZ**	0.016438	0	8.228274	1.634832	1.936387	3.081405	7.928617	7.420366	9.956901	4.100393	4.723385	1.803464	1.315434	1.250601	1.332197	2.594095	3.991218	2.550729	1.403082	5.206319	3.728864
**TCGA-G3-A5SL**	1.70137	0	9.063753	1.215402	2.207196	4.453181	6.507717	6.433563	8.805559	4.68793	3.143756	1.130417	1.303914	1.29165	1.254358	4.529561	1.842886	5.110065	1.467378	4.426256	3.282559
**TCGA-DD-A1EE**	0.956164	1	10.29117	2.592	2.770613	4.900775	8.730601	7.34249	9.855828	4.033694	4.505054	1.620549	1.30586	1.553723	1.254358	5.334113	2.94911	3.89592	1.380059	4.449473	5.664952
**TCGA-CC-5263**	0.353425	1	11.27277	1.676721	3.916932	6.519469	10.83227	8.104241	8.365549	4.982593	4.052316	3.72701	5.32812	2.853434	1.751312	5.370804	3.088998	7.05993	4.936797	6.243112	3.775893
**TCGA-DD-AAVS**	4.994521	0	10.7941	1.215402	2.09765	3.676275	7.858206	7.105097	9.113363	2.840623	4.293355	2.143168	1.254343	1.726998	2.515714	3.036233	2.345904	5.480104	3.407878	5.411569	4.635884
**TCGA-DD-A4NO**	6.150685	0	9.513394	1.215402	2.741459	3.743405	6.458339	6.678778	9.55228	3.761046	3.683289	2.048392	1.61886	1.468026	1.254358	4.868195	1.78445	5.057994	1.4648	3.963544	4.915555
**TCGA-CC-A3MA**	0.830137	1	9.254815	1.397723	3.617114	5.717374	7.526677	7.064204	9.697482	3.219128	5.088019	3.102864	1.210141	1.388034	1.402498	3.532378	3.203605	3.40047	1.634407	5.776392	6.140738
**TCGA-NI-A8LF**	2.189041	0	7.46114	1.215402	1.411933	3.449203	6.038266	5.830378	9.037817	4.788041	4.579935	1.525226	1.210141	1.33061	1.289521	5.937382	1.860834	5.812187	1.433856	3.555455	2.720967
**TCGA-DD-A4NE**	1.808219	1	9.249215	3.881143	1.636082	5.465704	7.220819	7.015394	9.500433	4.307283	3.751451	2.812379	1.258392	1.970727	1.254358	2.709871	2.332179	8.660519	1.440902	4.386526	4.524476
**TCGA-FV-A4ZQ**	0.032877	0	9.985124	1.215402	1.994133	5.257677	8.047984	7.679584	9.843052	4.608416	3.684267	1.290956	1.440173	2.417166	1.360338	2.613075	2.228249	4.509992	1.380059	5.686429	4.571149
**TCGA-DD-AACA**	6.30411	0	8.934133	1.215402	2.676101	4.609124	6.944592	6.317347	8.959861	4.867107	4.929601	1.137595	1.217047	2.469063	1.254358	4.088346	3.075175	4.608323	1.4011	5.149598	3.934969
**TCGA-K7-A5RF**	1.728767	0	9.073044	1.215402	1.394555	3.274205	6.769882	6.01049	8.704878	3.599124	3.105132	2.553942	1.233289	1.314751	1.352428	3.148081	2.066077	7.664429	1.399674	3.681446	2.317503
**TCGA-DD-A1EB**	5.526027	0	9.340185	1.215402	3.262545	3.969593	4.072489	5.77664	9.614518	5.334127	4.721066	2.282214	1.274692	1.661003	1.347592	2.696197	1.958272	8.142108	1.389392	2.770535	3.662625
**TCGA-DD-AAVU**	6.032877	0	9.158198	1.281183	2.903413	2.693957	6.740129	5.957684	9.104372	5.992956	2.668254	1.264378	1.234623	1.760083	1.307381	1.686487	2.243976	3.923101	1.411106	5.566215	3.738529
**TCGA-2Y-A9H0**	10.06849	0	9.104952	1.386052	2.38762	5.285343	7.737584	6.428597	9.587322	3.763791	3.269119	2.863239	1.232318	2.067824	1.254358	2.998473	2.581834	6.603208	1.380059	5.585314	4.356223
**TCGA-GJ-A6C0**	0.084932	1	9.601993	2.612397	1.608963	4.985787	9.033243	6.803545	9.436644	5.895301	4.225785	2.689795	1.337155	2.542084	1.348345	5.329175	2.74294	6.413311	1.380059	5.249478	4.118472
**TCGA-ED-A4XI**	2.243836	0	9.27111	1.529113	1.667596	3.974841	6.171452	6.972655	9.042898	3.267323	3.89432	2.001715	1.231832	1.595566	1.43172	2.011986	2.561007	6.27818	1.416499	3.907752	3.199788
**TCGA-G3-A7M8**	1.178082	0	9.370254	1.215402	1.840062	2.92733	5.16884	5.874703	9.564637	3.465733	3.526784	2.064007	1.287881	1.28294	1.527762	3.884431	2.228506	5.09153	1.421756	4.003276	3.305298
**TCGA-CC-A3M9**	0.821918	1	10.74554	1.998014	1.432955	5.758733	6.785712	8.477628	10.2814	4.825136	4.836919	1.125434	1.312934	2.099558	1.254358	5.572599	3.423722	2.102777	1.413581	5.816599	5.501994
**TCGA-MR-A8JO**	0.90411	0	9.086684	2.53065	2.476648	3.690249	5.963361	6.98639	8.632648	2.04492	5.034658	1.258058	1.325948	1.419268	4.751	5.784899	3.546731	3.451422	3.6146	3.907185	3.783978
**TCGA-DD-A3A6**	8.926027	1	7.787423	1.812429	3.242437	2.084872	4.33956	6.020852	7.594208	4.100375	5.114474	2.261111	1.210141	1.756329	1.254358	4.469603	2.632472	3.236622	1.92184	4.568196	2.392882
**TCGA-G3-AAV4**	0.073973	1	9.837439	1.215402	4.372557	4.466003	7.067048	7.094732	9.700519	4.913824	4.221173	1.06929	1.293787	2.60725	1.254358	4.99756	4.141953	6.191637	1.404215	4.534528	3.631418
**TCGA-DD-A11D**	4.273973	1	9.26649	1.215402	1.763631	3.856786	5.231076	6.197876	9.254893	3.643002	4.155381	2.591965	1.565772	1.461261	1.254358	4.311648	2.744024	7.160476	1.414479	3.928115	2.746877
**TCGA-BD-A3EP**	1.120548	0	10.17219	1.803544	3.339266	4.208874	6.941212	7.287337	9.508558	4.323388	4.385146	1.700153	1.283345	1.726393	1.628999	4.314162	2.380182	6.096916	1.619736	4.511433	3.861341
**TCGA-EP-A12J**	1.561644	0	8.659332	1.215402	2.372185	4.538373	8.638313	6.904526	9.317351	4.163982	3.78047	1.60588	1.210141	1.462486	1.254358	3.403613	3.374065	6.967021	1.414592	4.429615	3.265332

**TCGA-4R-AA8I**	0.717808	1	9.886878	1.359996	1.967845	4.650197	6.329885	6.528743	9.803802	4.135077	4.613464	3.279124	1.97962	2.023949	1.254358	3.77052	3.195301	6.18166	1.403648	5.119531	2.95825
**TCGA-CC-A7IF**	1.778082	1	8.156866	1.215402	2.614431	4.228118	6.517875	5.644653	8.353437	4.862734	3.754981	1.329114	1.356086	2.727402	1.336446	2.857295	1.940816	5.372328	1.451609	5.003264	4.033058
**TCGA-CC-A5UE**	0.745205	1	9.916622	1.837319	2.23156	5.045603	8.174522	8.435195	11.06924	6.126142	3.528925	3.039868	2.459753	3.022982	1.56482	3.059451	3.250288	4.866275	4.421711	6.718422	5.235114
**TCGA-HP-A5MZ**	0.249315	1	10.00503	1.653373	2.361914	4.381734	6.934746	6.622493	9.885967	3.433591	4.188733	1.753892	1.470087	1.290456	1.374947	3.882159	2.815223	5.24777	1.380059	4.288453	3.685918
**TCGA-CC-A8HU**	0.942466	1	9.657468	1.288875	2.554252	5.644894	8.107598	8.012291	10.06382	5.013956	4.015241	2.29476	2.556808	2.766308	1.313607	3.030457	3.917155	3.898719	4.245362	6.276718	4.696195
**TCGA-DD-AAD5**	3.684932	0	9.225053	5.912228	2.533473	4.300214	8.297821	7.444825	10.00176	4.926426	3.217089	2.010621	1.33827	1.362532	1.254358	3.672588	4.706088	2.399269	1.426766	5.01527	5.438824
**TCGA-G3-A5SI**	2.10411	1	9.677159	1.215402	1.355888	5.11738	6.059603	7.153629	9.17285	3.751931	3.686121	1.128648	1.318722	2.841346	1.254358	3.887814	1.696575	6.894586	1.380059	4.510898	4.08433
**TCGA-KR-A7K2**	2.271233	0	9.724033	1.281874	1.659475	3.326243	5.60173	7.200103	8.789371	2.645184	3.655554	2.419267	1.329096	1.339296	1.307938	3.015711	3.769259	4.821529	1.401038	4.051935	3.038221
**TCGA-DD-AADM**	0.032877	1	9.111836	1.333259	1.914473	3.103922	5.764022	6.843343	9.950148	4.388533	2.923176	1.525777	1.33905	1.767119	1.303026	2.091715	2.900841	3.428942	1.399105	4.962509	4.415985
**TCGA-XR-A8TG**	2.460274	0	9.765867	1.282564	1.712379	5.429063	6.572222	7.592394	9.499982	4.376965	4.113142	1.968628	1.283694	2.19234	1.457487	5.851321	2.69096	6.640321	1.380059	4.873259	3.641025
**TCGA-DD-AADJ**	2.920548	0	9.027714	1.745996	1.464722	3.100583	5.574443	6.07091	8.453788	4.806386	3.695475	2.300719	1.210141	1.320802	1.380824	3.46811	1.671528	5.188407	1.77927	4.678681	3.936122
**TCGA-ES-A2HS**	1.884932	1	8.991224	1.270774	1.63088	2.26945	5.815962	5.378055	8.022082	4.279234	3.679207	1.23506	1.210141	1.505841	1.254358	1.538513	3.67286	4.868041	1.380059	5.236212	3.037077
**TCGA-HP-A5N0**	2.060274	1	8.857241	1.284289	1.796844	4.036736	6.028548	6.577819	9.239442	5.288784	4.619527	2.11638	1.235834	1.487266	1.254358	3.868971	1.853359	7.70026	1.433635	4.605313	3.484265
**TCGA-ED-A7XP**	1.09589	0	9.964654	1.215402	3.327842	4.576565	5.979824	6.486971	10.20662	3.4014	3.948886	1.885425	2.127107	1.555205	1.964312	3.167897	2.069943	4.729907	1.380059	4.539097	4.468481
**TCGA-G3-A3CH**	2.136986	0	8.624415	1.215402	1.614259	4.316137	6.265598	6.279286	8.897687	5.315119	3.882656	1.924953	1.210141	2.222493	1.254358	4.160325	2.149048	6.225061	1.398422	4.644709	3.689106
**TCGA-BC-A217**	3.827397	1	9.075637	1.584827	2.336094	5.38427	5.551296	7.592861	9.887257	5.824161	3.685244	3.926304	1.35883	3.077464	1.254358	2.293452	3.887759	5.674131	1.380059	5.546112	3.023928
**TCGA-CC-A5UD**	0.832877	1	10.56705	1.359564	2.419466	5.43798	7.550257	7.409984	9.829102	5.467956	4.34732	1.236977	2.146446	3.512449	2.00056	3.88978	4.626826	4.573215	1.380059	7.10136	5.438116
**TCGA-EP-A26S**	1.665753	0	9.472273	1.215402	1.640572	3.776311	6.347064	6.990433	9.369312	4.601792	3.614271	1.232532	1.226353	1.501201	1.389583	3.733802	2.468695	5.859834	1.414142	4.450392	3.95265
**TCGA-BC-A110**	5.79726	1	8.574296	1.215402	1.424857	3.331256	5.657808	6.031488	8.304234	3.435368	3.308228	1.676479	1.31191	1.53384	1.466567	4.700396	2.606175	6.972721	1.380059	3.197706	2.9814
**TCGA-G3-A5SM**	1.424658	0	9.529712	1.689711	2.775797	3.865793	5.324886	6.259035	9.195401	4.443916	4.065904	1.830361	2.285098	1.381709	1.254358	2.98757	2.116464	5.524499	1.399105	4.509178	3.666157
**TCGA-ED-A8O5**	1.112329	0	9.764127	1.215402	1.894121	3.907336	8.758523	6.934223	8.029762	3.719057	4.258076	1.689607	1.277622	1.41917	1.351677	5.201934	2.312248	6.151512	2.704369	4.505079	2.815721
**TCGA-FV-A3R2**	0.531507	1	9.961472	1.343149	2.598285	4.621996	7.453545	7.359532	9.83356	4.673785	4.424835	2.416525	1.281946	2.160463	1.254358	4.23616	2.175232	5.972988	1.400697	5.186351	4.434619
**TCGA-DD-A116**	4.443836	1	9.810032	1.215402	2.654017	2.848167	6.191101	5.641023	8.469646	5.617629	3.660638	1.09052	1.510496	3.813647	2.050371	2.580971	2.327582	4.375122	1.405234	5.102675	3.7965
**TCGA-DD-A39X**	4.641096	1	9.381021	1.311551	3.194212	3.04731	8.181037	6.923052	10.43195	4.004695	3.340288	1.716029	1.315321	1.689319	2.754475	2.785104	1.872767	4.193026	1.403082	5.243704	5.132621
**TCGA-2Y-A9H2**	4.742466	0	9.053365	1.621761	1.995552	4.606701	5.985898	6.626128	9.302173	4.069853	3.538957	2.703409	1.210141	1.661721	1.360125	2.620503	2.933149	4.705407	1.796994	5.236487	5.380373
**TCGA-ZP-A9D2**	2.09589	1	9.537317	2.922696	4.220174	4.976787	7.327905	7.774639	10.24299	4.436117	4.087809	3.281164	1.236682	1.589229	1.418722	3.909886	2.991695	8.849016	1.446268	5.568687	4.040961
**TCGA-DD-A4NK**	3.315068	1	9.181743	1.215402	2.080394	3.752921	6.080177	6.143461	8.918463	4.20932	3.94811	1.940319	1.261479	1.362532	1.60065	3.208021	3.190688	6.715693	1.40206	3.771446	3.256484
**TCGA-FV-A4ZP**	6.810959	1	9.362964	1.215402	2.559016	2.089589	10.41507	6.271343	8.086351	5.641068	3.617178	2.586663	1.210141	1.734229	1.306824	1.999374	2.827637	2.008229	1.488637	6.845434	3.469127
**TCGA-DD-AAE2**	1.747945	0	9.219688	2.153714	1.516947	4.07583	5.829602	6.339922	9.404315	5.482705	4.476647	2.05919	1.324146	1.62009	1.254358	3.681951	2.022806	9.026018	1.405121	4.553153	2.884043
**TCGA-DD-A39Z**	1.646575	1	8.611361	1.286928	1.892509	3.500088	4.502156	7.042111	8.457989	4.754396	3.257941	2.221104	1.262902	2.034528	1.254358	2.756374	3.232626	4.684941	1.402628	4.789776	3.764729
**TCGA-5R-AA1D**	1.230137	0	9.368713	1.32095	1.897828	4.301438	5.971318	7.031457	9.173794	2.983853	4.579799	1.639825	1.288345	1.258656	1.722227	4.394224	2.706879	6.044255	1.81023	3.835629	2.887073
**TCGA-2Y-A9H7**	3.2	0	9.804418	1.215402	1.410685	3.672557	6.506215	6.083556	7.406486	3.091286	3.683177	2.265412	1.210141	1.964417	1.254358	1.756327	2.526855	5.065213	3.088473	4.942661	4.660766
**TCGA-CC-5264**	0.279452	1	10.12702	1.370314	2.917554	5.164439	11.01337	8.268939	10.08738	4.743568	4.007214	3.59218	1.34372	2.8627	1.297755	3.767689	4.322348	8.012599	1.561396	5.538465	4.490946
**TCGA-UB-A7ME**	1.331507	0	9.53213	1.389849	1.660324	3.737902	7.441696	7.508723	9.786322	5.036143	4.429802	1.52986	1.494982	2.250639	2.05725	2.845222	1.77991	3.40521	1.418179	4.748324	3.990356
**TCGA-UB-A7MD**	0.142466	1	9.999412	1.839649	2.781805	4.822307	7.044407	6.726992	10.52991	5.724572	5.279742	2.184331	1.457298	1.514165	1.254358	5.67034	3.267657	3.244051	1.4515	5.077214	4.072656
**TCGA-RC-A6M6**	0.024658	0	10.21877	2.308801	2.376487	4.631446	10.19531	7.142683	10.51189	6.183512	3.836827	2.50714	4.318795	1.970665	4.876634	3.128194	3.028073	2.044751	1.390539	6.365696	5.078937
**TCGA-DD-A3A8**	0.030137	1	8.892219	1.215402	1.744939	3.624661	6.483666	6.447754	8.892806	4.099732	3.667646	1.138454	1.253627	1.801692	1.254358	3.944358	2.384994	5.806194	1.661661	4.320403	3.215778
**TCGA-ED-A7PX**	0.016438	0	10.13683	2.754689	2.263601	5.142079	6.49261	7.866436	9.434637	3.437627	4.405775	3.283322	1.236803	1.677804	1.69328	4.053046	2.094986	5.211888	1.740364	5.152686	4.250547
**TCGA-DD-AACZ**	0.468493	1	9.649521	1.45635	3.282215	5.071251	9.055724	8.318903	10.6476	2.261839	3.992409	2.01781	1.391179	1.276914	1.254358	4.906438	2.635912	3.898052	2.003214	5.606516	5.291696
**TCGA-DD-A39V**	1.761644	1	9.878939	1.356104	2.39981	3.665671	5.47841	6.538412	9.789506	5.165259	3.791664	1.258157	1.237166	1.385127	1.312831	3.309072	1.885037	4.560388	1.39157	5.088881	4.707126
**TCGA-G3-A25Z**	1.794521	0	9.971494	1.215402	1.588446	2.995156	4.455428	6.672635	9.22793	4.729857	3.204239	1.18365	1.507098	2.149345	1.386879	2.912217	3.910947	4.221859	1.380059	5.153031	3.810443
**TCGA-5R-AAAM**	0.126027	1	9.610247	1.215402	1.937807	3.983706	6.785294	6.456481	9.388566	4.062086	3.62374	1.326036	1.36102	1.496269	1.606531	4.07339	2.30561	6.65882	1.402514	4.070706	2.973784
**TCGA-ES-A2HT**	1.2	1	9.47005	1.259805	1.856713	2.443785	4.702163	6.392336	9.552342	3.285018	3.398116	2.052704	1.226597	1.221516	1.862909	2.281752	2.704212	3.966202	1.380059	4.216345	2.834069
**TCGA-DD-A3A7**	1.147945	1	8.81635	4.767888	2.919219	1.963707	7.73194	6.477378	8.063813	4.425785	3.54347	1.799615	1.210141	1.167058	1.254358	1.894011	4.102535	3.510015	1.380059	5.490134	4.385231
**TCGA-G3-A7M6**	1.731507	0	10.72873	1.524785	2.243352	4.825095	8.165797	8.394247	9.438324	4.683236	4.407844	1.968575	1.285208	1.550665	1.309609	4.189345	4.161006	5.164699	1.412344	5.599494	5.477551
**TCGA-DD-AAD6**	1.841096	0	8.418928	1.215402	1.562995	3.674314	7.133335	5.403789	8.821047	4.549142	3.863378	1.10495	1.210141	1.467089	1.254358	3.175601	4.105112	2.371283	1.380059	4.683521	3.53933
**TCGA-FV-A2QQ**	1.99726	0	9.90714	3.502075	1.398765	3.71139	6.877401	6.26928	9.457372	3.31855	3.754631	1.982795	1.356965	1.482277	1.302242	2.794276	3.296421	4.025739	1.389507	4.617014	4.757712
**TCGA-2Y-A9GX**	6.690411	0	9.172476	1.283944	1.765266	5.049997	6.352886	6.525201	9.599675	3.876767	3.891049	1.728589	1.235713	1.612636	1.508031	3.40395	1.753771	6.635633	1.380059	3.82233	4.180354
**TCGA-BD-A3ER**	3.054795	0	9.041866	1.373841	1.555624	3.517176	5.797013	5.848187	9.235056	3.665149	4.237926	1.789685	1.270578	1.406023	1.298766	5.04352	3.012874	6.112259	1.414479	3.734646	4.260776
**TCGA-DD-AADC**	1.164384	1	10.4109	2.441811	2.884538	4.625953	7.358639	8.044401	10.6131	5.666358	4.769641	2.262449	2.504599	2.45224	1.254358	3.603802	3.508326	5.32511	1.941089	4.878404	4.460233
**TCGA-BC-A10Z**	0.093151	1	9.405074	1.215402	2.122244	4.747559	4.729731	7.460843	9.446753	4.641076	4.269762	1.320888	1.357952	1.751453	1.254358	3.291802	2.451112	3.445239	1.380059	5.086071	4.399906
**TCGA-5C-AAPD**	0.054795	0	9.280642	1.518927	1.597677	3.680188	7.087076	8.084762	9.488393	4.49112	4.253733	1.35591	1.418533	2.295411	1.308495	2.805489	3.335553	6.614904	1.380059	5.784929	4.072177
**TCGA-DD-AAED**	2.090411	0	8.420955	1.215402	1.40076	1.438205	6.923394	7.961449	7.994459	3.112403	3.544248	1.473975	1.239824	1.301051	1.254358	2.270152	2.752849	2.730394	1.380059	3.772818	3.552912
**TCGA-DD-AACI**	4.432877	0	9.484907	1.860708	1.905416	3.23592	5.763587	6.550996	9.65218	2.283668	3.529445	2.633202	1.210141	1.968875	1.254358	2.052696	1.962717	4.627996	1.380059	4.817369	3.921896
**TCGA-G3-A25Y**	1.238356	1	10.1853	1.215402	2.349048	4.750058	6.626438	7.845162	9.020042	4.192051	4.660716	2.460706	1.339718	2.656711	2.180462	5.628329	2.039762	6.290682	1.715009	5.715053	5.185398
**TCGA-PD-A5DF**	1.750685	1	10.06563	1.75211	1.892018	5.158738	5.648485	6.658357	9.942945	5.70838	4.183928	1.95407	1.258511	2.703481	4.57702	4.535852	3.034512	7.356861	1.380059	5.307153	3.809732
**TCGA-DD-A114**	3.147945	1	10.39941	2.75414	4.439859	5.462832	7.956883	8.005183	10.39518	3.887104	4.995712	2.870575	1.256488	1.833434	2.100404	3.613046	2.074527	5.976414	1.723293	5.628552	4.416274
**TCGA-DD-AAVY**	5.39726	0	9.526861	1.215402	2.371617	4.041256	5.26688	6.647469	9.752452	4.490081	3.850515	1.116524	1.254223	1.507383	1.254358	3.54306	2.231155	4.116747	1.380059	4.057627	3.763763
**TCGA-BC-A10S**	3.89863	1	9.454646	1.2632	1.924215	3.719541	6.611424	6.526346	8.595444	4.084864	3.66634	1.161379	1.591194	1.292626	1.330231	3.892854	3.871215	5.355449	1.395116	4.25498	3.799311
**TCGA-CC-A123**	0.6	0	9.694427	1.277261	1.265571	1.756834	7.240811	7.16535	8.082004	4.419192	3.143059	1.118088	1.210141	2.120985	1.931284	2.855956	2.780343	3.228365	3.190595	5.729604	3.70784
**TCGA-MI-A75H**	2.046575	0	8.78263	1.215402	1.657692	3.89791	5.144026	6.534569	9.571357	4.822802	3.916377	1.096677	1.309402	1.329664	1.254358	3.08014	3.448935	4.425108	1.40172	4.532915	3.676516
**TCGA-DD-AADG**	3.136986	0	8.976701	1.215402	1.46512	4.127616	4.775758	6.283999	8.082191	4.57808	3.855756	1.229492	1.232803	1.887193	1.303473	3.885138	3.023468	5.06273	1.389736	4.59034	3.270647
**TCGA-BC-A10U**	2.293151	1	9.497504	3.874217	2.556942	3.858207	8.918004	7.029205	9.551131	4.363355	3.450021	2.085074	1.247764	3.729292	1.254358	4.054786	2.217936	5.268081	1.396143	4.792994	3.674083
**TCGA-CC-A7IG**	0.819178	1	10.00586	1.662244	3.580072	3.318917	8.197696	7.492637	9.826569	5.457861	4.083325	1.871594	1.601957	2.934536	1.390622	3.031983	4.468062	3.485933	2.445124	6.755972	5.395221
**TCGA-DD-A3A3**	1.465753	1	8.985528	1.215402	1.686089	3.205676	8.098076	6.297879	8.972919	5.05615	3.762393	1.496862	1.747963	2.144656	4.464496	2.396476	2.190918	1.967954	1.667361	5.13583	3.578422
**TCGA-2Y-A9GZ**	2.323288	1	9.208318	1.215402	1.449194	3.598825	5.958145	6.388386	9.538799	5.751669	4.157316	1.59962	1.324935	1.949816	1.254358	3.805434	2.813443	7.047749	1.390195	4.085048	3.911491
**TCGA-BW-A5NO**	0.054795	0	8.862478	1.215402	1.864416	4.090463	5.759149	7.143324	9.99079	6.512673	4.394462	2.422003	1.210141	3.280425	1.788578	3.237934	3.173006	5.695376	1.398536	4.748143	3.52283
**TCGA-DD-A4NJ**	2.542466	0	9.784838	1.415383	4.503083	5.22016	6.839857	6.919781	9.470611	4.406405	4.16662	1.151656	1.287184	3.062269	1.254358	4.375901	3.958475	5.604053	1.461355	4.802858	4.426393

**TCGA-DD-AACS**	4.942466	0	9.341574	1.215402	2.239135	4.722817	7.478296	7.479125	10.29234	5.728667	3.598886	1.192691	1.249921	2.760913	1.339812	3.397855	1.144353	6.060993	1.380059	5.364274	3.57829
**TCGA-UB-AA0U**	0.89589	0	9.237807	1.215402	2.148799	5.442263	6.777323	6.524753	9.375138	4.54049	4.04227	1.551146	1.235108	2.410977	1.360338	2.971391	2.468345	7.203803	1.380059	5.058082	4.559378
**TCGA-CC-A9FW**	0.679452	0	10.57305	1.64657	2.935571	4.086668	8.069372	6.872644	9.228725	4.738089	4.576442	1.998941	1.29159	2.197716	1.299664	2.374594	2.612455	4.807104	1.415153	5.050455	4.330552
**TCGA-G3-AAUZ**	1.315068	0	8.776003	1.215402	1.66312	3.771059	5.64699	6.245086	8.877998	5.146838	3.057032	1.06929	1.375164	1.426078	1.254358	2.403508	3.815463	5.553667	1.380059	5.635631	3.776046
**TCGA-G3-A25U**	4.482192	0	9.441638	1.215402	1.692467	4.76374	6.67903	6.829822	9.365675	5.263876	3.896315	1.06929	1.210141	1.394625	1.254358	3.444987	2.348808	8.139559	1.380059	4.814119	3.714229
**TCGA-DD-AAEK**	2.923288	0	9.017794	1.215402	1.905971	4.303496	6.035157	6.193602	8.742713	4.318359	4.068284	1.117977	1.210141	2.024008	1.254358	3.050378	2.369512	6.412763	1.380059	4.49649	2.98487
**TCGA-DD-AAEH**	2.147945	0	8.680015	1.215402	1.888505	4.046883	6.65795	6.55333	9.269517	4.667545	3.309915	1.095426	1.258511	1.447426	1.254358	2.623297	1.993009	5.504808	1.421086	4.082405	3.370712
**TCGA-2Y-A9HA**	0.09863	1	9.927784	3.26326	3.57031	5.107187	9.03098	6.843257	9.982565	5.603395	4.935707	1.629364	1.600786	2.275223	1.447545	4.859768	3.513044	4.553775	1.45844	4.858436	3.03485
**TCGA-DD-AACG**	1.284932	1	8.964604	5.736748	1.577422	2.580193	7.665951	7.380294	9.563607	5.65053	2.820533	1.481109	2.369665	2.548781	1.8108	2.215585	1.879949	3.903406	3.813428	5.553201	4.191724
**TCGA-DD-AAD2**	1.80274	0	9.713857	1.215402	1.531076	3.961682	7.028992	6.83977	9.818401	4.110283	3.759525	1.767524	1.302079	2.031716	4.206047	3.231192	1.555922	5.818519	1.393401	4.729505	3.862385
**TCGA-DD-AAEI**	4.194521	0	9.480324	2.520844	1.300796	3.670921	5.676483	6.493954	9.721199	4.980534	3.324164	1.115517	1.709439	1.431789	1.254358	2.996788	2.690931	4.581542	1.380059	4.990596	3.765483
**TCGA-G3-AAV5**	0.969863	0	10.48521	1.215402	2.776613	3.75576	4.971988	6.757132	9.761657	4.123221	3.044917	1.06929	1.413194	1.452562	1.254358	3.430751	4.168805	3.581882	1.387784	5.229834	5.346675
**TCGA-DD-AADK**	2.873973	0	9.271096	1.312224	1.947432	3.645599	5.19452	5.634676	8.333505	4.016837	3.417848	1.756863	1.234502	2.515193	3.200644	2.442124	1.951115	5.04581	1.54476	4.52501	4.305637
**TCGA-FV-A3I0**	2.323288	0	8.728729	3.395734	1.914128	4.9108	7.015544	7.308469	9.197032	2.783056	4.355994	3.586242	1.210141	1.732425	1.316265	4.342491	2.497071	2.599015	1.561198	4.740153	3.276575
**TCGA-2Y-A9GS**	1.983562	1	10.45409	1.215402	2.95782	4.494626	6.411642	7.713947	10.69846	5.38193	3.898816	1.595774	1.423224	3.733277	1.254358	1.92518	3.508502	5.322574	1.40172	5.759164	4.445562
**TCGA-ZP-A9CV**	2.980822	1	9.942732	1.358268	1.512551	4.660894	4.966869	7.452078	9.916417	4.23491	3.98584	2.725181	1.316456	1.920264	1.254358	2.950603	2.265733	6.042505	1.380059	4.336791	4.127242
**TCGA-ZP-A9D1**	0.057534	0	9.889276	1.215402	1.511977	4.687201	5.637517	6.395748	9.133879	3.088161	3.910038	2.782153	1.236197	1.49196	1.310832	4.240209	2.531343	6.242395	2.869783	3.848015	4.332527
**TCGA-DD-AADV**	1.572603	0	9.440276	1.447142	2.368059	4.284997	7.040025	7.265232	10.18519	4.554724	4.177064	2.235435	1.477877	1.542502	1.254358	2.849057	2.602585	6.342299	1.387899	5.279598	3.648298
**TCGA-BC-A10Y**	1.947945	1	9.199874	1.323844	2.099309	4.401188	7.08474	7.282553	9.835952	5.106148	4.880843	2.176259	1.419577	1.673152	1.383542	4.702054	3.571345	6.436664	1.380059	5.525562	3.591781
**TCGA-DD-AADR**	5.556164	0	9.149402	1.965065	1.750388	3.900665	6.903476	5.502931	10.26499	5.180864	2.744826	1.965127	1.210141	2.385374	1.254358	3.351261	2.961856	4.715849	1.380059	4.644671	4.326478
**TCGA-EP-A2KC**	0.052055	1	9.705239	1.215402	2.906994	4.363123	7.017433	6.488131	9.025178	4.617285	4.329574	1.694654	1.251118	1.675047	1.572661	3.289181	3.801764	4.355902	1.414816	4.775067	4.619081
**TCGA-DD-AACJ**	5.758904	0	9.755311	1.252752	2.735037	3.130242	6.094117	5.79911	10.10373	4.972612	3.579193	1.54063	1.629738	1.194771	1.254358	2.450872	3.850731	3.537168	1.380059	4.246222	4.502644
**TCGA-WX-AA44**	1.684932	0	9.89053	1.764168	2.649346	4.897501	7.396891	7.1579	9.349249	4.31316	4.122705	2.213328	2.383573	1.44895	1.403525	2.957007	4.213216	5.249983	1.439913	5.744261	5.162679
**TCGA-DD-A113**	6.643836	0	9.455308	1.263317	2.378609	4.966206	6.652077	7.139073	9.86901	2.641806	3.97161	2.159397	2.352462	2.803254	1.254358	3.995102	2.239957	6.172222	1.89198	3.765669	4.9451
**TCGA-DD-A1EC**	1.649315	0	10.36965	1.215402	1.626789	4.760947	5.249889	7.192657	9.289534	2.467166	4.31841	3.410288	1.228548	1.882887	1.634402	4.27106	2.006945	3.721938	1.426433	4.536057	5.265356
**TCGA-BC-A10Q**	3.109589	1	9.264931	1.27703	2.232145	5.255076	7.914791	6.7242	7.809777	3.194431	3.602135	2.86304	1.210141	1.539416	1.254358	4.967618	3.906972	7.041859	1.418627	5.658155	4.225422
**TCGA-2Y-A9H3**	4.153425	0	8.502587	1.422478	1.794491	2.564164	5.854528	6.62908	8.273881	1.663774	3.543914	1.154798	1.210141	1.290456	1.855128	2.647447	1.790331	3.760602	1.61181	4.643382	2.75693
**TCGA-DD-A4NH**	2.512329	0	9.421266	3.100308	1.82459	4.651187	8.044493	6.784516	8.715967	4.46648	3.907541	3.111401	1.259224	2.289603	1.811523	4.760366	2.789921	5.493911	1.471768	5.460179	4.767953
**TCGA-CC-A7IE**	0.594521	1	9.246618	1.825306	1.457934	4.124427	9.469926	7.05565	9.290501	4.582677	4.491576	1.303402	6.566301	1.644448	5.089001	5.042268	1.717304	1.65921	3.147221	5.133984	4.827408
**TCGA-DD-A4NQ**	1.021918	1	9.583039	1.926668	2.03244	4.100388	9.219944	7.347387	10.38331	4.702752	3.170446	2.404047	1.425927	2.188484	1.254358	3.506386	2.074138	3.599454	1.416947	4.503466	4.911785
**TCGA-DD-A4NG**	2.19726	1	9.115459	1.215402	2.425692	4.611937	5.362607	5.718416	8.183416	4.035261	4.055825	1.122546	1.226963	2.418442	1.254358	4.06173	2.127036	5.062115	1.380059	3.448362	3.712102
**TCGA-DD-AAW2**	5.082192	0	9.052821	1.215402	1.863698	4.239189	7.240516	6.596852	9.19447	3.675188	3.934704	1.66376	1.245724	1.368347	1.403114	3.408286	2.925351	6.048272	1.425099	3.823066	3.133606
**TCGA-DD-A73B**	0.775342	1	10.68192	1.493566	1.360233	4.221365	6.652513	5.849738	8.742565	4.342047	3.862278	1.877251	1.210141	2.434789	1.295505	3.674307	2.921815	7.901232	1.380059	4.127219	3.327166
**TCGA-MI-A75G**	1.912329	0	9.198685	1.215402	1.485548	4.048442	4.832881	6.343518	9.547442	5.884584	3.900514	1.504739	1.36408	1.668164	1.254358	4.128156	1.873804	4.636542	1.4058	4.735667	3.854548
**TCGA-BC-A10X**	2.109589	1	8.913263	1.260391	1.553405	3.639823	5.58673	6.546101	8.745517	3.60236	3.132698	1.88756	1.210141	1.392831	1.425088	2.80844	1.22825	8.590996	1.874683	3.477047	2.497675
**TCGA-CC-A3MC**	0.994521	0	9.118643	1.215402	2.023653	4.668345	6.913535	6.534732	9.742444	4.593852	3.623792	2.408848	2.940448	3.426777	1.801932	2.271755	2.326912	4.296245	1.524721	5.276647	6.050295
**TCGA-DD-A73G**	9.528767	0	8.538708	1.604406	1.789999	2.08836	5.781115	7.251763	8.46145	3.565339	3.760873	1.176663	1.348814	1.397807	2.684168	2.091178	2.315235	3.600388	1.655295	5.651302	3.489464
**TCGA-CC-5260**	0.238356	1	10.1969	1.215402	4.187115	5.997916	7.187509	8.284475	8.177887	4.599618	4.704841	3.572609	1.210141	3.038958	1.254358	4.400852	3.939488	4.236787	1.475502	5.71185	5.87008
**TCGA-DD-A4NL**	4.687671	0	8.612078	1.407208	1.38694	2.970486	5.413195	6.136057	8.608588	3.509795	3.382019	1.879053	1.59835	1.311661	1.254358	2.934076	2.386044	7.175157	1.452804	3.375305	2.193982
**TCGA-DD-A3A1**	0.638356	1	9.33438	2.508318	1.848088	3.319646	7.530324	5.6892	10.31155	5.624479	3.691238	1.889231	1.210141	1.728055	1.254358	3.159282	3.627381	5.743198	1.380059	4.96799	4.756524
**TCGA-G3-AAV2**	1.019178	0	9.997642	1.215402	1.466811	3.084921	7.329727	6.911103	9.437482	4.97833	3.447585	1.300446	1.233774	2.040889	1.49025	2.722197	3.237282	4.864575	1.539934	4.566704	3.697856
**TCGA-DD-A4NV**	6.569863	0	8.733149	1.215402	1.708226	4.096111	5.139975	6.188104	8.535582	5.250776	4.253315	1.529547	1.231832	2.291637	1.390414	3.701632	2.401537	9.192045	1.416611	3.998319	3.8527
**TCGA-ED-A627**	1.158904	0	9.351685	1.215402	1.531736	4.627163	6.52963	7.064317	8.864418	3.468454	3.893113	2.160492	1.210141	1.442555	1.63356	4.72962	2.580911	6.576543	1.401493	4.353175	3.179042
**TCGA-BC-A3KG**	1.863014	0	8.786535	1.215402	1.655139	5.819694	6.254947	8.216681	9.928822	5.459752	3.69098	1.867378	1.23086	1.55049	1.254358	3.561603	1.811389	6.08228	1.949676	5.888591	4.093714
**TCGA-G3-AAV6**	0.178082	1	10.83993	3.634634	1.490334	4.391106	6.775392	7.078279	8.585343	5.661127	5.267136	1.120208	7.299766	2.378183	1.403833	2.455095	3.592452	2.349027	2.526165	7.234288	4.785535
**TCGA-DD-AAVZ**	5.205479	0	10.4767	1.215402	1.587186	3.749782	8.290199	7.150123	8.817189	2.375815	3.85714	2.448268	1.238133	1.423553	1.314826	2.712205	1.751477	4.904703	2.435779	4.376555	4.613037
**TCGA-CC-A9FU**	0.00274	0	9.861575	1.379484	2.396008	4.218627	6.911738	7.503432	9.357465	5.748302	4.674673	1.279153	5.324747	1.70504	1.468511	2.408991	2.97568	2.882155	3.794912	5.620051	5.377024
**TCGA-DD-AACE**	5.983562	0	8.007582	1.215402	1.87684	3.506363	4.589179	6.005705	9.259349	5.071311	3.336995	1.06929	1.23438	1.378584	1.254358	3.107168	2.027827	6.248179	1.380059	3.875193	2.893186
**TCGA-BC-A3KF**	0.021918	0	8.854059	1.29276	2.093672	3.885248	7.319409	6.746744	9.629001	4.926979	3.795632	2.21846	1.34815	1.686431	1.254358	2.678736	2.110954	6.616631	1.392372	3.949087	3.439859
**TCGA-ZS-A9CD**	3.79726	1	9.952109	1.984076	2.64333	3.635767	6.431035	6.417849	9.774423	4.180221	4.467764	1.766436	1.627799	1.284468	1.363848	5.097506	3.497698	4.52876	1.455083	4.353987	3.882283
**TCGA-2Y-A9H4**	3.978082	0	8.778846	1.297996	1.678341	3.684972	7.457444	6.913006	9.462049	5.461181	3.634812	2.740304	1.300586	1.379089	1.254358	2.376455	3.710507	6.373855	1.380059	5.457836	2.965505
**TCGA-WX-AA47**	1.523288	1	7.819015	1.396571	1.575787	2.499742	3.190585	6.222351	8.531357	2.503976	5.103081	1.161486	1.27961	1.501383	1.966088	2.829197	2.979448	2.64468	1.387554	3.457033	3.385894
**TCGA-DD-A3A5**	8.561644	1	8.941724	1.215402	1.411725	3.873958	5.42693	6.849569	10.07765	5.57817	4.223837	1.845704	1.261716	1.632658	1.254358	2.295522	1.815629	5.338605	1.391227	5.220287	3.60943
**TCGA-BC-A5W4**	1.49863	1	9.987496	1.709385	1.735485	2.904236	7.704636	6.781433	9.910375	3.613423	2.961675	2.330069	1.210141	2.810552	1.254358	1.300039	3.984089	6.066824	1.380059	5.208096	4.302812
**TCGA-RC-A7SF**	1.586301	0	8.863594	1.215402	2.325414	3.497942	6.55739	6.374835	9.136683	4.173927	3.388215	2.194165	1.359158	1.283267	1.394663	3.044735	3.758213	4.911876	1.399105	4.141421	3.527794
**TCGA-RC-A6M4**	0.060274	0	9.281345	1.215402	1.540468	4.035289	6.538658	6.316772	9.969256	4.698981	4.296619	2.090851	1.262191	1.922193	1.254358	1.997743	3.744533	4.333636	1.380059	4.617453	3.927748
**TCGA-ED-A7XO**	1.169863	0	9.703478	1.215402	1.572966	3.835534	6.410737	7.222473	9.912819	5.229751	3.883999	1.344659	1.44652	2.175908	1.316155	2.996577	3.592043	5.447655	1.380059	4.715797	3.147872

Supplementary Table S5. The results of HCC samples from the TCGA database that multivariate Cox regression analysis.

ID	coef
**HSP90AA1**	0.33829
**MAPT**	0.284749
**HDAC1**	0.270317
**RAC3**	0.342612
**BTC**	0.334404
**CHGA**	0.245928
**GAL**	0.195572
**STC1**	0.247046
**GHR**	-0.14759

Supplementary Table S6. Chi-square test results of clinical features of LIHC patients in TCGA database.

Covariates	Type	Total	Test	Train	Pvalue
**Age**	<=65	232(62.7%)	74(66.67%)	158(61%)	0.3602
	>65	138(37.3%)	37(33.33%)	101(39%)	
**Gender**	FEMALE	121(32.7%)	35(31.53%)	86(33.2%)	0.8466
	MALE	249(67.3%)	76(68.47%)	173(66.8%)	
**Grade**	G1	55(14.86%)	19(17.12%)	36(13.9%)	0.8681
	G2	177(47.84%	51(45.95%)	126(48.65%)	
	G3	121(32.7%)	36(32.43%)	85(32.82%)	
	G4	12(3.24%)	4(3.6%)	8(3.09%)	
	unknow	5(1.35%)	1(0.9%)	4(1.54%)	
**Stage**	Stage I	171(46.22%	52(46.85%)	119(45.95%	0.455
	Stage II	85(22.97%)	24(21.62%)	61(23.55%)	
	Stage III	85(22.97%)	23(20.72%)	62(23.94%)	
	Stage IV	5(1.35%)	3(2.7%)	2(0.77%)	
	unknow	24(6.49%)	9(8.11%)	15(5.79%)	
**T**	T1	181(48.92%	56(50.45%)	125(48.26%	0.0748
	T2	93(25.14%)	26(23.42%)	67(25.87%)	
	T3	80(21.62%)	21(18.92%)	59(22.78%)	
	T4	13(3.51%)	8(7.21%)	5(1.93%)	
	unknow	3(0.81%)	0(0%)	3(1.16%)	
**M**	M0	266(71.89%	80(72.07%)	186(71.81%	0.7547
	M1	4(1.08%)	2(1.8%)	2(0.77%)	
	unknow	100(27.03%	29(26.13%)	71(27.41%)	
**N**	N0	252(68.11%	79(71.17%)	173(66.8%)	1
	N1	4(1.08%)	1(0.9%)	3(1.16%)	
	unknow	114(30.81%	31(27.93%)	83(32.05%)	

Supplementary Table S7. The HCC patients' high-risk and low-risk results of the nomogram.

ID	futime	fustat	HSP90AA1	MAPT	HDAC1	RAC3	BTC	CHGA	GAL	STC1	GHR	riskScore	Risk	Nomogram
**TCGA-G3-A25X**	4.873973	0	9.409763	1.281519	7.210282	3.093527	2.297399	1.234017	1.306266	4.36522	4.743646	0.764852	low	0.470826
**TCGA-DD-AADS**	1.29863	0	7.909564	1.372748	5.299621	3.812261	1.389025	1.370612	1.254358	1.954272	3.41247	0.18281	low	0.214028
**TCGA-G3-AAV3**	1.128767	0	9.344611	3.096314	6.885689	3.779225	1.272327	1.583814	1.396523	3.135438	4.613829	0.860768	low	0.263259
**TCGA-DD-A73E**	0.120548	0	9.686737	3.912204	6.844208	4.530901	1.956301	1.241512	1.254358	3.772045	5.393782	1.828231	high	1.224976
**TCGA-DD-AAEG**	1.969863	0	9.134447	2.890903	6.868026	3.201183	2.186742	1.352014	1.544893	2.137698	6.235383	0.501399	low	0.249631
**TCGA-ED-A8O6**	0.153425	1	9.145738	1.550439	7.468652	5.803519	1.315811	3.177968	3.289903	3.710817	1.869739	4.562	high	1.930372
**TCGA-G3-AAV7**	0.989041	0	9.963573	2.93566	8.342488	4.492261	3.317779	1.353116	1.319028	6.031657	4.9245	6.914145	high	0.576021
**TCGA-CC-A7IJ**	1.046575	0	11.02695	1.462929	8.49314	4.619424	2.62515	1.733717	1.254358	5.611743	1.982414	8.482863	high	1.044698
**TCGA-CC-5261**	0.265753	1	10.50076	2.653866	7.557932	2.965371	3.949684	1.29575	1.347807	4.22172	5.943558	2.472265	high	0.642555
**TCGA-RC-A7SH**	1.282192	0	8.407089	2.318704	6.472636	5.29232	1.510014	1.210141	1.307827	2.850693	4.318761	0.71297	low	0.267664
**TCGA-DD-A1EI**	0.50137	0	10.07078	2.251952	7.013666	3.858943	2.301205	1.282179	1.603508	4.489831	6.145724	1.399435	high	0.474899
**TCGA-G3-A3CJ**	1.627397	0	9.016811	1.478284	6.205672	5.340397	1.089147	1.246804	1.254358	3.866509	2.705966	0.922884	high	0.743371
**TCGA-CC-A8HS**	0.821918	1	9.698237	1.426399	7.22109	5.136394	2.683495	1.973901	1.822901	3.522062	6.510217	1.676464	high	5.430538
**TCGA-G3-AAV0**	1.30411	0	7.865013	1.528811	6.120548	5.358484	1.925567	1.371047	1.254358	2.083636	7.251258	0.279893	low	0.195394
**TCGA-5C-A9VG**	0.89863	0	10.36627	2.112852	8.668209	3.621939	1.398718	1.289389	2.556653	2.843499	2.607748	2.147497	high	0.829235
**TCGA-BC-4072**	4.082192	1	10.20127	1.628096	7.939093	3.882541	2.437567	1.289041	4.691019	3.701314	5.519358	2.744444	high	2.989015
**TCGA-DD-AADB**	3.40274	0	9.66043	1.78572	7.310735	4.13628	1.484293	2.108178	1.405166	3.665716	3.731888	1.327957	high	1.195698
**TCGA-CC-5258**	0.353425	1	10.34974	1.286713	8.034325	4.429156	4.028567	1.85035	3.581841	3.757479	3.90228	6.562061	high	0.691127
**TCGA-DD-A73C**	1.920548	0	9.710457	2.977105	6.914562	4.983785	1.570688	1.235229	1.254358	2.682157	8.217953	0.742837	low	1.150368
**TCGA-DD-AAEE**	2.219178	0	9.144361	2.432832	6.476584	3.793001	1.941861	1.229765	1.254358	2.40347	4.185845	0.59386	low	0.425241
**TCGA-ED-A82E**	1.117808	0	9.210486	2.570141	6.69766	3.238474	3.775877	1.253268	1.346838	2.932621	7.585855	0.723409	low	0.586124
**TCGA-DD-AAE6**	0.386301	0	8.208498	2.316773	7.427705	3.956871	1.23506	1.754746	1.254358	2.437081	4.235984	0.514865	low	0.191057
**TCGA-DD-A3A2**	5.838356	1	8.177214	1.401808	5.676328	4.245633	1.639754	1.225621	1.254358	1.476846	6.783345	0.146982	low	0.466609
**TCGA-CC-A3MB**	0.863014	1	9.912439	4.553198	7.180453	3.605352	2.890799	1.63387	1.254358	3.700983	6.110596	2.513447	high	2.13641
**TCGA-DD-AAE1**	1.512329	0	9.620283	2.397169	6.484127	2.714117	2.557483	1.210141	1.254358	3.040672	4.246626	0.678156	low	0.228863
**TCGA-G3-A3CG**	1.843836	0	9.016853	1.817824	7.005727	4.15789	2.966166	1.377326	1.254358	4.395182	6.15988	1.114274	high	0.882283
**TCGA-DD-A4NA**	2.761644	0	10.05612	1.517995	6.924044	3.292139	2.396921	1.235229	1.709424	4.695046	6.782435	0.906283	high	3.30E-07
**TCGA-DD-A118**	9.416438	0	10.0526	2.967311	6.70308	1.45283	1.230202	2.043492	1.448928	4.498284	8.133071	0.420178	low	0.372143
**TCGA-CC-A9FV**	0.00274	0	9.942902	5.48324	6.450378	3.81873	2.839399	1.238133	1.254358	2.455386	2.682161	3.178184	high	1.748029
**TCGA-ED-A459**	2.493151	0	9.510187	1.562995	7.545389	3.82245	2.344684	1.30357	1.254358	2.842517	5.57831	0.748094	low	0.224394
**TCGA-DD-AAEA**	1.575342	0	8.341954	1.765422	6.257058	5.354448	2.106006	1.360363	1.417708	3.016242	5.472169	0.652801	low	0.290023
**TCGA-DD-AADP**	1.254795	0	9.44108	1.502459	6.479009	3.691437	1.770141	1.263257	1.254358	3.679408	6.558314	0.447571	low	0.201462
**TCGA-DD-AACT**	4.279452	0	8.866206	2.317367	6.137446	3.591705	1.781376	1.28416	1.254358	2.003785	4.704236	0.359059	low	0.229235
**TCGA-DD-A4NB**	2.709589	0	9.329346	2.312603	7.035541	3.492829	1.253495	1.210141	1.442393	3.679004	5.736354	0.573223	low	0.107107
**TCGA-DD-AADL**	1.742466	0	8.368305	2.225488	7.047244	5.329844	2.277315	1.288229	1.297755	2.148791	6.701287	0.630646	low	0.449129
**TCGA-DD-A4ND**	7.523288	0	10.06203	1.683345	7.307398	3.127134	2.392781	1.351463	1.500309	3.997487	7.108186	0.789522	low	0.236354
**TCGA-DD-AAW1**	5.449315	0	9.778331	1.605774	6.123471	4.167501	1.212731	1.210141	1.254358	4.653817	4.569067	0.77216	low	0.557302
**TCGA-DD-A1EA**	6.616438	0	9.429983	1.712135	6.752069	4.7158	1.876203	1.210141	1.254358	4.144835	6.058889	0.893954	high	0.994941
**TCGA-ED-A66Y**	0.810959	1	10.01571	1.578692	7.554728	2.53112	2.331133	1.210141	1.254358	4.792142	3.073779	1.308831	high	1.965862
**TCGA-DD-AACH**	0.534247	1	9.855498	3.598118	8.30829	4.766375	3.916351	1.296096	1.254358	3.843557	4.96232	6.035533	high	1.323872
**TCGA-DD-A3A4**	1.676712	1	9.490199	2.536085	7.673511	4.127605	1.623302	1.232682	1.254358	2.179031	4.492634	0.866699	low	0.524581
**TCGA-DD-A11A**	0.216438	0	9.749902	1.898386	6.818327	5.814024	1.621274	1.48542	1.254358	4.686368	4.984978	2.050462	high	0.909682
**TCGA-DD-AAE7**	1.764384	0	9.403715	1.69914	5.772515	3.223085	1.831692	1.210141	1.333179	2.694851	5.959052	0.288273	low	0.252161
**TCGA-DD-AAE4**	1.665753	0	8.664595	1.29154	6.108782	4.291234	1.382815	1.210141	1.254358	2.941326	5.875568	0.287826	low	0.285318

**TCGA-DD-A4NF**	2.580822	0	10.37676	1.618918	6.863259	3.20846	1.143054	1.233532	1.254358	4.449996	5.193491	0.71103	low	0.252161
**TCGA-BW-A5NP**	0.00274	0	10.325	2.804884	7.722997	3.187653	1.141632	1.36266	1.525545	4.017369	3.855773	1.461258	high	1.30E-07
**TCGA-RC-A6M3**	0.00274	0	10.83543	1.356759	8.13876	3.663113	1.854576	1.413404	6.655542	4.145214	4.344454	5.096435	high	0.583187
**TCGA-DD-AAE0**	1.520548	0	9.009184	2.730066	7.848776	5.452181	1.677515	1.210141	2.482971	3.89917	2.169756	3.56547	high	3.100307
**TCGA-G3-A6UC**	1.838356	0	8.99865	1.99742	5.957524	5.179814	2.790892	2.008084	1.254358	3.652613	6.197464	1.135956	high	2.022294
**TCGA-DD-A1ED**	6.30411	0	8.850578	1.358606	6.249671	3.429937	1.551608	1.273988	1.482121	5.774979	6.191312	0.521911	low	0.420066
**TCGA-CC-5262**	0.282192	1	9.500885	2.540012	7.085042	3.615503	2.618305	1.235471	1.254358	4.397321	4.996844	1.396778	high	5.710638
**TCGA-DD-AAW0**	5.520548	0	9.855328	2.432697	5.821474	4.292986	1.820752	1.233895	2.110472	2.174841	6.265328	0.593413	low	0.181662
**TCGA-DD-AAD8**	3.339726	0	8.948472	1.822141	6.979771	5.193488	1.761049	1.406146	1.254358	3.705198	5.671036	0.941646	high	0.745699
**TCGA-RC-A7SK**	1.293151	0	8.881384	2.478255	6.418829	3.913863	1.893255	5.93107	1.480873	1.216399	5.078284	1.206726	high	0.78631
**TCGA-DD-AAC8**	0.043836	1	10.06687	3.936666	7.118544	4.313525	1.098496	1.365607	4.395	4.071127	1.52587	5.704336	high	0.996436
**TCGA-KR-A7K8**	2.482192	0	8.879334	2.330765	6.099211	5.927491	1.781566	1.325047	1.254358	3.409092	5.589659	1.000538	high	1.039729
**TCGA-CC-A1HT**	0.276712	1	9.97693	3.440625	7.942687	4.706837	3.63717	1.323131	1.426498	4.909938	1.896929	10.35519	high	2.01049
**TCGA-DD-A4NN**	2.463014	1	11.13017	2.139553	7.032306	3.671138	1.553376	1.255892	2.092981	3.456869	4.162585	1.615786	high	0.714821
**TCGA-DD-AACC**	4.616438	1	9.815001	1.474933	6.484205	3.269079	2.010828	1.26302	1.254358	2.697989	4.814277	0.480373	low	0.20637
**TCGA-DD-AADI**	2.972603	0	10.20513	2.563883	7.354245	4.059735	1.465637	1.239099	1.31704	2.09152	4.567655	0.928276	high	0.564081
**TCGA-DD-AAEB**	1.309589	0	7.768963	1.305115	5.719475	4.419705	1.06929	1.233046	1.254358	3.176675	4.124301	0.249489	low	0.202645
**TCGA-DD-AACB**	6.367123	0	9.427023	2.92369	7.663791	4.129741	1.109459	1.916325	1.671701	2.97997	3.067372	1.537531	high	0.99223
**TCGA-DD-A1EJ**	2.753425	1	10.2061	1.364131	6.981701	5.350787	1.06929	3.783464	1.322554	3.196419	3.721506	2.265984	high	1.950813
**TCGA-DD-AACW**	3.90137	0	9.822091	1.855123	6.887408	3.455807	1.327996	1.539817	1.254358	1.616262	4.578045	0.430758	low	0.194253
**TCGA-ED-A97K**	0.016438	0	9.555754	1.402542	7.84812	2.873987	3.088727	1.210141	3.326786	3.550992	5.833485	1.227386	high	1.655056
**TCGA-DD-AADW**	1.608219	0	9.626909	2.359332	7.318913	4.129769	2.370029	1.468283	1.391348	4.796891	6.667002	1.518926	high	0.64352
**TCGA-DD-A39W**	2.265753	1	10.26391	3.242669	7.637344	3.619395	1.095198	1.234259	1.254358	3.172863	4.725351	1.186424	high	1.00775
**TCGA-DD-A4NR**	0.024658	1	9.96021	2.320187	7.258282	2.360296	2.818461	1.446418	1.753823	3.760799	3.813795	1.319939	high	1.212394
**TCGA-DD-AACU**	4.293151	0	10.23955	2.769075	7.566421	2.966814	2.995724	1.238616	1.254358	2.001634	6.19431	0.919026	high	0.78631
**TCGA-DD-AACQ**	1.183562	1	8.474799	2.050875	6.754409	5.279758	1.127762	1.492229	1.254358	2.573498	6.364126	0.4681	low	0.30966
**TCGA-DD-A11B**	0.038356	1	9.428548	1.593563	5.843317	3.251519	2.698103	1.210141	1.254358	3.796305	7.052452	0.426742	low	0.256797
**TCGA-DD-A4NS**	6.728767	1	9.298434	2.483973	7.278142	4.271786	2.527585	1.372999	1.358739	3.779469	6.759702	1.14781	high	0.599266
**TCGA-DD-A1EH**	4.09589	0	10.29647	2.244404	6.720003	3.323565	1.851606	1.210141	1.383751	5.141547	6.736481	1.010284	high	1.22936
**TCGA-DD-AAW3**	4.473973	0	8.167372	2.012388	7.188172	3.723634	2.100496	1.210141	1.254358	2.977379	4.874964	0.48946	low	0.23875
**TCGA-DD-AADA**	3.378082	0	9.406762	1.717812	5.883739	1.470055	1.413973	1.210141	1.342086	2.288968	5.4009	0.140312	low	0.283585
**TCGA-DD-A119**	0.610959	1	9.208193	2.171278	6.133339	4.605857	1.490554	1.583906	4.121246	2.745163	2.665133	1.518048	high	4.921541
**TCGA-DD-A1EE**	0.956164	1	10.29117	2.770613	7.34249	4.033694	1.620549	1.30586	1.254358	5.334113	3.89592	2.605487	high	3.05687
**TCGA-CC-5263**	0.353425	1	11.27277	3.916932	8.104241	4.982593	3.72701	5.32812	1.751312	5.370804	7.05993	32.45955	high	2.097842
**TCGA-DD-AAVS**	4.994521	0	10.7941	2.09765	7.105097	2.840623	2.143168	1.254343	2.515714	3.036233	5.480104	1.073032	high	0.569798
**TCGA-DD-A4NO**	6.150685	0	9.513394	2.741459	6.678778	3.761046	2.048392	1.61886	1.254358	4.868195	5.057994	1.414553	high	1.202862
**TCGA-CC-A3MA**	0.830137	1	9.254815	3.617114	7.064204	3.219128	3.102864	1.210141	1.402498	3.532378	3.40047	1.864327	high	1.880167
**TCGA-DD-A4NE**	1.808219	1	9.249215	1.636082	7.015394	4.307283	2.812379	1.258392	1.254358	2.709871	8.660519	0.508019	low	1.006482
**TCGA-DD-AACA**	6.30411	0	8.934133	2.676101	6.317347	4.867107	1.137595	1.217047	1.254358	4.088346	4.608323	0.890306	low	0.290023
**TCGA-DD-A1EB**	5.526027	0	9.340185	3.262545	5.77664	5.334127	2.282214	1.274692	1.347592	2.696197	8.142108	0.780134	low	0.242113
**TCGA-DD-AAVU**	6.032877	0	9.158198	2.903413	5.957684	5.992956	1.264378	1.234623	1.307381	1.686487	3.923101	0.884876	low	0.220343
**TCGA-2Y-A9H0**	10.06849	0	9.104952	2.38762	6.428597	3.763791	2.863239	1.232318	1.254358	2.998473	6.603208	0.624166	low	0.895172
**TCGA-ED-A4XI**	2.243836	0	9.27111	1.667596	6.972655	3.267323	2.001715	1.231832	1.43172	2.011986	6.27818	0.335394	low	0.358246
**TCGA-CC-A3M9**	0.821918	1	10.74554	1.432955	8.477628	4.825136	1.125434	1.312934	1.254358	5.572599	2.102777	4.341296	high	1.835448
**TCGA-DD-A3A6**	8.926027	1	7.787423	3.242437	6.020852	4.100375	2.261111	1.210141	1.254358	4.469603	3.236622	0.985072	high	1.027505
**TCGA-G3-AAV4**	0.073973	1	9.837439	4.372557	7.094732	4.913824	1.06929	1.293787	1.254358	4.99756	6.191637	2.424366	high	1.603136
**TCGA-DD-A11D**	4.273973	1	9.26649	1.763631	6.197876	3.643002	2.591965	1.565772	1.254358	4.311648	7.160476	0.628346	low	0.184221

**TCGA-BD-A3EP**	1.120548	0	10.17219	3.339266	7.287337	4.323388	1.700153	1.283345	1.628999	4.314162	6.096916	1.976191	high	0.77337
**TCGA-CC-A7IF**	1.778082	1	8.156866	2.614431	5.644653	4.862734	1.329114	1.356086	1.336446	2.857295	5.372328	0.413696	low	1.074054
**TCGA-CC-A5UE**	0.745205	1	9.916622	2.23156	8.435195	6.126142	3.039868	2.459753	1.56482	3.059451	4.866275	6.071778	high	2.254603
**TCGA-CC-A8HU**	0.942466	1	9.657468	2.554252	8.012291	5.013956	2.29476	2.556808	1.313607	3.030457	3.898719	3.23397	high	1.579827
**TCGA-DD-AAD5**	3.684932	0	9.225053	2.533473	7.444825	4.926426	2.010621	1.33827	1.254358	3.672588	2.399269	2.251931	high	0.71785
**TCGA-G3-A5SI**	2.10411	1	9.677159	1.355888	7.153629	3.751931	1.128648	1.318722	1.254358	3.887814	6.894586	0.466859	low	0.21246
**TCGA-KR-A7K2**	2.271233	0	9.724033	1.659475	7.200103	2.645184	2.419267	1.329096	1.307938	3.015711	4.821529	0.612064	low	0.390544
**TCGA-DD-AADM**	0.032877	1	9.111836	1.914473	6.843343	4.388533	1.525777	1.33905	1.303026	2.091715	3.428942	0.641085	low	0.358246
**TCGA-DD-AADJ**	2.920548	0	9.027714	1.464722	6.07091	4.806386	2.300719	1.210141	1.380824	3.46811	5.188407	0.70911	low	0.305022
**TCGA-ED-A7XP**	1.09589	0	9.964654	3.327842	6.486971	3.4014	1.885425	2.127107	1.964312	3.167897	4.729907	1.389699	high	0.949716
**TCGA-G3-A3CH**	2.136986	0	8.624415	1.614259	6.279286	5.315119	1.924953	1.210141	1.254358	4.160325	6.225061	0.712233	low	0.537362
**TCGA-CC-A5UD**	0.832877	1	10.56705	2.419466	7.409984	5.467956	1.236977	2.146446	2.00056	3.88978	4.573215	3.415113	high	1.404769
**TCGA-ED-A8O5**	1.112329	0	9.764127	1.894121	6.934223	3.719057	1.689607	1.277622	1.351677	5.201934	6.151512	0.981388	high	2.274305
**TCGA-DD-A116**	4.443836	1	9.810032	2.654017	5.641023	5.617629	1.09052	1.510496	2.050371	2.580971	4.375122	1.130065	high	2.790726
**TCGA-DD-A4NK**	3.315068	1	9.181743	2.080394	6.143461	4.20932	1.940319	1.261479	1.60065	3.208021	6.715693	0.519005	low	0.84378
**TCGA-DD-AAE2**	1.747945	0	9.219688	1.516947	6.339922	5.482705	2.05919	1.324146	1.254358	3.681951	9.026018	0.57665	low	0.224732
**TCGA-5R-AA1D**	1.230137	0	9.368713	1.897828	7.031457	2.983853	1.639825	1.288345	1.722227	4.394224	6.044255	0.605104	low	0.349875
**TCGA-CC-5264**	0.279452	1	10.12702	2.917554	8.268939	4.743568	3.59218	1.34372	1.297755	3.767689	8.012599	3.06529	high	2.25588
**TCGA-DD-A3A8**	0.030137	1	8.892219	1.744939	6.447754	4.099732	1.138454	1.253627	1.254358	3.944358	5.806194	0.437684	low	0.373721
**TCGA-DD-AACZ**	0.468493	1	9.649521	3.282215	8.318903	2.261839	2.01781	1.391179	1.254358	4.906438	3.898052	1.805747	high	1.428579
**TCGA-G3-A25Z**	1.794521	0	9.971494	1.588446	6.672635	4.729857	1.18365	1.507098	1.386879	2.912217	4.221859	0.863446	low	0.195394
**TCGA-5R-AAAM**	0.126027	1	9.610247	1.937807	6.456481	4.062086	1.326036	1.36102	1.606531	4.07339	6.65882	0.621933	low	0.299066
**TCGA-DD-A3A7**	1.147945	1	8.81635	2.919219	6.477378	4.425785	1.799615	1.210141	1.254358	1.894011	3.510015	0.701124	low	0.90609
**TCGA-DD-AAD6**	1.841096	0	8.418928	1.562995	5.403789	4.549142	1.10495	1.210141	1.254358	3.175601	2.371283	0.418421	low	0.889732
**TCGA-DD-AADC**	1.164384	1	10.4109	2.884538	8.044401	5.666358	2.262449	2.504599	1.254358	3.603802	5.32511	5.210784	high	0.704891
**TCGA-5C-AAPD**	0.054795	0	9.280642	1.597677	8.084762	4.49112	1.35591	1.418533	1.308495	2.805489	6.614904	0.645949	low	0.51888
**TCGA-DD-AAED**	2.090411	0	8.420955	1.40076	7.961449	3.112403	1.473975	1.239824	1.254358	2.270152	2.730394	0.42163	low	0.224732
**TCGA-DD-AACI**	4.432877	0	9.484907	1.905416	6.550996	2.283668	2.633202	1.210141	1.254358	2.052696	4.627996	0.375821	low	0.437736
**TCGA-G3-A25Y**	1.238356	1	10.1853	2.349048	7.845162	4.192051	2.460706	1.339718	2.180462	5.628329	6.290682	3.259397	high	0.664583
**TCGA-PD-A5DF**	1.750685	1	10.06563	1.892018	6.658357	5.70838	1.95407	1.258511	4.57702	4.535852	7.356861	2.891225	high	2.59734
**TCGA-DD-AAVY**	5.39726	0	9.526861	2.371617	6.647469	4.490081	1.116524	1.254223	1.254358	3.54306	4.116747	0.902689	high	1.716471
**TCGA-CC-A123**	0.6	0	9.694427	1.265571	7.16535	4.419192	1.118088	1.210141	1.931284	2.855956	3.228365	0.850872	low	0.545063
**TCGA-DD-AADG**	3.136986	0	8.976701	1.46512	6.283999	4.57808	1.229492	1.232803	1.303473	3.885138	5.06273	0.533813	low	0.95699
**TCGA-CC-A7IG**	0.819178	1	10.00586	3.580072	7.492637	5.457861	1.871594	1.601957	1.390622	3.031983	3.485933	3.652139	high	0.67865
**TCGA-DD-A3A3**	1.465753	1	8.985528	1.686089	6.297879	5.05615	1.496862	1.747963	4.464496	2.396476	1.967954	1.697297	high	0.466326
**TCGA-DD-A4NJ**	2.542466	0	9.784838	4.503083	6.919781	4.406405	1.151656	1.287184	1.254358	4.375901	5.604053	1.901922	high	0.740234
**TCGA-DD-AACS**	4.942466	0	9.341574	2.239135	7.479125	5.728667	1.192691	1.249921	1.339812	3.397855	6.060993	1.179025	high	0.546204
**TCGA-CC-A9FW**	0.679452	0	10.57305	2.935571	6.872644	4.738089	1.998941	1.29159	1.299664	2.374594	4.807104	1.61695	high	2.135896
**TCGA-G3-AAUZ**	1.315068	0	8.776003	1.66312	6.245086	5.146838	1.06929	1.375164	1.254358	2.403508	5.553667	0.397908	low	0.162851
**TCGA-G3-A25U**	4.482192	0	9.441638	1.692467	6.829822	5.263876	1.06929	1.210141	1.254358	3.444987	8.139559	0.519558	low	0.268502
**TCGA-DD-AAEK**	2.923288	0	9.017794	1.905971	6.193602	4.318359	1.117977	1.210141	1.254358	3.050378	6.412763	0.346581	low	0.315353
**TCGA-DD-AAEH**	2.147945	0	8.680015	1.888505	6.55333	4.667545	1.095426	1.258511	1.254358	2.623297	5.504808	0.394887	low	0.256797
**TCGA-DD-AACG**	1.284932	1	8.964604	1.577422	7.380294	5.65053	1.481109	2.369665	1.8108	2.215585	3.903406	1.330494	high	1.708702
**TCGA-DD-AAD2**	1.80274	0	9.713857	1.531076	6.83977	4.110283	1.767524	1.302079	4.206047	3.231192	5.818519	1.129668	high	0.68366
**TCGA-DD-AAEI**	4.194521	0	9.480324	1.300796	6.493954	4.980534	1.115517	1.709439	1.254358	2.996788	4.581542	0.678116	low	0.252161
**TCGA-G3-AAV5**	0.969863	0	10.48521	2.776613	6.757132	4.123221	1.06929	1.413194	1.254358	3.430751	3.581882	1.37107	high	0.976979
**TCGA-DD-AADK**	2.873973	0	9.271096	1.947432	5.634676	4.016837	1.756863	1.234502	3.200644	2.442124	5.04581	0.568485	low	0.412705

**TCGA-DD-AADV**	1.572603	0	9.440276	2.368059	7.265232	4.554724	2.235435	1.477877	1.254358	2.849057	6.342299	0.985814	high	0.6674
**TCGA-DD-AADR**	5.556164	0	9.149402	1.750388	5.502931	5.180864	1.965127	1.210141	1.254358	3.351261	4.715849	0.709958	low	0.255298
**TCGA-DD-AACJ**	5.758904	0	9.755311	2.735037	5.79911	4.972612	1.54063	1.629738	1.254358	2.450872	3.537168	1.066385	high	1.130266
**TCGA-DD-A113**	6.643836	0	9.455308	2.378609	7.139073	2.641806	2.159397	2.352462	1.254358	3.995102	6.172222	0.82053	low	0.325675
**TCGA-DD-A1EC**	1.649315	0	10.36965	1.626789	7.192657	2.467166	3.410288	1.228548	1.634402	4.27106	3.721938	1.645613	high	0.370994
**TCGA-DD-A4NH**	2.512329	0	9.421266	1.82459	6.784516	4.46648	3.111401	1.259224	1.811523	4.760366	5.493911	1.840181	high	2.537
**TCGA-CC-A7IE**	0.594521	1	9.246618	1.457934	7.05565	4.582677	1.303402	6.566301	5.089001	5.042268	1.65921	12.63692	high	1.748029
**TCGA-DD-A4NQ**	1.021918	1	9.583039	2.03244	7.347387	4.702752	2.404047	1.425927	1.254358	3.506386	3.599454	1.862979	high	1.123669
**TCGA-DD-AAW2**	5.082192	0	9.052821	1.863698	6.596852	3.675188	1.66376	1.245724	1.403114	3.408286	6.048272	0.445477	low	0.23875
**TCGA-DD-A73B**	0.775342	1	10.68192	1.360233	5.849738	4.342047	1.877251	1.210141	1.295505	3.674307	7.901232	0.582453	low	0.242113
**TCGA-MI-A75G**	1.912329	0	9.198685	1.485548	6.343518	5.884584	1.504739	1.36408	1.254358	4.128156	4.636542	1.167161	high	0.908317
**TCGA-CC-A3MC**	0.994521	0	9.118643	2.023653	6.534732	4.593852	2.408848	2.940448	1.801932	2.271755	4.296245	1.321673	high	1.655056
**TCGA-DD-A73G**	9.528767	0	8.538708	1.789999	7.251763	3.565339	1.176663	1.348814	2.684168	2.091178	3.600388	0.489085	low	0.322156
**TCGA-CC-5260**	0.238356	1	10.1969	4.187115	8.284475	4.599618	3.572609	1.210141	1.254358	4.400852	4.236787	8.38105	high	4.915323
**TCGA-DD-A4NL**	4.687671	0	8.612078	1.38694	6.136057	3.509795	1.879053	1.59835	1.254358	2.934076	7.175157	0.239659	low	0.281355
**TCGA-DD-A3A1**	0.638356	1	9.33438	1.848088	5.6892	5.624479	1.889231	1.210141	1.254358	3.159282	5.743198	0.760145	low	0.668668
**TCGA-G3-AAV2**	1.019178	0	9.997642	1.466811	6.911103	4.97833	1.300446	1.233774	1.49025	2.722197	4.864575	0.841333	low	0.302623
**TCGA-DD-A4NV**	6.569863	0	8.733149	1.708226	6.188104	5.250776	1.529547	1.231832	1.390414	3.701632	9.192045	0.377571	low	1.113909
**TCGA-BC-A3KG**	1.863014	0	8.786535	1.655139	8.216681	5.459752	1.867378	1.23086	1.254358	3.561603	6.08228	1.172809	high	1.248169
**TCGA-G3-AAV6**	0.178082	1	10.83993	1.490334	7.078279	5.661127	1.120208	7.299766	1.403833	2.455095	2.349027	8.313644	high	2.038811
**TCGA-DD-AAVZ**	5.205479	0	10.4767	1.587186	7.150123	2.375815	2.448268	1.238133	1.314826	2.712205	4.904703	0.630487	low	0.135729
**TCGA-CC-A9FU**	0.00274	0	9.861575	2.396008	7.503432	5.748302	1.279153	5.324747	1.468511	2.408991	2.882155	5.363565	high	1.532244
**TCGA-DD-AACE**	5.983562	0	8.007582	1.87684	6.005705	5.071311	1.06929	1.23438	1.254358	3.107168	6.248179	0.308973	low	0.274597
**TCGA-DD-A3A5**	8.561644	1	8.941724	1.411725	6.849569	5.57817	1.845704	1.261716	1.254358	2.295522	5.338605	0.677682	low	0.653825
**TCGA-RC-A7SF**	1.586301	0	8.863594	2.325414	6.374835	4.173927	2.194165	1.359158	1.394663	3.044735	4.911876	0.705553	low	0.226051
**TCGA-ED-A7XO**	1.169863	0	9.703478	1.572966	7.222473	5.229751	1.344659	1.44652	1.316155	2.996577	5.447655	0.944633	high	1.049575
**TCGA-DD-A73A**	1.994521	0	9.144501	1.781657	6.248951	3.75619	1.465057	1.530597	1.306712	2.73205	6.165656	0.344081	low	0.247609
**TCGA-DD-AAVV**	6.726027	0	9.550883	1.762071	6.874515	3.880046	2.134524	1.313844	1.470548	3.499673	4.578359	0.907619	high	1.044698
**TCGA-DD-AAVQ**	7.473973	0	9.864714	1.938617	6.825018	5.27706	2.26132	1.34372	1.419127	1.943092	3.822821	1.338319	high	0.410493
**TCGA-G3-AAV1**	0.983562	1	10.01502	2.579048	6.105452	3.805858	1.714893	1.251715	1.343708	4.374867	4.161093	1.169755	high	3.111308
**TCGA-G3-A25T**	4.254795	0	9.394065	1.951115	7.352633	4.534665	2.770002	1.210141	3.770344	3.500265	5.596535	2.104396	high	1.34879
**TCGA-DD-AACF**	1	1	9.438521	1.325366	6.88444	3.954423	1.311941	1.512627	1.254358	2.549491	5.545335	0.416008	low	0.306315
**TCGA-DD-AAC9**	0.950685	0	9.251634	1.659136	5.971039	3.338953	1.623664	1.210141	1.400338	2.150002	3.402559	0.358062	low	0.172
**TCGA-DD-AACK**	0.024658	0	9.766329	2.301464	6.966375	4.499887	2.235221	1.295981	1.254358	4.405436	6.27356	1.387237	high	0.73534
**TCGA-G3-A25V**	2.356164	0	8.952906	1.498108	6.700778	3.973361	1.684332	1.390432	1.254358	3.002211	6.600552	0.373545	low	0.234439
**TCGA-DD-AAD3**	3.547945	0	9.3242	2.217075	6.746872	4.120063	2.062023	1.258629	1.254358	4.025666	5.529917	0.916748	high	0.44964
**TCGA-RC-A7SB**	1.610959	0	9.402213	1.802972	5.832736	5.030172	1.254786	1.715451	1.320903	2.869823	5.254069	0.604618	low	0.250313
**TCGA-UB-A7MA**	2.323288	0	9.251729	3.687408	7.45669	2.763165	2.732432	1.241633	2.141256	3.669836	5.755742	1.358591	high	0.856376
**TCGA-DD-A1EG**	3.758904	1	9.68238	1.971861	6.908931	3.839714	1.57733	1.280078	3.121962	3.079001	2.229487	1.453329	high	1.071759
**TCGA-DD-AADF**	0.315068	1	8.337265	1.772484	6.139549	5.072202	1.682958	1.29817	1.254358	2.65266	4.51889	0.500393	low	0.481041
**TCGA-RC-A6M5**	0.041096	0	9.110639	2.651608	7.990975	2.775363	1.407024	1.258986	1.391659	4.419609	5.059034	0.831006	low	2.61E-07
**TCGA-DD-AACP**	1.136986	0	10.15498	1.738671	7.496205	5.017171	1.246015	8.660976	1.63869	3.274269	2.95588	10.85706	high	0.861298
**TCGA-BC-A8YO**	1.539726	0	10.46078	3.095345	7.606954	4.819905	2.847088	1.693487	1.309497	3.055012	6.262759	2.864026	high	3.926102
**TCGA-DD-A3A9**	2.550685	1	9.331795	2.058613	6.408335	2.633927	1.311752	1.234986	2.085853	3.044713	5.168488	0.363039	low	2.813751
**TCGA-CC-A7II**	1.093151	0	10.43197	1.339785	8.639124	5.740088	1.33237	10.35362	2.507654	3.968255	1.426845	51.0995	high	2.162468
**TCGA-DD-AAD1**	1.545205	0	10.18343	2.0416	7.21595	3.698191	3.634832	1.210141	1.254358	3.626869	5.818437	1.663568	high	1.14805
**TCGA-DD-AAVR**	6.884932	0	10.42458	1.731731	6.735253	2.549445	1.576125	1.210141	1.512247	3.526934	4.872551	0.580251	low	0.151406

**TCGA-DD-AAE3**	1.550685	0	10.18096	1.573603	5.321626	4.807389	1.490716	1.237649	1.37052	4.437787	5.407001	0.832238	low	0.168894
**TCGA-ED-A66X**	1.112329	0	10.38965	2.012325	7.111326	4.000872	2.558423	1.210141	3.263545	3.336321	2.66119	2.924121	high	1.529757
**TCGA-G3-A3CK**	1.60274	0	9.654886	1.774339	6.576888	4.800592	1.312131	1.539438	1.36703	4.510007	4.59202	1.202604	high	0.624138
**TCGA-DD-AAVP**	7.539726	0	8.061431	1.416083	6.951485	4.752525	1.310239	1.210141	1.342194	3.796942	3.818909	0.594335	low	0.291796
**TCGA-KR-A7K7**	2.605479	0	9.386169	2.285758	7.040381	4.39956	2.773536	1.27046	2.291703	3.956542	2.428886	2.753895	high	1.506745
**TCGA-DD-A1EL**	1.136986	1	9.67613	2.437061	7.540285	5.298064	1.28032	1.646529	1.254358	2.60814	2.538572	1.892934	high	0.572659
**TCGA-G3-A3CI**	0.493151	0	8.183763	2.816821	6.00789	3.4036	1.687075	1.210141	1.345867	3.903939	6.342388	0.361835	low	0.247609
**TCGA-ED-A5KG**	2.339726	0	10.4272	1.787403	8.131232	2.260937	3.093997	1.749562	1.322994	5.49717	4.259491	2.538071	high	0.825735
**TCGA-5C-A9VH**	0.882192	0	10.60803	1.961167	6.851629	3.26294	2.523117	1.292516	1.254358	3.70007	3.144226	1.557931	high	0.73534
**TCGA-DD-AADU**	1.517808	0	9.790804	2.371096	5.174935	4.514133	1.133066	1.326624	1.298204	3.524142	2.556986	0.864498	low	0.371539
**TCGA-FV-A2QR**	1.591781	1	10.16245	4.388419	6.700164	5.453146	1.175494	1.28544	1.362786	3.976317	7.972971	1.854515	high	1.443229
**TCGA-KR-A7K0**	0.178082	1	9.214282	2.43724	6.495324	4.444015	1.459907	1.359925	1.254358	3.344264	3.850965	0.891005	high	1.202862
**TCGA-DD-AADO**	1.241096	0	9.009096	1.713029	6.32579	3.970128	1.423288	1.379376	4.170003	2.88866	2.806865	1.005089	high	0.731048
**TCGA-RG-A7D4**	3.008219	0	9.743198	3.24074	6.875143	3.656096	2.03962	1.783768	1.864154	3.171847	1.944094	2.184155	high	1.013232
**TCGA-DD-AACD**	1.043836	1	8.733315	1.477497	6.049178	4.2848	1.882765	1.878604	3.466904	2.961089	3.88739	0.882577	low	0.374328
**TCGA-DD-AAD0**	0.375342	0	10.04772	1.663289	5.966813	5.215107	1.317129	1.210141	1.309497	3.115196	5.764415	0.707998	low	0.246564
**TCGA-G3-A25S**	1.139726	1	10.12357	1.513317	7.003044	5.345303	1.427148	1.210141	1.254358	2.360429	4.542561	0.982392	high	0.659199
**TCGA-DD-AACX**	0.465753	0	8.215351	2.943695	7.114738	5.6929	2.060782	1.377542	1.254358	2.931263	3.901999	1.465074	high	1.253459
**TCGA-MI-A75E**	1.389041	0	8.931709	2.116536	6.739255	4.477368	1.502413	2.01074	1.34414	3.571976	5.392185	0.815189	low	0.944695
**TCGA-DD-AACO**	5.139726	0	9.438475	1.327929	5.848663	4.395647	1.669407	1.246204	1.254358	3.205689	5.776241	0.439066	low	0.183922
**TCGA-CC-A9FS**	0.578082	0	9.487473	1.443535	6.005181	4.704793	1.546518	1.466576	1.839267	1.619129	6.286016	0.380951	low	0.259602
**TCGA-XR-A8TD**	2.821918	0	8.962333	3.458385	6.396391	5.66729	1.915084	1.341832	1.67626	5.79832	6.04058	2.707099	high	1.895523
**TCGA-5R-AA1C**	1.424658	0	9.799362	1.597052	6.985699	4.42483	1.296046	1.471889	1.375789	3.691204	4.902777	0.901428	high	0.814265
**TCGA-DD-AAE9**	1.978082	0	9.230737	1.958379	6.428399	4.856377	2.548202	1.284975	1.254358	2.209745	4.553087	0.850942	low	0.311946
**TCGA-CC-A7IH**	1	0	9.882396	2.092779	6.659365	4.297323	1.195724	1.419368	1.29899	2.644489	5.576574	0.615117	low	1.054664
**TCGA-DD-AACL**	0.293151	1	9.733034	1.394871	8.066812	4.211632	1.829573	2.191807	2.027446	3.035938	2.980359	1.896802	high	0.857663
**TCGA-CC-5259**	0.684932	0	9.421794	1.264985	6.239345	3.020686	2.250816	1.818188	1.254358	4.095731	2.65695	0.821357	low	0.890674
**TCGA-DD-AAVX**	4.706849	0	9.876677	1.638761	6.380376	3.783417	2.320208	1.210141	1.254358	3.06722	4.407279	0.758974	low	0.19046
**TCGA-CC-A8HT**	0.383562	1	8.640307	1.915161	7.454656	4.782391	2.511298	2.238525	1.354999	3.713702	3.703503	1.854093	high	2.382604
**TCGA-DD-A73F**	2.972603	0	9.367411	1.445357	6.724552	4.937932	2.384531	1.272225	1.300001	2.591136	6.801392	0.644162	low	0.475187
**TCGA-DD-A1EF**	1.079452	1	9.833235	1.739784	7.553796	3.914179	1.194366	1.2504	1.567945	4.374054	2.745095	1.43791	high	0.727963
**TCGA-DD-AADQ**	1.194521	0	9.038353	1.630706	6.349267	3.938705	1.680825	1.315661	1.254358	3.146069	5.363877	0.4376	low	0.364832
**TCGA-DD-A1EK**	1.528767	1	8.592322	1.45322	5.980395	4.995575	1.145674	1.474088	1.254358	5.196802	5.826141	0.626826	low	2.813751
**TCGA-DD-A11C**	1.813699	0	9.188229	1.693292	7.231853	6.353696	2.845975	1.346823	1.331106	5.420414	6.155622	3.207133	high	0.943438
**TCGA-CC-A7IL**	0.761644	1	9.232198	2.431705	5.568373	5.585878	1.477424	1.357952	1.254358	2.602767	4.199373	0.819134	low	1.113909
**TCGA-DD-A115**	6.964384	1	9.023223	1.40474	6.184063	6.551028	2.318696	1.37289	1.254358	2.784102	5.886759	1.015348	high	1.625177
**TCGA-BC-A69I**	1.060274	0	8.409492	1.660663	6.403893	5.005374	1.094514	1.210141	1.254358	5.013852	5.226189	0.676582	low	0.427789
**TCGA-MI-A75C**	0.79726	0	8.970429	1.442218	6.981665	4.039542	2.383477	1.994065	1.297193	3.545974	2.870409	1.196482	high	0.861298
**TCGA-DD-AACV**	4.194521	0	9.621187	1.612761	6.910153	5.67159	1.104386	1.274809	1.254358	3.801354	7.489469	0.783718	low	0.233071
**TCGA-LG-A9QD**	1.00274	0	9.004267	1.854689	5.943728	3.401899	1.985926	1.228304	1.254358	2.177939	5.693462	0.279427	low	0.70623
**TCGA-DD-AAVW**	6.347945	0	10.91142	1.79373	6.48963	3.623308	2.285087	1.440378	1.57603	3.54668	4.365561	1.384659	high	0.38866
**TCGA-CC-A7IK**	0.717808	1	10.38443	2.683914	7.658786	4.830106	1.06929	1.27809	1.254358	3.246229	1.748492	2.541613	high	2.368697
**TCGA-RC-A7S9**	1.753425	0	8.828561	1.69766	7.790181	3.250611	1.370834	1.301045	1.254358	3.240206	4.735316	0.488785	low	0.200611
**TCGA-DD-AADD**	3.372603	0	9.024093	2.481291	8.007883	5.560245	1.10201	1.240668	1.254358	3.45346	2.681031	1.96352	high	1.195698
**TCGA-DD-AACN**	3.567123	0	9.382378	2.578288	6.89669	3.76334	2.655657	1.210141	1.504929	4.398697	5.900464	1.254774	high	0.480807
**TCGA-DD-AADY**	1.520548	0	10.28767	1.75149	6.852874	2.826755	2.772081	1.254343	1.254358	4.324223	7.310621	0.77053	low	0.177629
**TCGA-DD-A4NP**	9.063014	0	8.737478	1.399815	6.14999	4.854576	1.551992	1.224399	1.285325	1.999059	5.991867	0.310498	low	0.158978

**TCGA-DD-AACY**	3.972603	0	9.551515	2.160625	7.248691	2.271897	2.465981	1.210141	1.254358	4.039935	5.858156	0.640561	low	0.26964
**TCGA-CC-A5UC**	0.950685	1	9.725753	1.312805	6.772819	4.407379	1.592659	1.264914	3.055958	3.095156	3.500148	1.177629	high	2.547753
**TCGA-CC-A8HV**	0.764384	1	9.272713	2.646739	7.797036	4.809109	2.173601	1.210141	1.387608	2.969453	4.158103	1.700525	high	0.700863

Supplementary Table S8. The HCC patients' tumor mutation burden (TMB) results.

id	TMB
TCGA-2Y-A9GV	1.447368
TCGA-5C-A9VG	2.815789
TCGA-G3-A25U	1.184211
TCGA-G3-A5SK	1.868421
TCGA-DD-A1EA	1.921053
TCGA-ZS-A9CD	2
TCGA-DD-AAC8	11.81579
TCGA-T1-A6J8	1.763158
TCGA-RC-A6M6	4.157895
TCGA-DD-A1ED	0.421053
TCGA-5R-AA1C	1.894737
TCGA-DD-AACC	1.868421
TCGA-DD-A4NK	1.552632
TCGA-2Y-A9H2	1.052632
TCGA-K7-A5RG	2.657895
TCGA-DD-A4NL	0.552632
TCGA-2Y-A9H8	1.5
TCGA-BW-A5NP	1.552632
TCGA-DD-AACN	1.026316
TCGA-DD-AACQ	4.5
TCGA-DD-A3A4	0.684211
TCGA-DD-A1E9	1.289474
TCGA-2Y-A9GZ	1.973684
TCGA-DD-A39W	0.736842
TCGA-G3-A5SL	2.947368
TCGA-DD-AAW1	2.447368
TCGA-CC-A7IF	2.684211
TCGA-DD-A4NP	0.473684
TCGA-DD-A4NG	1.552632
TCGA-DD-A113	3.236842
TCGA-EP-A3JL	1.921053
TCGA-WX-AA47	0.842105
TCGA-DD-AAEE	1.631579
TCGA-CC-A9FU	0.973684
TCGA-DD-A116	2.421053
TCGA-2Y-A9H1	2.763158
TCGA-ED-A7PX	0.710526
TCGA-DD-A73F	1.315789
TCGA-RC-A6M3	1.605263
TCGA-BW-A5NO	2.447368
TCGA-BC-A69H	1.657895
TCGA-RC-A7SK	2.789474
TCGA-DD-A4NO	1.210526
TCGA-G3-A6UC	2.473684
TCGA-DD-A4NR	1.052632
TCGA-DD-AACJ	2.052632
TCGA-DD-AAW2	2.5
TCGA-GJ-A9DB	1.552632
TCGA-DD-AACX	2.631579
TCGA-QA-A7B7	1.815789
TCGA-ZP-A9D4	1.263158
TCGA-DD-AAEG	2.289474
TCGA-FV-A495	2.236842
TCGA-G3-A25Z	1.552632

**TCGA-G3-AAUZ**	1.710526
**TCGA-EP-A2KB**	1.789474
**TCGA-DD-AAVW**	0.710526
**TCGA-LG-A9QC**	1.026316
**TCGA-DD-A11A**	2.736842
**TCGA-DD-A73B**	1.710526
**TCGA-DD-A11D**	2.605263
**TCGA-FV-A3I0**	1.815789
**TCGA-DD-A3A5**	1.263158
**TCGA-FV-A4ZP**	1.789474
**TCGA-CC-A3MC**	2.157895
**TCGA-DD-AAEK**	1.684211
**TCGA-G3-AAV2**	1.026316
**TCGA-4R-AA8I**	24.94737
**TCGA-BC-A10Y**	1.184211
**TCGA-2Y-A9GU**	3.052632
**TCGA-DD-A119**	1.342105
**TCGA-G3-A25V**	1.052632
**TCGA-UB-A7MA**	1.710526
**TCGA-DD-AAD1**	2.684211
**TCGA-UB-A7MD**	2.368421
**TCGA-DD-AADJ**	1.342105
**TCGA-WX-AA46**	1.263158
**TCGA-DD-A4NV**	2.631579
**TCGA-DD-AAD8**	2.394737
**TCGA-DD-AAE4**	1.315789
**TCGA-G3-A7M8**	0.473684
**TCGA-DD-AADC**	1.815789
**TCGA-XR-A8TF**	2.947368
**TCGA-CC-A7IH**	15.26316
**TCGA-ZP-A9CZ**	1.078947
**TCGA-CC-5260**	1.157895
**TCGA-CC-A7II**	3.578947
**TCGA-ZP-A9CV**	2.210526
**TCGA-G3-AAV1**	1.131579
**TCGA-DD-AAVS**	1.157895
**TCGA-DD-AADN**	2.368421
**TCGA-DD-AADQ**	2.552632
**TCGA-CC-A9FV**	0.263158
**TCGA-DD-A4NE**	1.210526
**TCGA-DD-AAD2**	1.789474
**TCGA-CC-A5UC**	1.131579
**TCGA-ED-A8O6**	1.5
**TCGA-BC-A10Q**	0.736842
**TCGA-2Y-A9H9**	2.763158
**TCGA-BC-4073**	2.868421
**TCGA-DD-AACA**	8.157895
**TCGA-DD-AACD**	1.789474
**TCGA-DD-AAD6**	2.131579
**TCGA-G3-A7M6**	1.947368
**TCGA-ED-A5KG**	0.289474
**TCGA-5R-AA1D**	0.263158
**TCGA-DD-A1EC**	0.5
**TCGA-DD-A39Z**	1.5
**TCGA-BD-A3EP**	2.184211
**TCGA-2Y-A9GW**	1.710526
**TCGA-BC-A217**	2.263158

**TCGA-RC-A7SH**	1.447368
**TCGA-2Y-A9HA**	3.052632
**TCGA-DD-A39V**	1.473684
**TCGA-ED-A459**	5.105263
**TCGA-DD-AAC9**	1.815789
**TCGA-ED-A4XI**	3
**TCGA-G3-AAV3**	1.921053
**TCGA-DD-AAVR**	1.315789
**TCGA-DD-AAVU**	1.105263
**TCGA-DD-A73E**	2.157895
**TCGA-GJ-A6C0**	1
**TCGA-RC-A7SB**	1.131579
**TCGA-ZP-A9D0**	1.157895
**TCGA-DD-A4NH**	1.078947
**TCGA-DD-AADY**	0.973684
**TCGA-CC-A1HT**	1.815789
**TCGA-BC-A112**	24.02632
**TCGA-BC-A8YO**	1.5
**TCGA-XR-A8TG**	1.710526
**TCGA-BC-A10X**	0.026316
**TCGA-BD-A3ER**	1.131579
**TCGA-DD-A4NB**	0.552632
**TCGA-CC-5264**	1.973684
**TCGA-G3-AAV6**	1.394737
**TCGA-2Y-A9GT**	1.868421
**TCGA-2Y-A9H7**	1.710526
**TCGA-G3-A5SI**	0.605263
**TCGA-DD-AAD3**	1.394737
**TCGA-DD-AA3A**	0.868421
**TCGA-2Y-A9GS**	1.526316
**TCGA-CC-A7IE**	3.447368
**TCGA-DD-AADM**	4.131579
**TCGA-WQ-AB4B**	1.5
**TCGA-DD-AAE0**	1.447368
**TCGA-DD-A4NI**	2.947368
**TCGA-5C-A9VH**	2.289474
**TCGA-DD-AAVP**	1.552632
**TCGA-MR-A520**	0.368421
**TCGA-DD-A1EJ**	1.263158
**TCGA-WJ-A86L**	2.763158
**TCGA-G3-A3CJ**	9.236842
**TCGA-DD-AACY**	1.710526
**TCGA-XR-A8TE**	0.263158
**TCGA-LG-A9QD**	2.184211
**TCGA-DD-AAVV**	2.631579
**TCGA-CC-A7IK**	6.868421
**TCGA-DD-A11C**	1.973684
**TCGA-2Y-A9H3**	3.473684
**TCGA-DD-A3A0**	8.157895
**TCGA-DD-A118**	2.5
**TCGA-CC-A9FS**	2.315789
**TCGA-HP-A5MZ**	1.078947
**TCGA-EP-A3RK**	1.289474
**TCGA-ED-A7PY**	0.526316
**TCGA-BC-A216**	1.210526
**TCGA-CC-A3M9**	2.684211
**TCGA-UB-A7MC**	1.578947

**TCGA-MI-A75C**	2.5
**TCGA-DD-A4NS**	0.947368
**TCGA-CC-A7IG**	3
**TCGA-G3-A25W**	1.684211
**TCGA-DD-AACM**	1.184211
**TCGA-DD-A4NF**	2.552632
**TCGA-K7-A5RF**	1.052632
**TCGA-DD-AAVZ**	0.868421
**TCGA-BC-A5W4**	1.394737
**TCGA-CC-A3MA**	0.868421
**TCGA-CC-A7IL**	2.184211
**TCGA-DD-AAEI**	2.526316
**TCGA-EP-A26S**	1.947368
**TCGA-RC-A7SF**	1.736842
**TCGA-DD-AADD**	1.763158
**TCGA-DD-AADA**	2.684211
**TCGA-KR-A7K0**	2.026316
**TCGA-G3-A25S**	2.552632
**TCGA-G3-A25T**	0.815789
**TCGA-G3-AAV7**	1.710526
**TCGA-CC-A5UD**	4.052632
**TCGA-2Y-A9H6**	1.078947
**TCGA-DD-AAEB**	2.657895
**TCGA-CC-A9FW**	3.105263
**TCGA-DD-AAW3**	2.078947
**TCGA-FV-A2QQ**	2.684211
**TCGA-O8-A75V**	1.605263
**TCGA-DD-AAEH**	1.947368
**TCGA-G3-A7M9**	2.5
**TCGA-DD-A73G**	2.368421
**TCGA-3K-AAZ8**	2.842105
**TCGA-FV-A3I1**	1.5
**TCGA-ZS-A9CG**	1.684211
**TCGA-DD-A1EG**	10.47368
**TCGA-MR-A8JO**	0.684211
**TCGA-NI-A8LF**	2.131579
**TCGA-DD-AAVX**	1.473684
**TCGA-ED-A7XP**	1.394737
**TCGA-DD-A1EL**	1.947368
**TCGA-DD-AAE3**	3.526316
**TCGA-RC-A6M5**	0.447368
**TCGA-DD-A1EK**	1.184211
**TCGA-DD-AADW**	0.973684
**TCGA-5C-AAPD**	1.394737
**TCGA-CC-A8HV**	3.026316
**TCGA-DD-AAD0**	2.236842
**TCGA-CC-5259**	3.131579
**TCGA-DD-A3A7**	2.578947
**TCGA-CC-5262**	2.657895
**TCGA-RG-A7D4**	2
**TCGA-DD-A4NA**	0.657895
**TCGA-DD-A4ND**	1.263158
**TCGA-DD-AACI**	7.631579
**TCGA-2Y-A9HB**	1.5
**TCGA-DD-AAE6**	1.657895
**TCGA-ED-A7XO**	1.263158
**TCGA-DD-A3A9**	7.684211

**TCGA-BC-A3KG**	4.026316
**TCGA-DD-AADF**	4.710526
**TCGA-DD-AAVQ**	1.210526
**TCGA-DD-AADS**	3.052632
**TCGA-WX-AA44**	1.605263
**TCGA-XR-A8TC**	1.131579
**TCGA-K7-A6G5**	1.5
**TCGA-BC-A10U**	3.078947
**TCGA-KR-A7K7**	1.394737
**TCGA-DD-AACB**	2.210526
**TCGA-BC-A10R**	2.5
**TCGA-DD-AACH**	1.973684
**TCGA-UB-A7ME**	1.842105
**TCGA-ED-A82E**	0.578947
**TCGA-RC-A7S9**	1.236842
**TCGA-2Y-A9GY**	1.447368
**TCGA-MI-A75E**	2.605263
**TCGA-DD-A115**	1.736842
**TCGA-G3-AAV0**	3.789474
**TCGA-FV-A2QR**	2.368421
**TCGA-DD-AADI**	2.236842
**TCGA-DD-AAEA**	4
**TCGA-DD-AAED**	1.394737
**TCGA-G3-A5SM**	2.315789
**TCGA-ES-A2HT**	1.552632
**TCGA-ES-A2HS**	1.394737
**TCGA-ZP-A9CY**	1.763158
**TCGA-RC-A6M4**	3.631579
**TCGA-DD-AACT**	4.026316
**TCGA-DD-AACS**	1.157895
**TCGA-DD-AACF**	2.736842
**TCGA-CC-A8HU**	1.921053
**TCGA-DD-AAW0**	2.026316
**TCGA-FV-A3R3**	1.131579
**TCGA-FV-A496**	2.5
**TCGA-PD-A5DF**	0.921053
**TCGA-DD-AADB**	2.526316
**TCGA-DD-A39X**	1.394737
**TCGA-DD-AAE9**	2.289474
**TCGA-DD-A3A6**	0.5
**TCGA-MI-A75G**	5.078947
**TCGA-ED-A66X**	0.894737
**TCGA-2Y-A9H0**	1.657895
**TCGA-ZS-A9CE**	2.684211
**TCGA-G3-A25Y**	1.736842
**TCGA-DD-A114**	2.184211
**TCGA-DD-AAE7**	5.236842
**TCGA-5R-AAAM**	1.710526
**TCGA-CC-A7IJ**	3.184211
**TCGA-ED-A8O5**	2.421053
**TCGA-YA-A8S7**	1.473684
**TCGA-DD-A3A3**	1.184211
**TCGA-G3-A3CK**	4.763158
**TCGA-EP-A12J**	1.105263
**TCGA-CC-A3MB**	1.447368
**TCGA-BC-A10Z**	3.868421
**TCGA-DD-AACW**	1.052632

**TCGA-BD-A2L6**	1.710526
**TCGA-DD-A73C**	1.815789
**TCGA-ED-A97K**	1.078947
**TCGA-EP-A2KC**	1.605263
**TCGA-WQ-A9G7**	10.68421
**TCGA-G3-A3CG**	2.105263
**TCGA-BW-A5NQ**	2.157895
**TCGA-DD-A3A1**	4.026316
**TCGA-DD-AADE**	2.342105
**TCGA-DD-AACP**	3.631579
**TCGA-DD-AACG**	2.157895
**TCGA-DD-AACL**	6.315789
**TCGA-KR-A7K2**	1.078947
**TCGA-CC-5258**	2.763158
**TCGA-DD-A4NN**	1.105263
**TCGA-DD-A4NQ**	1.789474
**TCGA-DD-AAD5**	2.684211
**TCGA-DD-AACK**	3.105263
**TCGA-CC-A5UE**	2.868421
**TCGA-UB-A7MF**	2.236842
**TCGA-KR-A7K8**	1.026316
**TCGA-UB-AA0V**	0.789474
**TCGA-LG-A6GG**	3.973684
**TCGA-FV-A3R2**	1.631579
**TCGA-G3-A7M5**	3.947368
**TCGA-DD-AADV**	2.263158
**TCGA-DD-A1EI**	1.210526
**TCGA-DD-AACO**	0.921053
**TCGA-XR-A8TD**	1.184211
**TCGA-ED-A66Y**	1.921053
**TCGA-DD-A3A2**	1.578947
**TCGA-DD-A4NJ**	1.631579
**TCGA-DD-AACU**	2.447368
**TCGA-CC-A8HS**	1.157895
**TCGA-CC-A8HT**	4.5
**TCGA-DD-A39Y**	8.473684
**TCGA-G3-A5SJ**	2.210526
**TCGA-DD-A1EB**	3.447368
**TCGA-G3-AAV5**	1.578947
**TCGA-DD-A1EH**	0.842105
**TCGA-DD-A3A8**	2.815789
**TCGA-NI-A4U2**	2.526316
**TCGA-2Y-A9H4**	1.447368
**TCGA-DD-AADR**	2.026316
**TCGA-UB-AA0U**	1.736842
**TCGA-DD-AADO**	4.605263
**TCGA-2Y-A9GX**	1.078947
**TCGA-DD-AAE8**	1.342105
**TCGA-FV-A4ZQ**	1.473684
**TCGA-G3-A3CH**	0.868421
**TCGA-DD-AADU**	2.026316
**TCGA-ZS-A9CF**	4.526316
**TCGA-DD-A1EF**	2.473684
**TCGA-ZP-A9D1**	2.789474
**TCGA-CC-5263**	2.5
**TCGA-BC-A10T**	1.473684
**TCGA-DD-AAE2**	1.657895

**TCGA-G3-A3CI**	0.289474
**TCGA-DD-AADG**	3.552632
**TCGA-DD-AACE**	1.526316
**TCGA-DD-AADP**	2.184211
**TCGA-G3-AAV4**	2.078947
**TCGA-K7-AAU7**	1.578947
**TCGA-BC-A10W**	2.078947
**TCGA-2Y-A9H5**	1.763158
**TCGA-DD-AACZ**	3.710526
**TCGA-MI-A75H**	2.684211
**TCGA-BC-A3KF**	1.763158
**TCGA-HP-A5N0**	2.763158
**TCGA-DD-AADL**	2.763158
**TCGA-ED-A7PZ**	5.763158
**TCGA-UB-A7MB**	29.97368
**TCGA-2V-A95S**	2.026316
**TCGA-DD-AADK**	1.342105
**TCGA-GJ-A3OU**	1.210526
**TCGA-CC-A123**	1.657895
**TCGA-ZP-A9D2**	1.236842
**TCGA-EP-A2KA**	2.236842
**TCGA-DD-A73D**	1.473684
**TCGA-DD-A11B**	1.078947
**TCGA-DD-A73A**	2.236842
**TCGA-DD-AAVY**	2.026316
**TCGA-DD-AACV**	2.289474
**TCGA-FV-A23B**	1.789474
**TCGA-DD-AAE1**	1
**TCGA-MI-A75I**	3.763158

Supplementary Table S9. HPA database Immunohistochemical patient information.

Protein	Type	Sex	Age	Patient id	Antibody id	Staining
STC1	Normal	Female	63	3222	HPA023918	Not dected
	Tumor	Male	80	2280		Low
MAPT	Normal	Female	63	3222	HPA069570	Not dected
	Tumor	Female	70	5035		Medium
GAL	Normal	Male	67	1720	HPA049864	Not dected
	Tumor	Male	67	3447		Not dected
RAC3	Normal	Female	54	3402	HPA047820	Not dected
	Tumor	Female	58	2177		Not dected
HSP90AA1	Normal	Female	32	1846	CAB002058	Not dected
	Tumor	Male	24	1287		Low
HDAC1	Normal	Female	56	1846	CAB005017	Medium
	Tumor	Female	58	2177		High
GHR	Normal	Female	54	3402	HPA045339	High
	Tumor	Female	58	5032		High
CHGA	Normal	Female	79	516	CAB000023	Not dected
	Tumor	Female	52	812		Low

Supplementary Table S10. IC50 of liver cancer cell lines relative to drug sensitivity in GDSC database.

Cell line	Oxaliplatin	Cisplatin	Dabrafenib	Gefitinib	Afatinib
HLE	5.572622977	2.617914377	5.589237833	3.886492229	2.179657113
HuH-7	6.260676218	5.243869714	5.95635231	4.43919776	4.806842834
SK-HEP-1	5.191964931	4.355933645	4.196745719	3.885721269	3.11040054
SNU-387	5.841550386	5.825071535	6.342018398	4.39333317	2.347365271
SNU-423	6.182471378	3.643976717	6.13203162	4.370735716	2.059492266
SNU-449	4.918625114	4.965649763	6.194366351	3.22177575	1.975418575
C3A	3.97646907	3.168446657	6.430072942	4.512011612	3.603906756
JHH-2	6.225137593	4.290007598	5.304150836	4.08367688	1.393952302
JHH-4	6.002657717	3.091409792	4.0664886	4.467016627	2.034495286
JHH-6	5.172228037	5.727456379	5.569589486	4.008524051	3.570907437
JHH-7	6.298880266	4.592145887	4.750238857	4.645128736	3.497279151
SNU-398	4.163467429	4.603227293	5.160225097	3.886270874	1.256182984
huH-1	5.206773407	3.507875699	5.711500979	4.258492074	2.418996417
JHH-1	4.996642405	5.149275992	5.321767141	2.890108197	1.942145019

Supplementary Table S11. The relative expression level of NMIRG genes.

Cell		CT
HuH-7	β-actin	17.73
HuH-7	β-actin	17.28
HuH-7	β-actin	18.92
MHCC97-H	β-actin	21.02
MHCC97-H	β-actin	22.58
MHCC97-H	β-actin	22.46

Gene_name	HuH-7-CT	MHCC97-H-CT	HuH-7-ΔCT	MHCC97-H-ΔCT	ΔΔCT	2^(-ΔΔCT)
HDAC1	20.96	24.16	3.23	3.14	0.09	0.939522749
HDAC1	19.08	26.44	1.8	3.86	-2.06	4.169863043
HDAC1	19.83	25.86	0.91	3.4	-2.49	5.617779503
CHGA	25.8	35.04	8.07	14.02	-5.95	61.81992505
CHGA	24.86	35.43	7.58	12.85	-5.27	38.58585049
CHGA	26.99	34.52	8.07	12.06	-3.99	15.88947993
STC1	22.98	28.46	5.25	7.44	-2.19	4.563054863
STC1	22.52	27.88	5.24	5.3	-0.06	1.042465761
STC1	21.53	28.44	2.61	5.98	-3.37	10.33882265
GAL	19.05	28.75	1.32	7.73	-6.41	85.0358921
GAL	20.11	29.59	2.83	7.01	-4.18	18.12614216
GAL	19.82	29.76	0.9	7.3	-6.4	84.44850629
HSP90AA1	22.05	29.64	4.32	8.62	-4.3	19.69831061
HSP90AA1	21.09	31.27	3.81	8.69	-4.88	29.44600482
HSP90AA1	21.87	30.32	2.95	7.86	-4.91	30.06472797
BTC	22.42	32.01	4.69	10.99	-6.3	78.79324245
BTC	22.76	32.26	5.48	9.68	-4.2	18.37917368
BTC	22.58	31.8	3.66	9.34	-5.68	51.26847217
GHR	19.62	30.85	1.89	9.83	-7.94	245.5716145
GHR	18.51	31.62	1.23	9.04	-7.81	224.4110647
GHR	19.1	28.61	0.18	6.15	-5.97	62.68289905
MAPT	25.7	32.09	7.97	11.07	-3.1	8.5741877
MAPT	27.14	32.7	9.86	10.12	-0.26	1.197478705
MAPT	26.79	32.35	7.87	9.89	-2.02	4.055837919
RAC3	23.32	26.96	5.59	5.94	-0.35	1.274560627
RAC3	20.14	27.32	2.86	4.74	-1.88	3.680750602
RAC3	22.28	27.4	3.36	4.94	-1.58	2.989698497
